# Hydrogen‐Bond Network Determines the Early Photoisomerization Processes of Cph1 and AnPixJ Phytochromes

**DOI:** 10.1002/anie.202104853

**Published:** 2021-07-16

**Authors:** Xiang‐Yang Liu, Teng‐Shuo Zhang, Qiu Fang, Wei‐Hai Fang, Leticia González, Ganglong Cui

**Affiliations:** ^1^ Key Laboratory of Theoretical and Computational Photochemistry Ministry of Education College of Chemistry Beijing Normal University Beijing 100875 China; ^2^ College of Chemistry and Material Science Sichuan Normal University Chengdu 610068 China; ^3^ Institute of Theoretical Chemistry Faculty of Chemistry University of Vienna Währinger Straße 17 1090 Vienna Austria

**Keywords:** CASPT2, CASSCF, photochemistry, photoreceptors, QM/MM

## Abstract

Phytochrome proteins are light receptors that play a pivotal role in regulating the life cycles of plants and microorganisms. Intriguingly, while cyanobacterial phytochrome Cph1 and cyanobacteriochrome AnPixJ use the same phycocyanobilin (PCB) chromophore to absorb light, their excited‐state behavior is very different. We employ multiscale calculations to rationalize the different early photoisomerization mechanisms of PCB in Cph1 and AnPixJ. We found that their electronic S_1_, T_1_, and S_0_ potential minima exhibit distinct geometric and electronic structures due to different hydrogen bond networks with the protein environment. These specific interactions influence the S_1_ electronic structures along the photoisomerization paths, ultimately leading to internal conversion in Cph1 but intersystem crossing in AnPixJ. This explains why the excited‐state relaxation in AnPixJ is much slower (ca. 100 ns) than in Cph1 (ca. 30 ps). Further, we predict that efficient internal conversion in AnPixJ can be achieved upon protonating the carboxylic group that interacts with PCB.

## Graphical abstract

QM/MM calculations reveal that hydrogen‐bond networks interacting with the PCB chromophore govern the distinct excited‐state relaxations of red‐light photoreceptors Cph1 and AnPixJ. The present results contribute to decipher the natural evolution of both cyanobacteriochromes and phytochromes.

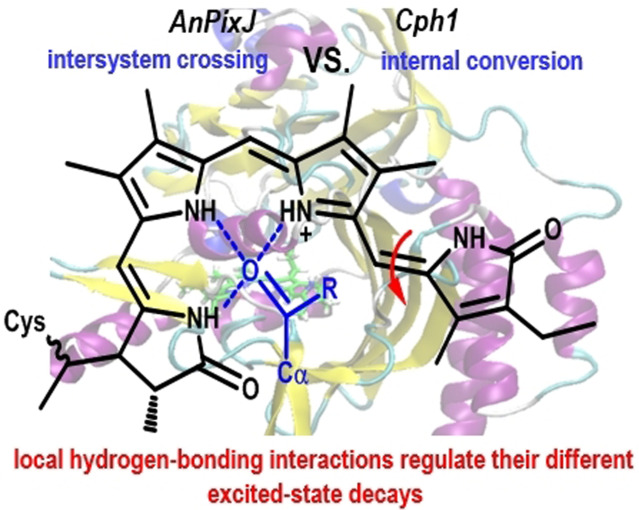

## Introduction

**Figure 1:**
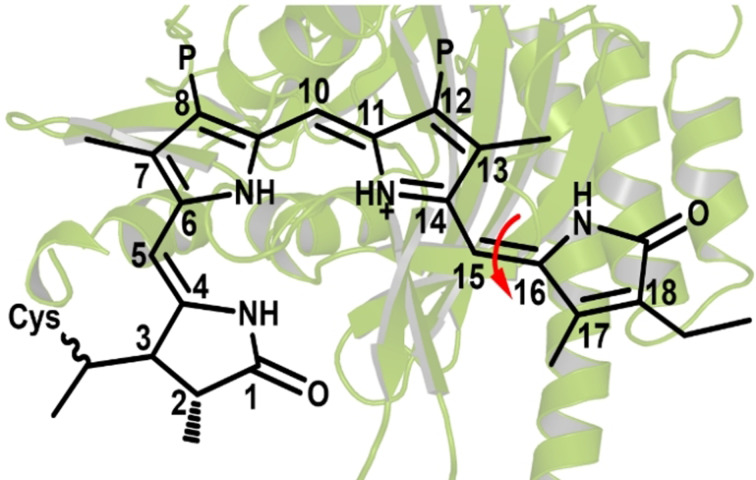
Phycocyanobilin (PCB) chromophore linked to a conserved cysteine residue via a covalent thioether bond within the GAF domain. In both Cph1 and AnPixJ, a Asp residue is hydrogen‐bonded with the chromophore but differently: In AnPixJ, the side‐chain COO^−^ group is bonded, while in Cph1, the backbone C=O group is bonded (see Figures S1 and S2 for local protein surroundings of the PCB chromophore in Cph1 and AnPixJ).

Phytochromes are among the most important photosensory proteins, being widely present in plants, bacteria, cyanobacteria, and fungi.^[1]^ They are a superfamily of dimeric chromoproteins that absorb light by virtue of a covalently bound bilin chromophore. Upon illumination, the red‐absorbing dark‐adapted state of phytochrome (Pr) converts reversibly into the far‐red‐absorbing (Pfr) active signaling state.^[1]^ This Pr/Pfr photocycle is driven by a *Z‐E* photoisomerization of the C15=C16 double bond in the bilin chromophore, buried within the GAF domain (see Figure 1).[^1^, ^2^] Phytochrome's ability to sense ambient light environments as well as to regulate numerous photoresponses in plants and microorganisms is attributed to the underlying photoinduced inter‐conversion.^[1]^

Cyanobacteriochromes belong to another family of phytochrome‐related photoreceptor proteins, discovered in cyanobacteria.[^3a^, ^3b^] They exhibit diverse photocycles in the visible to near‐ultraviolet spectral range but have a much simpler domain architecture compared to phytochromes.^[3]^ High‐resolution crystal structures of cyanobacteriochromes confirm that they share key structural residues with phytochromes and that their photocycles are similar.[^2^, ^4^] Both phytochromes and cyanobacteriochromes indeed use bilin chromophores to regulate the photoresponse and employ the same *Z*‐*E* photoisomerization around the C15=C16 double bond to drive the photocycle. However, their excited‐state dynamics is drastically different.^[5]^ The Pr form of cyanobacterial phytochrome Cph1 isomerizes with a time constant of ca. 30 ps,[^5b^, ^5c^, ^5d^, ^5e^] whereas, the best characterized cyanobacteriochrome AnPixJ exhibits a time constant of ca. 100 ns.^[5a]^ Unfortunately, the physical origins of these contrasting photoisomerization dynamics remain elusive. It is the purpose of this work to clarify the reasons for this different behavior.

As illustrated in one previous work on the Pr form of a bacteriophytochrome, steric interactions with the environment play a key role in triggering different reaction channels.^[6]^ There is a strong binding of the chromophore to the protein, and the isolated in‐vacuum chromophore cannot undergo photoreversible conversion. However, most of the previous studies on the phycocyanobilin (PCB) chromophore did not consider the protein surroundings.^[7]^ Furthermore, the photoresponse mechanism of cyanobacteriochromes is still largely unexplored computationally, except for absorption spectra calculations in Slr1393g3 that identified the physical origin of its spectral tuning.^[8]^ Quantum mechanics/molecular mechanics (QM/MM) calculations of excited state relaxation pathways are just beginning to emerge.^[9]^ And yet, there is no related computational study that ever compared the photochemistry of phytochromes and cyanobacteriochromes.

Here, we exploit a multiscale QM/MM approach to illuminate the *Z*‐*E* photoisomerization of PCB including the cyanobacterial phytochrome Cph1 and cyanobacteriochrome AnPixJ protein environments. This allows us to rationalize the nature of the excited‐state decay channels, the role of nonradiative conical intersections, the involvement of triplet states, and ultimately the reasons for the different excited state dynamics of both phytochromes in its natural protein environments.

## Results and Discussion

The computations were based on the crystal structures of Cph1 (PDB code: 2VEA^[2d]^) and AnPixJ (PDB code: 3W2Z^[4a]^). The geometries were solvated, neutralized and equilibrated with molecular dynamics simulations, from which snapshots were selected to perform the QM/MM calculations; see Section S1 of the Supporting Information for further details.

**Figure 2:**
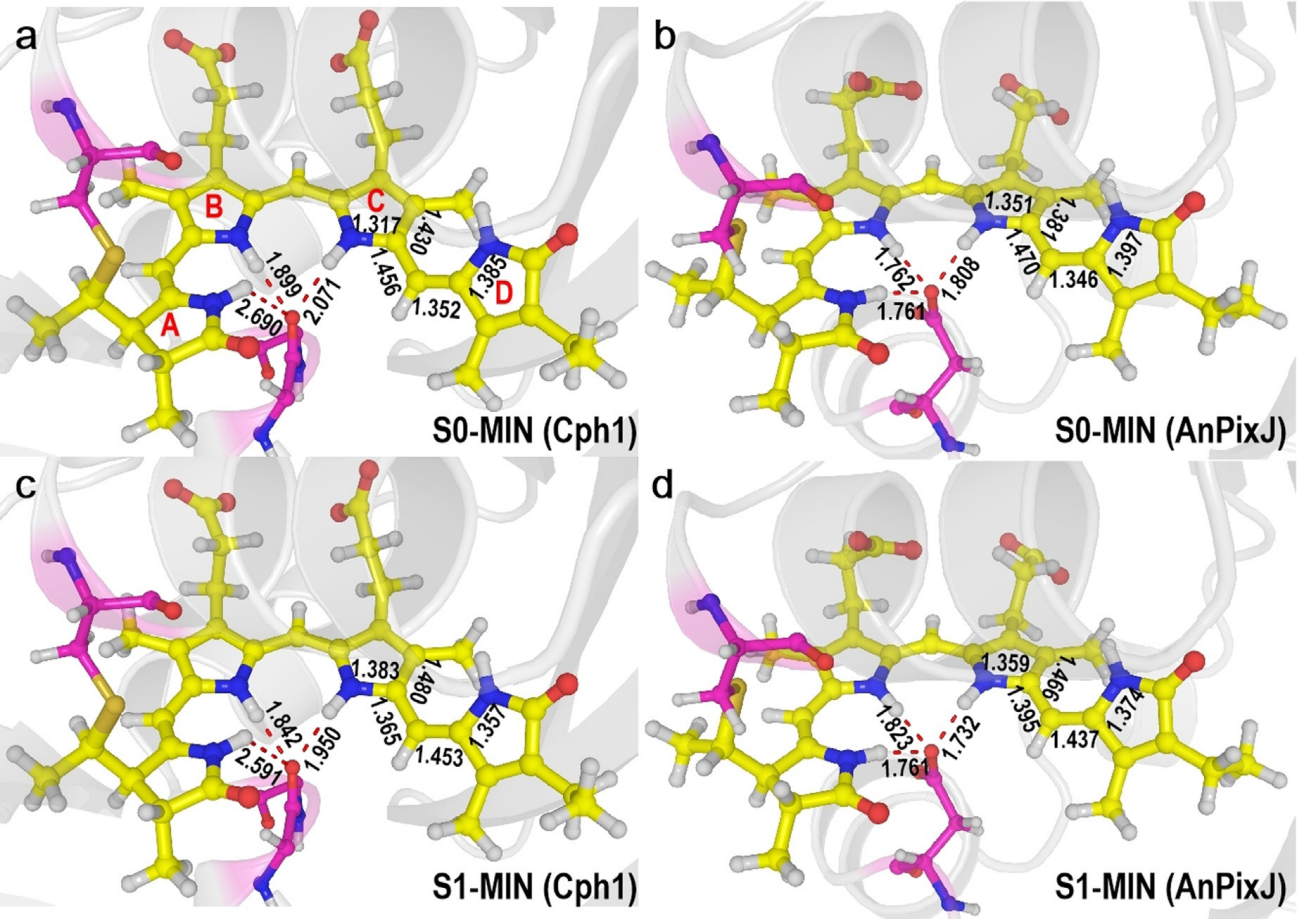
QM(CASSCF)/MM optimized S_0_ and S_1_ minimum‐energy structures of Cph1 (left, panel a and c) and AnPixJ (right, panel b and d). Selected bond lengths in Angstroms. See the Supporting Information for QM/MM setup and Cartesian coordinates of QM regions.

The ground state (S_0_) minimum structures of Cph1 and AnPixJ optimized at the QM(CASSCF)/MM level of theory are depicted in Figure 2 (see the active spaces used in CASSCF calculations in Figures S3–S4). An analysis of the bond lengths shows that the NH^+^ group should be primarily assigned to the C pyrrole ring of PCB in Cph1 but to the B pyrrole ring in AnPixJ. This is also supported by the largest positive charge found in the C and B pyrrole rings of Cph1 and AnPixJ, see Tables S1 and S2, respectively. This difference is due to different hydrogen‐bonding interactions of PCB with the nearby Asp residue involving the neutral carbonyl group in Cph1 and the negatively charged carboxylic group in AnPixJ (see Figures S1 and S2). Accordingly, the valence bond pattern of the B and C pyrrole rings differs in Cph1 and AnPixJ, ultimately leading to dissimilar excited‐state structures, isomerization, and decay dynamics, as it will be discussed below (Figure S14).

The calculated QM(CASPT2)/MM vertical excitation energies to the S_1_ state at these optimized S_0_ minima are 43.9 and 47.6 kcal mol^−1^ for Cph1 and AnPixJ (Table S3)—in close agreement to the experimental values of 43.3 and 44.1 kcal mol^−1^, respectively.[^2a^, ^2b^, ^3c^] An electronic structure analysis shows the S_1_ electronic excited state at the Franck‐Condon region is dominantly caused by a local electronic excitation in both Cph1 and AnPixJ (Table S4). However, the relaxed S_1_ state geometry of the PCB chromophore changes remarkably with respect to the S_0_ (see Figure 2 c,d). From the S_0_ to S_1_ minima the main changes are in the bond lengths of the C and D pyrrole rings of Cph1 (N_C_‐C14‐C15‐C16‐N_D_) and in the B, C, and D pyrrole rings of AnPixJ (C6‐N_B_‐C9‐C10‐C11‐C12‐C13‐C14‐C15‐C16‐N_D_). As a result, the C14−C15 single bond and C15=C16 double bond in the S_0_ state change to double and single bonds in the S_1_ states of both Cph1 and AnPixJ, respectively. The corresponding BLA indexes for C14−C15 and C15−C16 are calculated to be −0.091 Å, and 0.101 Å for Cph1 and −0.075 Å and 0.091 Å for AnPixJ. It is also interesting that compared to the S_0_ electronic structure at the S_0_ minima, there is a significant multireference character in the S_1_ electronic structure at the S_1_ minima, with two comparable contributions: from both the closed‐shell and charge‐transfer electronic configurations (Table S4).

**Figure 3:**
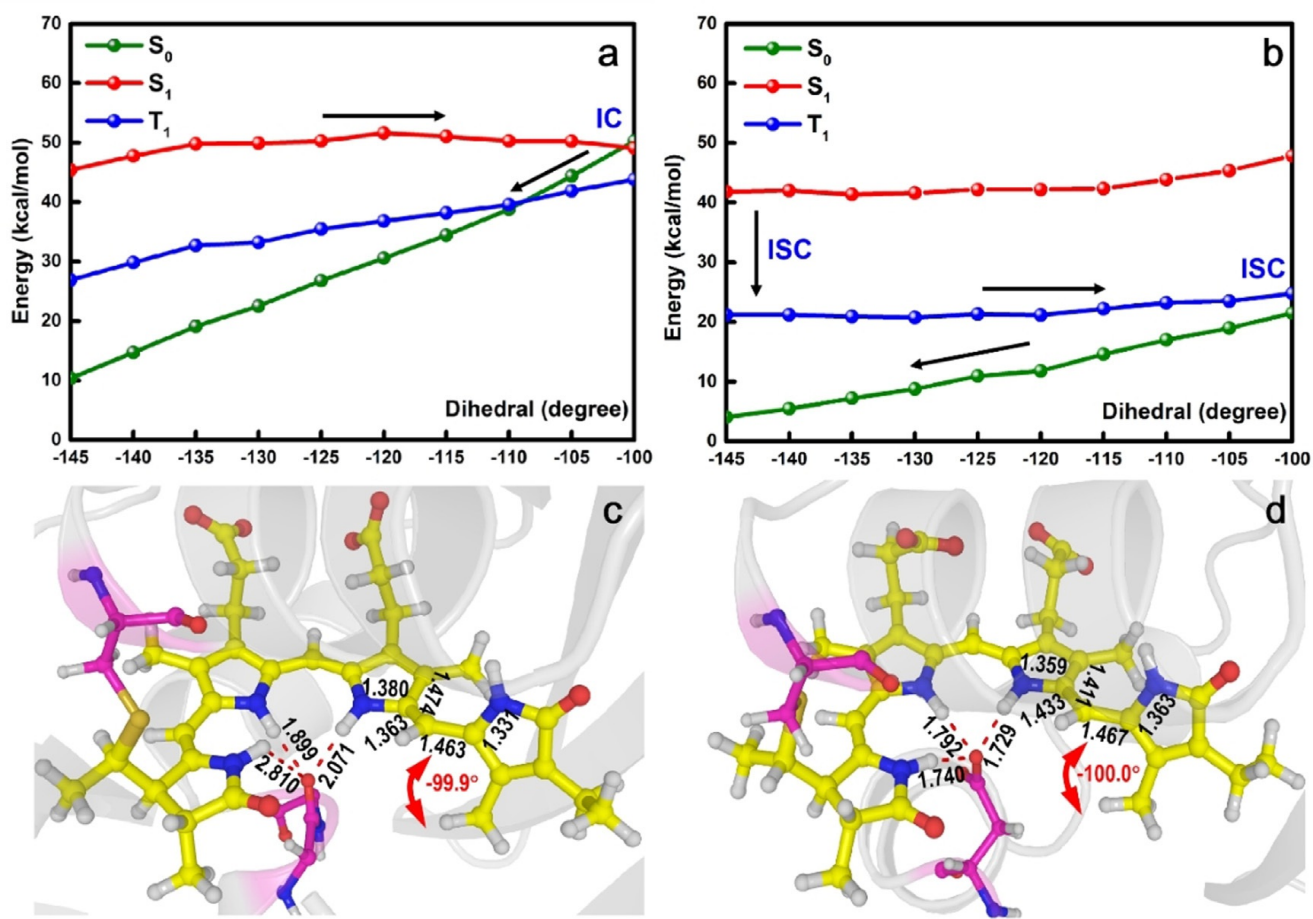
QM(CASPT2)/MM isomerization paths along the C14‐C15‐C16‐C17 dihedral angle of PCB in Cph1 (a) and AnPixJ (b) calculated at the optimized S_1_ minimum‐energy for each rotation angle. Also shown are the S_1_/S_0_ structure (energy gap 1.2 kcal mol^−1^) of Cph1 (c) and the S_1_ structure of AnPixJ (d) at the C14‐C15‐C16‐C17 dihedral angle of ca. −100°.

As suggested experimentally,^[4]^ the decay of Cph1 and AnPixJ after light irradiation is triggered by isomerization around the C15=C16 double bond between the C and D pyrrole rings of PCB. Therefore, we calculated the corresponding minimum‐energy paths in the S_1_ state along the decisive C14‐C15‐C16‐C17 dihedral angle (Tables S5–S10). The S_1_ potential energy surface in Cph1 (Figure 3 a) is quite flat and is associated with a barrier of only 6.2 kcal mol^−1^. In the vicinity of −100°, the S_1_ state becomes energetically degenerate with the S_0_ state, indicating the presence of an S_1_/S_0_ conical intersection. Thus, around this geometry, PCB in Cph1 is expected to undergo a fast S_1_→S_0_ internal conversion. The T_1_ and S_0_ states also intersect at a dihedral angle of about −110°; however, this excited‐state decay path will be less efficient because intersystem crossing from S_1_ to T_1_ is slow according to the El‐Sayed rule (same electronic state character, see Table S4).^[10]^ We can therefore conclude that internal conversion via the S_1_/S_0_ conical intersection along the photoisomerization reaction is responsible of the excited‐state decay of Cph1. Given the small barrier associated with this process, it is expected to be fast, which is consistent with the experimentally measured time constants of ca. 30 ps.[^5b^, ^5c^, ^5d^, ^5e^] This is further supported by 1 ps nonadiabatic dynamics simulations carried out with the QM(OM2/MRCI)/MM method using an active space of 16 electrons in 14 orbitals (see Supporting Information for simulation details). Figure S16 shows no hopping events to the electronic ground state within 1 ps, consistent with the existence of a barrier of 6.2 kcal mol^−1^.

In contrast, the excited‐state decay of AnPixJ is experimentally much slower.^[5a]^ From Figure 3 b we see that although the S_1_ and S_0_ energies gradually increase, they remain well separated energetically along the photoisomerization path, and there is no hint of an S_1_/S_0_ conical intersection that could promote fast S_1_→S_0_ internal conversion. Instead, around 100° the T_1_ energy gets close to the S_0_ one and the T_1_ state should thus be able to decay to the S_0_ state after being formed through an S_1_→T_1_ intersystem crossing from the S_1_ state. However, as mentioned above, this intersystem crossing is not efficient since the SOC values are smaller than 1 cm^−1^, and thus the entire excited‐state relaxation process becomes very slow compared with that in Cph1. This could explain the experimental observation that excited‐state relaxation in AnPixJ takes ca. 100 ns.^[5a]^

In order to get insight into the physical origin of the distinct excited‐state decay dynamics of PCB in Cph1 and AnPixJ, we have analyzed the electronic structure of the corresponding S_1_ states along the photoisomerization paths (Table S4). We find that the S_1_ electronic structure of Cph1 is of charge‐transfer character and remains almost constant during the photoisomerization, being essentially the same as at the S_1_ minimum and at the S_1_/S_0_ conical intersection. Both structures exhibit similar valence‐bond patterns (Figures 2 c and 3 c), with the C14−C15 and C15−C16 bonds being of double‐ and single‐bond characters, respectively; this is true along the entire photoisomerization path (Figure S5). The C15−C16 bond length does not change much (1.453 Å at the S_1_ minimum vs. 1.463 Å at the S_1_/S_0_ conical intersection). Consequently, the rotation around the C15−C16 single bond is rather straightforward with a small barrier in the S_1_ state. Noteworthy, similar excited‐state isomerization paths were found in the isolated PΦB chromophore in vacuum (PCB in this work).^[7h]^ However, relevant energy barriers are different (6.2 kcal mol^−1^ in PCB versus 0.7 kcal mol^−1^ in PΦB) and the roles of triplet states and local hydrogen‐bonding network were not studied in the previous work.

In contrast, in AnPixJ, the S_1_ electronic structure at the C14‐C15‐C16‐C17 dihedral angle of −100° is different from that at the S_1_ minimum. We recall that the S_1_ state is of charge‐transfer character at its minimum, and the valence bond patterns of the B, C, and D pyrrole rings are different from those at the S_0_ minimum (Figure 2). The C14−C15 and C15−C16 bonds exhibit typical double‐ and single‐bond characters at the S_1_ minimum, respectively. However, the S_1_ electronic structure varies along the photoisomerization path. Closer examination reveals that the charge‐transfer electronic configuration is dominant in the early phase of the photoisomerization, whereas more charge‐transfer electronic configurations are involved in the S_1_ state and becomes dominant when the C14‐C15‐C16‐C17 dihedral angle proceeds beyond −130° (see Figure 3 b and discussion in Table S4). In these configurations, there are molecular orbitals with clear antibonding character for the C14=C15 double bond, which makes it longer (Figure S5), as also found in vacuum in ref. [7h]. For these reasons, the corresponding S_1_ energy along the photoisomerization path increases slightly compared with that in Cph1. Nevertheless, this small difference of the S_1_ potential energy surfaces in Cph1 and AnPixJ is not responsible for their distinct excited‐state decay dynamics.

Instead, we attribute the different excited‐state decay dynamics of Cph1 and AnPixJ to their distinct S_0_ potential energy surfaces along the photoisomerization reaction (Figure 3). While in Cph1, the S_0_ energy is drastically lifted approaching the S_1_ state to form an S_1_/S_0_ conical intersection, the S_0_ energy of AnPixJ gradually increases but still far away from the S_1_. These differences can be rooted to their distinct S_0_ electronic structures along the photoisomerization paths. In Cph1, the S_0_ state is primarily composed of closed‐shell and charge‐transfer electronic configurations in the whole process. In contrast, in AnPixJ, the S_0_ electronic structure is similar to that of Cph1 in the first stage, whereas, in the late stage, the charge‐transfer configuration is replaced by a double‐excitation one, whose weight is even comparable with the closed‐shell one at the S_1_ structure with the C14‐C15‐C16‐C17 of −100° (see Table S4).

**Figure 4:**
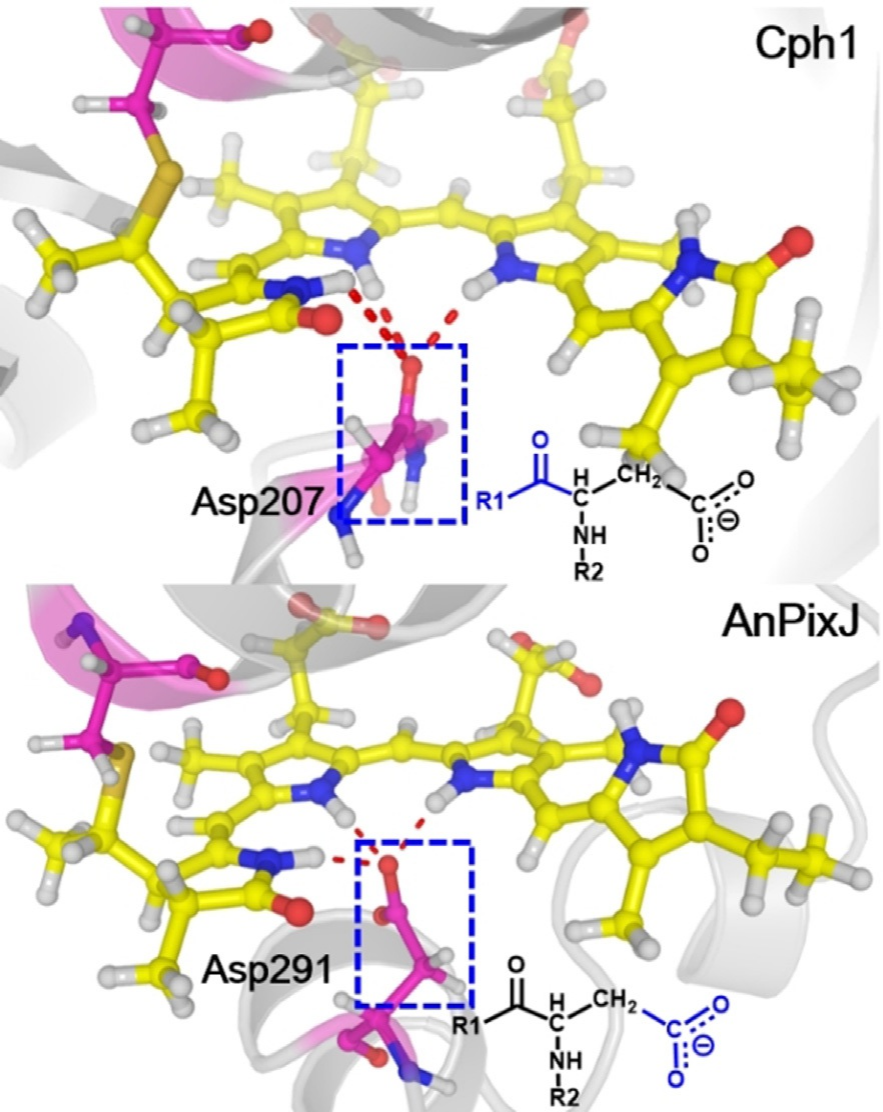
Local hydrogen‐bonding networks near the PCB chromophore in Cph1 and AnPixJ. Different parts of the Asp residue (in blue) are hydrogen‐bonded with the chromophore.

Why do the electronic structures of the PCB chromophore evolve so differently along the photoisomerization paths of Cph1 and AnPixJ? We propose that one possible factor that could regulate the S_1_ and S_0_ electronic structures is the hydrogen‐bonding interaction with the nearby Asp residue involving its neutral carbonyl group in Cph1 and its negatively charged carboxylic group in AnPixJ (Figure 4 and Figure S13). To investigate this hypothesis further, we constructed an artificial AnPixJ variant in which the carboxylic group is protonated to form a neutral [‐COOH] group (Section S3). The optimized S_0_ and S_1_ minima of this variant have electronic structures similar to those in AnPixJ (see Figure S6). However, the computed photoisomerization path (Figure S7) is different and actually resembles the one in Cph1 in terms of the energetics as well as bond lengths and most importantly: there is an energetically accessible S_1_/S_0_ conical intersection that controls the excited‐state relaxation dynamics of this mutant form. At the S_1_/S_0_ conical intersection, the C14−C15 and C15−C16 bonds are of typical double‐ and single‐bond characters (see Figure S7), as in the case of Cph1 (Figure 3 c) and consequently, the photoisomerization around the C15−C16 bond becomes quite easy. These comparisons emphasize the importance of the charge state of the chemical group interacting with the chromophore in governing the excited‐state relaxation dynamics.

Both Cph1 and AnPixJ proteins use the conserved Asp residue to interact with the chromophore, but the hydrogen‐bonding network is different due to their different residue sequences. Accordingly, different chemical groups of this residue are hydrogen‐bonded with the chromophore, in particular a ‐C=O group in Cph1 and a ‐COO^−^ group in AnPixJ (see Figure 4). We pinpoint this difference as the key reason that eventually triggers the distinct excited‐state decay dynamics of Cph1 and AnPixJ.

The protein environmental effects are further investigated by constructing cluster models including only the PCB chromophore and the nearby Asp residue (see Section S4). Interestingly, the optimized S_0_ and S_1_ minima (Figure S8) as well as the minimum‐energy photoisomerization paths are similar to those in Cph1 and AnPixJ (see Figures S9–S11). This reveals that in this case the environmental effects involving other residues play a minor role.

**Figure 5:**
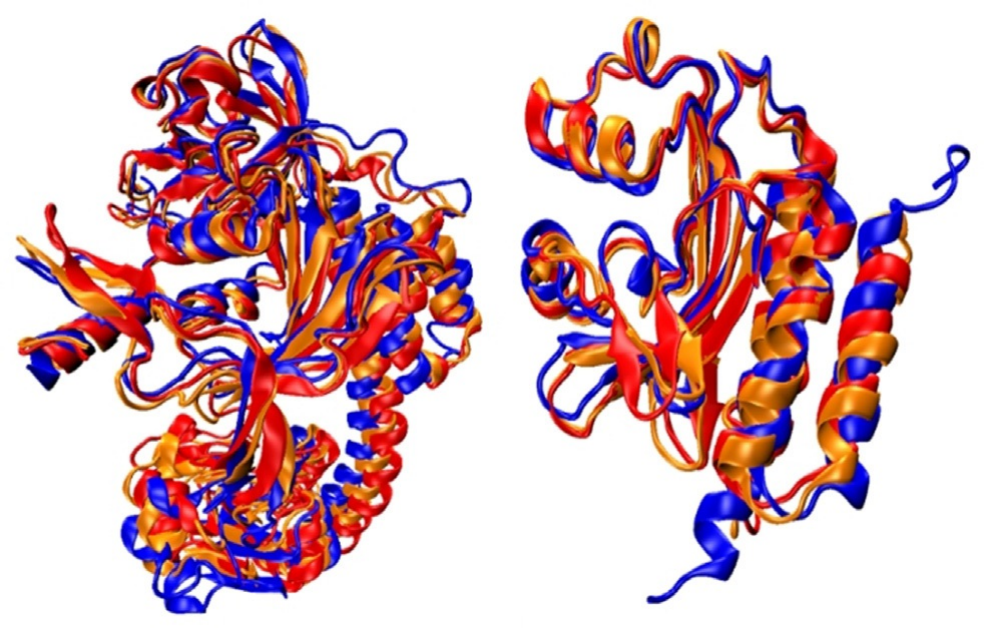
Spatial overlaps of the (left) Cph1 and (right) AnPixJ proteins of MD snapshots at 1 ns (blue), 100 ns (red), and 200 ns (orange).

Finally, in order to investigate protein fluctuation effects, an extended 200 ns MD simulation was carried out. Figure 5 evidences that the protein structures and related hydrogen‐bond networks are stable, as the MD snapshots at 1 ns, 100 ns, and 200 ns nicely overlap. Moreover, we have re‐optimized the S_1_ minimum‐energy isomerization paths of Cph1 and AnPixJ at the QM(OM2/MRCI)/MM level using the 100 ns MD snapshots as the initial structures. The photoisomerization paths are the essentially same as those using 1 ns MD snapshot (Figure S15). These results demonstrate that the protein fluctuation will not change the proposal that the hydrogen‐bond networks determine the early photoisomerization processes of Cph1 and AnPixJ phytochromes.

## Conclusion

We used accurate multi‐scale QM(CASPT2)/MM calculations to investigate the early PCB photoisomerization in Cph1 and AnPixJ with atomistic detail. We found that the S_1_, T_1_, and S_0_ minima exhibit distinct geometric and electronic structures because of individual hydrogen‐bonding networks with the nearby Asp residue. These networks also regulate the S_1_ electronic structures along the photoisomerization paths and ultimately lead to different excited‐state behaviors in Cph1 and AnPixJ. In the former, the S_1_ charge transfer character is maintained along the entire photoisomerization path, which provides access to an S_1_/S_0_ conical intersection that allows an efficient S_1_→S_0_ internal conversion to the S_0_ state. In the latter, the S_1_ electronic structure along the computed path evolves from initial charge transfer to diradical characters in the later phase, which prevents the formation of an S_1_/S_0_ conical intersection so that the excited‐state decay will be less efficient (involving consecutive S_1_→T_1_ and T_1_→S_0_ intersystem crossings to the S_0_ state). These different excited‐state dynamics rationalize the experimental observations that excited‐state relaxation is slow (ca. 100 ns) in AnPixJ but fast (ca. 30 ps) in Cph1.^[5]^ We also found that protonation of the carboxylic group in AnPixJ will recover efficient S_1_→S_0_ internal conversion via an S_1_/S_0_ conical intersection‐underscoring the decisive influence of the protonation state of nearby residues on the excited‐state dynamics of biological chromophores. In general, our findings demonstrate the feasibility of multi‐scale QM/MM calculations to provide exquisite insight into the photoresponse of red‐light photoreceptors to understand the natural evolution of phytochromes and cyanobacteriochromes.

## Conflict of interest

The authors declare no conflict of interest.

## Supporting information

As a service to our authors and readers, this journal provides supporting information supplied by the authors. Such materials are peer reviewed and may be re‐organized for online delivery, but are not copy‐edited or typeset. Technical support issues arising from supporting information (other than missing files) should be addressed to the authors.

Supporting Information
