# Supplementary material for: Hydrogen‐Bond Network Determines the Early Photoisomerization Processes of Cph1 and AnPixJ Phytochromes
Source: Angew Chem Int Ed Engl. 2021 Jul 16;60(34):18688–93. doi: 10.1002/anie.202104853 (PMC8456922; doi:10.1002/anie.202104853)
Supplement: Supplementary file 1 — Supporting Information [file ANIE-60-18688-s001.pdf]

Angewandte Chemie
Eine Zeitschrift der Gesellschaft Deutscher Chemiker

Supporting Information

Hydrogen-Bond Network Determines the Early Photoisomerization Processes of Cph1 and AnPixJ Phytochromes

Xiang-Yang Liu, Teng-Shuo Zhang, Qiu Fang, Wei-Hai Fang, Leticia González, and Ganglong Cui**

anie_202104853_sm_miscellaneous_information.pdf

Supporting Information

Contents

1.1 System Setup 3

1.2 QM/MM Calculations 3

1.3 Nonadiabatic Dynamics Simulations 5

1.4 Active Spaces of the QM region 8

1.4.1 Cph1 8

1.4.2 AnPixJ 9

2. Relevant Bond Lengths along Photoisomerization Path 10

3. AnPixJ-COOH 10

3.1 Optimized Minimum-Energy Structures 10

3.2 Optimized Minimum-Energy Path 10

4. Gas Phase Calculations 11

4.1 Gas Phase Optimized Minimum-Energy Structures 11

4.2 Gas Phase Optimized Minimum-Energy Paths 12

5. T_1 Minimum-Energy Structures 13

6. Hydrogen-Bonding Networks 13

7. Approximated Valence Bond Patterns 14

8. OM2/MRCI Optimized Minimum-Energy Paths 15

9. Nonadiabatic Dynamics Simulations 15

10. Tables 16

11. References 32

12. Cartesian Coordinates of All Optimized Structures (QM Part) 38

Computational Details

1.1 System Setup

Initial geometries of Cph1 and AnPixJ were constructed from available X-ray structures (Cph1, PDB ID: 2VEA; AnPixJ, PDB ID: 3W2Z).[1,2] The crystal structure of Cph1 consists of 500 amino acids, 29 crystal waters, and a phycocyanobilin (PCB) chromophore; it was solvated in a rectangular TIP3P water box of 100x95x120 Å^3. The crystal structure of AnPixJ consists of 178 amino acids, 185 crystal waters, and the same PCB chromophore; it was placed into a rectangular TIP3P water box of 80x80x70 Å^3. The protonation states of ionizable groups of all residues were set according to pH 7.0, and the protonation states of histidine residues were defined in analogy to previous work.[3,4] Both systems were neutralized by adding sodium ions on the protein surface near negatively charged residues. These setups were done using the tleap module in AMBER2015.[5]

Subsequently, equilibrium molecular dynamics (MD) simulations were carried out. In the first stage both systems were restrained with force constants of 100.0 kcal*mol^{-1}*Å^{-2}, and only sodium ions and water molecules were minimized for 10000 steps. Thereafter, the systems were minimized (20000 steps), heated (20 ps, NVT, T=300 K), and equilibrated (200 ns, NPT, T=300 K, P=1 atm) without any geometric restraints. The Andersen temperature coupling scheme and periodic boundary conditions were used during these MD calculations. [6] Nucleic acids and sodium ions were described with the built-in amber ff14SB force-field parameters;[7] water molecules were described with the TIP3P model;[8] and the PCB chromophore was described using the generalized Amber force field.[9] All these calculations were performed using the sander module in AMBER2015.[5] To save computational time, the 200 ns MD simulations are finished using highly parallel computer resources of the supercomputing center of the Beijing PARATERA Tech. Corp., Ltd.

1.2 QM/MM Calculations

The final snapshots of the above equilibrium MD simulations were taken as the starting structures for the following QM/MM calculations. The QM region included: (1) the truncated PCB chromophore, with the side chains, e.g. $-CH_3$, $-CH_2-CH_3$, and $-CH_2-CH_2-COO^-$, being placed into the MM region to save computational effort; (2) the groups of the Asp residues of Cph1 and

AnPixJ that interact with the chromophore through hydrogen-bonding interactions, specifically, -CH_2-C(O)-NH-CH_2- in Cph1 and -CH_2-COO^- in AnPixJ (see Figures S1-S2). All unsaturated bonds generated due to the truncation were saturated with hydrogen atoms. The remainder of the system was included in the MM region.

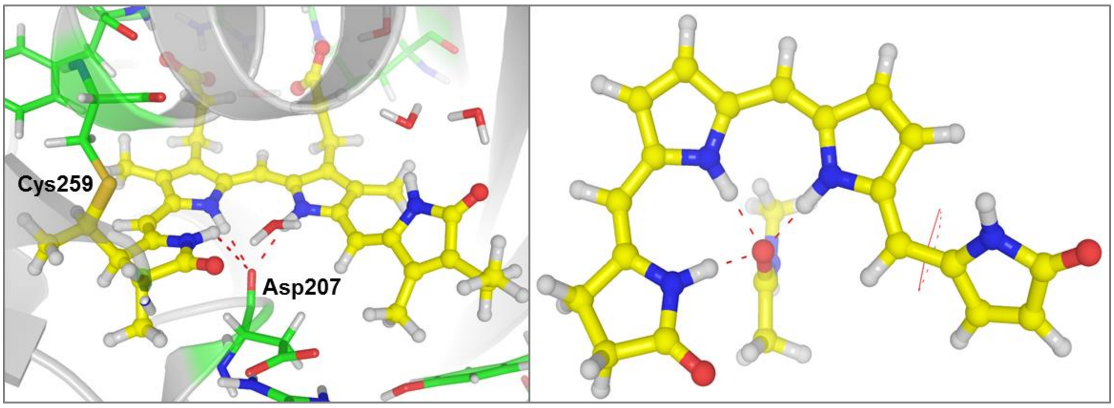

Figure S1. Cartoon of the QM/MM system of Cph1 (left) and QM region included in the QM/MM calculations (right).

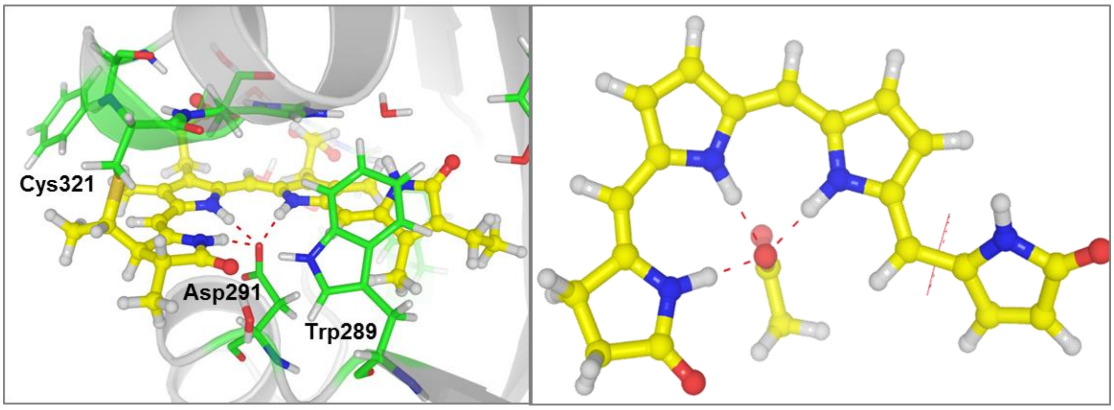

Figure S2. Cartoon of the QM/MM system of AnPixJ (left) and QM region included in the QM/MM calculations (right).

The QM regions were treated using the state-averaged complete-active-space self-consistent field (CASSCF) method and subsequently, the second-order perturbation approach (CASPT2)[10,11] on top. In the CASSCF calculations, an active space of 10 electrons in 8 orbitals was employed in combination with the 6-31G* basis set.[12,13] This choice was proven to be useful in previous theoretical investigations of excited states of biological systems.[14-18] In the CASPT2 calculations, a larger active space of 14 electrons in 12 orbitals was used in combination with the larger cc-pVTZ basis set.[19] In addition, the Cholesky decomposition technique with unbiased auxiliary basis sets was applied for accurate two-electron integral approximation;[20] the

imaginary shift technique (0.2 a.u.) was used to avoid intruder-state issues;[21] and the ionization potential electron affinity shift was set to zero.[22,23] The MM region was described with the amber ff14SB force field and the TIP3P model as specified above (section 1.1).[7,8] The QM-MM boundary was treated by the hydrogen link-atom scheme.[24] The electronic embedding scheme was used to account for the electrostatic interactions between the QM and MM regions.[25] In all QM/MM computations, the MM atoms within 15 Å from the center of mass of the PCB chromophore were allowed to move, while the other more distant atoms were frozen at their positions at end of the MD simulations.

The following computer codes were used: QM/MM calculations by ChemShell3.5;[26-28] CASSCF calculations by GAUSSIAN03;[29] CASPT2 calculations by MOLCAS8.0;[30,31] and MM calculations by the DL_POLY module implemented in ChemShell3.5.[32]

For comparison, we conducted QM calculations on gas-phase cluster models consisting of the QM regions used in the QM/MM calculations. We optimized all relevant structures and minimum-energy reaction paths using the same CASSCF approach as in the QM/MM calculations (see above). For the CASPT2 calculations on gas-phase cluster models, we employed the same options as before (see above) but used the smaller 6-31G* basis set.

1.3 Nonadiabatic Dynamics Simulations

Trajectory-based semi-classical nonadiabatic dynamics simulation approaches with different hopping schemes have been employed to simulate ultrafast excited-state relaxation processes in a lot of chemical and biological systems as well as materials.[33-47] In the present work, trajectory-based surface-hopping dynamics simulations involving the lowest two singlet states (S_1 and S_0) are carried out with the recently developed scheme proposed by Zhu et al.[48] In this approach, the system is only propagated in an electronic state at any time; however, it can jump to another potential energy surface near quasi-degenerate regions. The non-adiabatic transition probability is computed according to the Landau-Zener formula improved by Zhu and Nakamura [49–51]

$$p = \exp\left(-\frac{\pi}{4\sqrt{a^2}}\sqrt{\frac{2}{b^2+\sqrt{|b^4 \pm 1|}}}\right) \tag{1}$$

in which two unitless parameters, i.e. effective coupling and collision energy, are written as

$$a^2 = \frac{\hbar^2}{2\mu}\frac{\sqrt{|F_2F_1|}|F_2 - F_1|}{(2V_{12})^3} \tag{2}$$

and

$$b^2 = (E_t - E_x)\frac{|F_2 - F_1|}{\sqrt{|F_2F_1|}(2V_{12})} \tag{3}$$

where F_1 and F_2 are the two mass-scaled one-dimensional diabatic forces; V_{12} is the diabatic coupling; μ is the reduced mass; E_x is the energy at the crossing point, and E_t is the potential energy plus kinetic energy component along the hopping vector direction. Finally, the mass-scaled one-dimensional diabatic forces in Eqs. 2 and 3 are converted from the mass-scaled multi-dimensional diabatic forces based on

$$\frac{\sqrt{|F_2F_1|}}{\sqrt{\mu}} = \sqrt{\left|\sum_{i=1}^{N}\frac{1}{m_i}\sum_{\alpha=x.y.z} F_2^{i\alpha}F_1^{i\alpha}\right|} \tag{4}$$

and

$$\frac{|F_2 - F_1|}{\sqrt{\mu}} = \sqrt{\left|\sum_{i=1}^{N}\frac{1}{m_i}\sum_{\alpha=x,y,z}\left(F_2^{i\alpha} - F_1^{i\alpha}\right)^2\right|} \tag{5}$$

in which N is the number of nuclei in the system, m_i is the i-th atomic mass, $F_1^{i\alpha}$ and $F_2^{i\alpha}$ are the multidimensional diabatic forces of the i-th atom (α : x, y, and z) related to the involved two electronic states. Notably, these multidimensional diabatic forces can be further converted from multi-dimensional adiabatic forces, which are directly calculated by external electronic structure packages (such as ChemShell and MNDO99). The details can be found in many recent works by Zhu and co-workers.[48,52] This Zhu-Nakamura non-adiabatic dynamics method has recently been implemented as a module into the generalized trajectory-based surface hopping (GTSH) package.[53,54] Compared with the original fewest-switches surface-hopping method developed by Tully et al.[44,45] the advantage of using the Zhu-Nakamura method is that it does not need to compute expensive non-adiabatic couplings.

Initial atomic coordinates and velocities in the present non-adiabatic dynamics simulations are sampled from 200 ps ground-state molecular-mechanics based MD simulations. A total of 10 surface-hopping trajectories are run starting from the S_1 state in the Franck-Condon region, with all the relevant energies and gradients being computed "on-the-fly". Within the simulation, when the energy gap between the involved S_1 and S_0 states is less than 10 kcal/mol, the improved Landau-Zener formula is then applied to compute the non-adiabatic transition probability. We use a time step of 1.0 fs for nuclear propagation and each trajectory is propagated during 1 ps. In order to save computational efforts, the QM method employed in the present QM/MM nonadiabatic dynamics simulations is the semiempirical OM2/MRCI method implemented in the MNDO99 code.[55– 58] The benchmarks show that the OM2/MRCI method is reasonably accurate compared with the high-level CASPT2//CASSCF method (Figure S15).

In the OM2/MRCI calculations, the restricted open-shell Hartree– Fock formalism is applied in the self-consistent field (SCF) treatment. The active space in the MRCI calculations includes 16 electrons in 14 orbitals. In terms of the SCF configuration, it comprises the seven highest doubly occupied orbitals, the two singly occupied orbitals, and the five lowest unoccupied orbitals. For the MRCI treatment, three configuration state functions are chosen as references, namely the SCF configuration and the two closed-shell configurations derived therefrom (i.e. all singlet configurations generated from HOMO and LUMO of the closed-shell ground state). The MRCI wave function is built by allowing all single and double excitations from these three references. More details can be found in recent work by Thiel and coworkers including some of us. [35,42,59]

The OM2/MRCI calculations with a (16,14) active space and the 1 ps QM/MM non-adiabatic dynamics trajectories are performed using high-performance computer resources provided by the supercomputing center of the Beijing PARATERA Tech Corp., Ltd.

1.4 Active Spaces of the QM region

1.4.1 Cph1

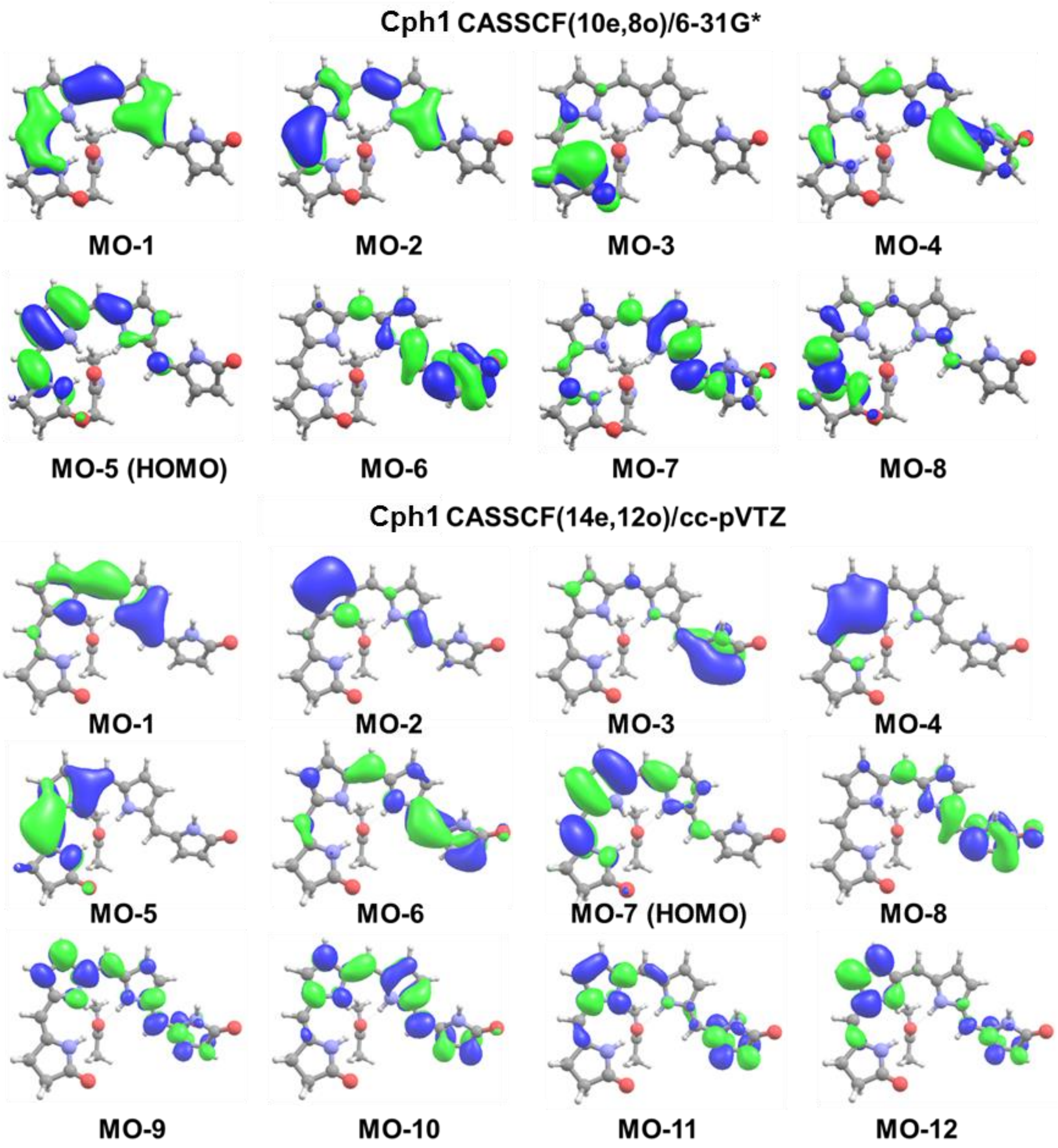

Figure S3. Active spaces used for the CASSCF (top) and CASPT2 (bottom) calculations of Cph1 (see text).

1.4.2 AnPixJ

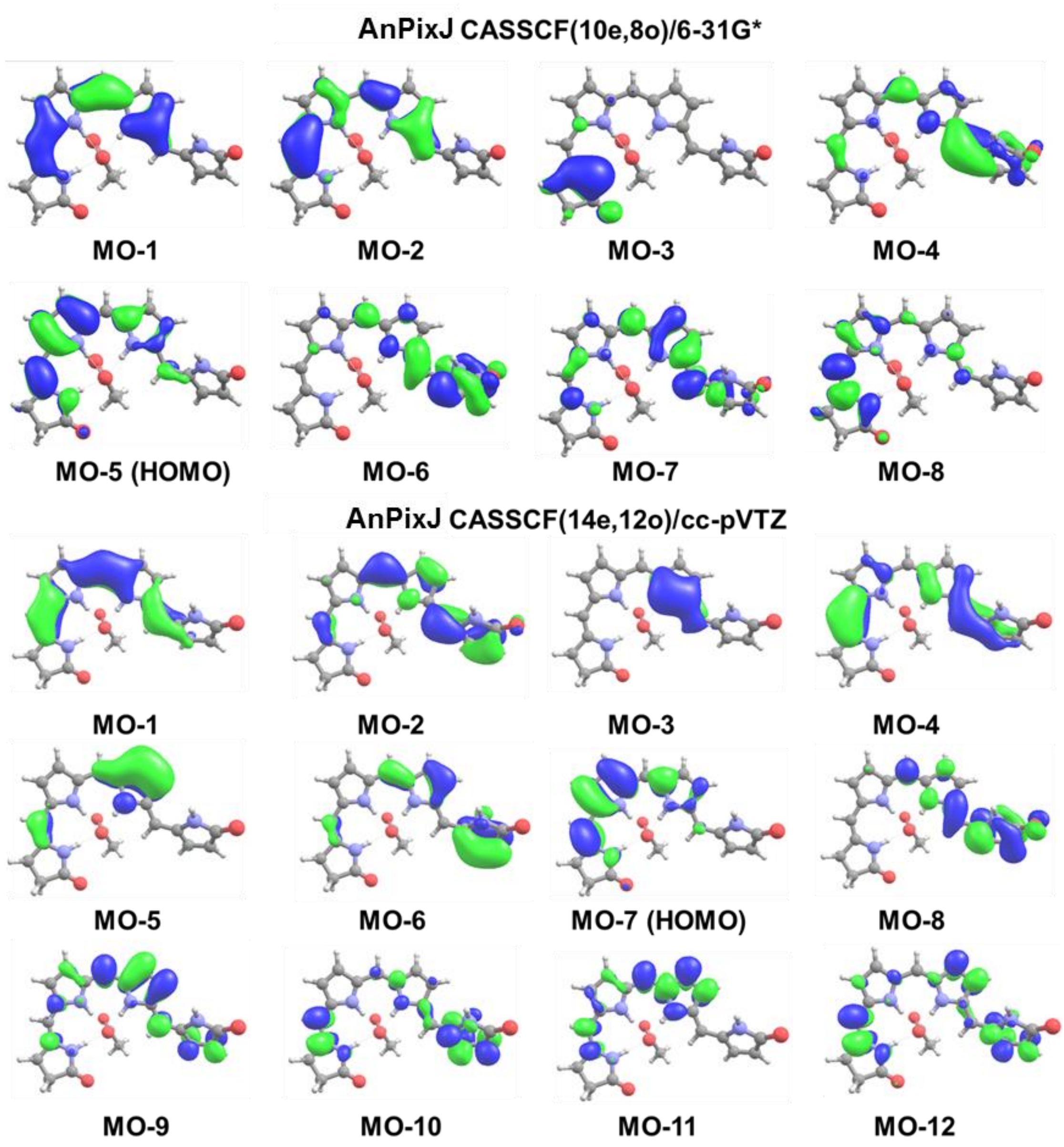

Figure S4. Active spaces used for the CASSCF (top) and CASPT2 (bottom) calculations of AnPixJ (see text).

2. Relevant Bond Lengths along Photoisomerization Path

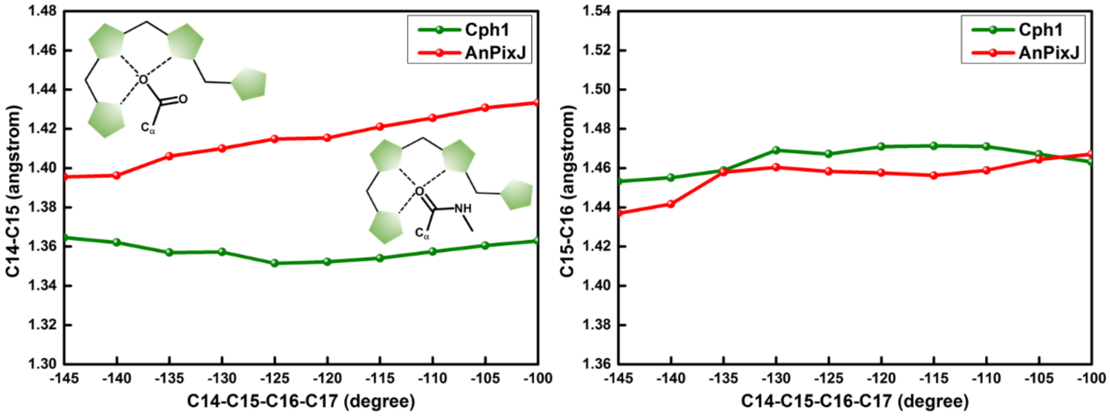

Figure S5. QM(CASSCF)/MM optimized bond lengths of C14-C15 (left) and C15-C16 (right) along the S_1 minimum-energy isomerization paths as functions of the dihedral angle for the internal rotation around the C15-C16 bond of PCB in Cph1 and AnPixJ.

3. <u>AnPixJ-COOH</u>

3.1 Optimized Minimum-Energy Structures

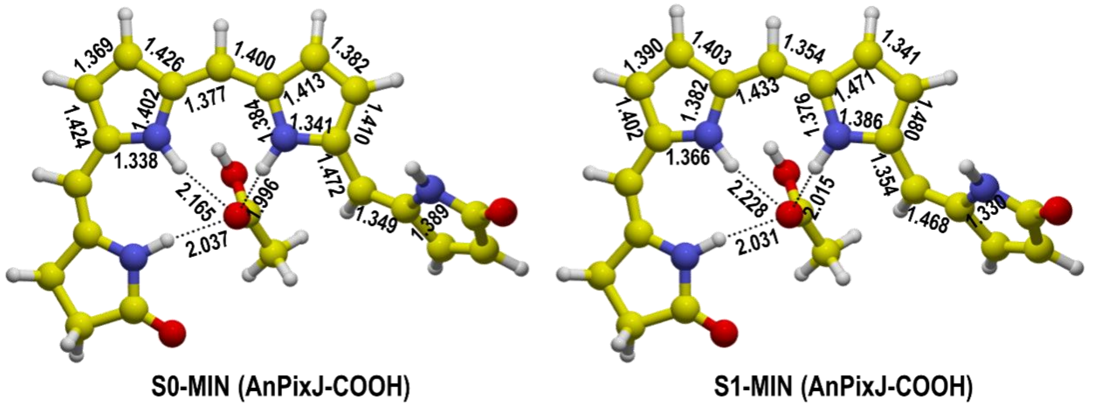

Figure S6. QM(CASSCF)/MM optimized S_0 and S_1 minimum-energy structures in an AnPixJ-COOH model containing a neutral carboxylic group, with selected bond lengths (in Angstroms).

3.2 Optimized Minimum-Energy Path

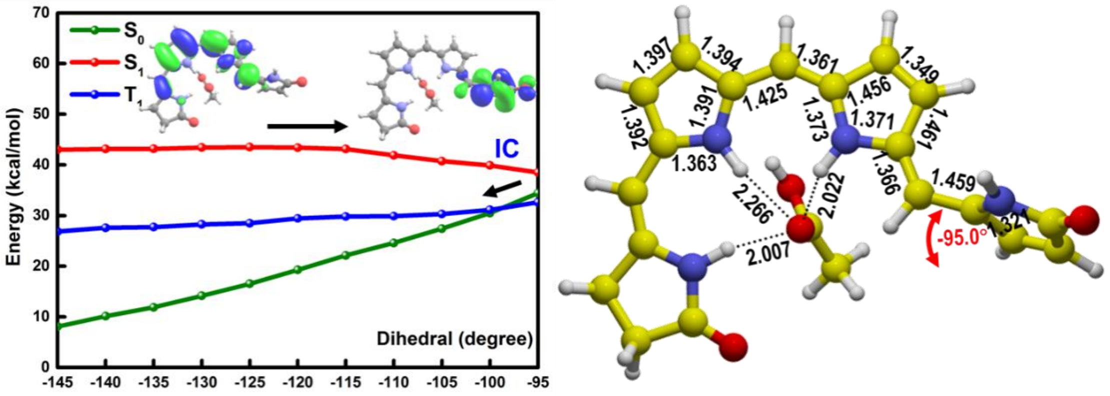

Figure S7. QM(CASPT2)/MM calculated S_1 minimum-energy isomerization path for the internal rotation around the C15-C16 bond of PCB in an AnPixJ-COOH model containing a neutral carboxylic group. Also shown are the geometric and electronic structures at a C14-C15-C16-C17 dihedral angle of ca. -95°.

4. <u>Gas Phase Calculations</u>

4.1 Gas Phase Optimized Minimum-Energy Structures

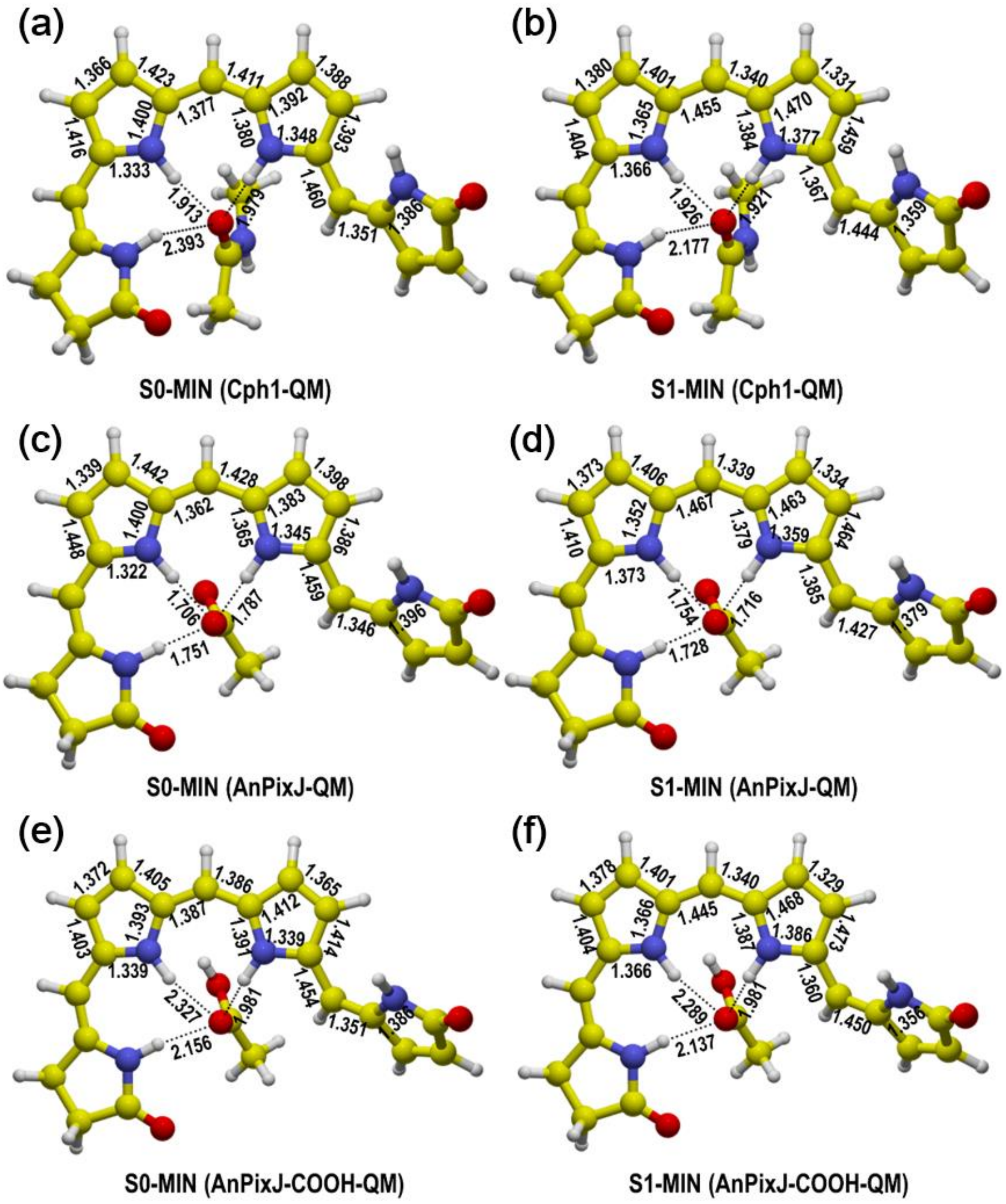

Figure S8. CASSCF optimized S_0 and S_1 minimum-energy structures of the gas-phase model of Cph1 (panel a and b), AnPixJ (panel c and d) and AnPixJ-COOH model (panel e and f) containing a neutral carboxylic group with selected bond lengths (in Angstroms).

4.2 Gas Phase Optimized Minimum-Energy Paths

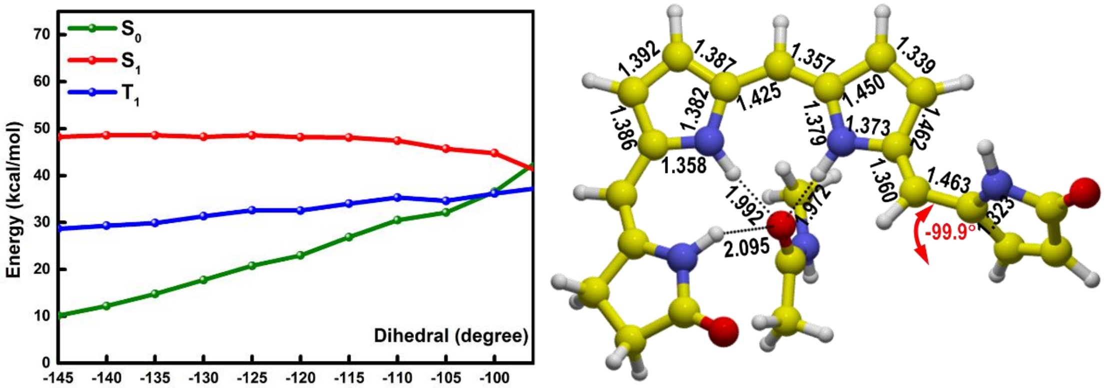

Figure S9. CASPT2 isomerization paths along the internal rotation around the C15-C16 bond of the gas-phase model of Cph1 (left) calculated along the optimized S_1 minimum-energy. Also shown are the geometric and electronic structures at a C14-C15-C16-C17 dihedral angle of ca. -100° (right).

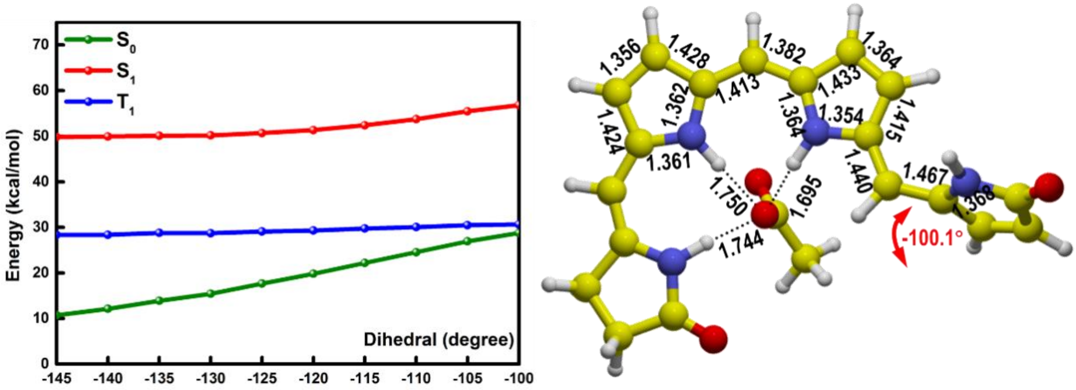

Figure S10. CASPT2 isomerization paths along the internal rotation around the C15-C16 bond of the gas-phase model of AnPixJ (left) calculated along the optimized S_1 minimum-energy. Also shown are the geometric and electronic structures at a C14-C15-C16-C17 dihedral angle of ca. -100° (right).

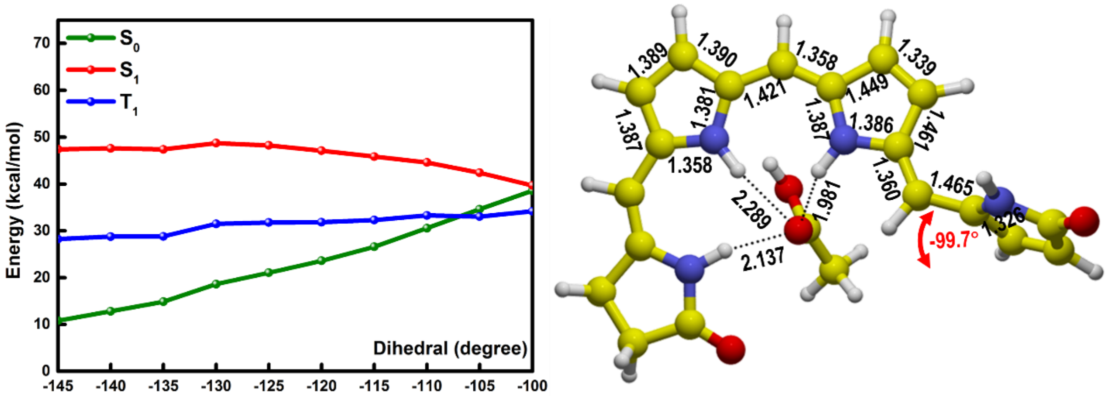

Figure S11. CASPT2 isomerization paths along the rotation of the C15-C16 bond of the gas-phase model of artificially constructed AnPixJ with a neutral carboxylic group (left) calculated along the optimized S_1 minimum-energy. Also shown are the geometric and electronic structures

at the C14-C15-C16-C17 dihedral angle of ca. -100° (right).

5. T_1 Minimum-Energy Structures

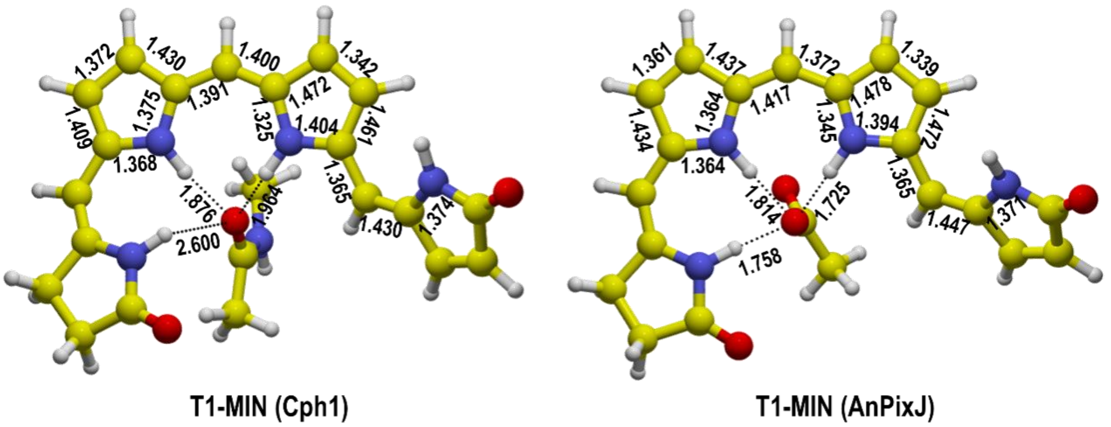

Figure S12. QM(CASSCF)/MM optimized T_1 minimum-energy structures of Cph1 and AnPixJ with selected bond lengths (in Angstrom).

6. Hydrogen-Bonding Networks

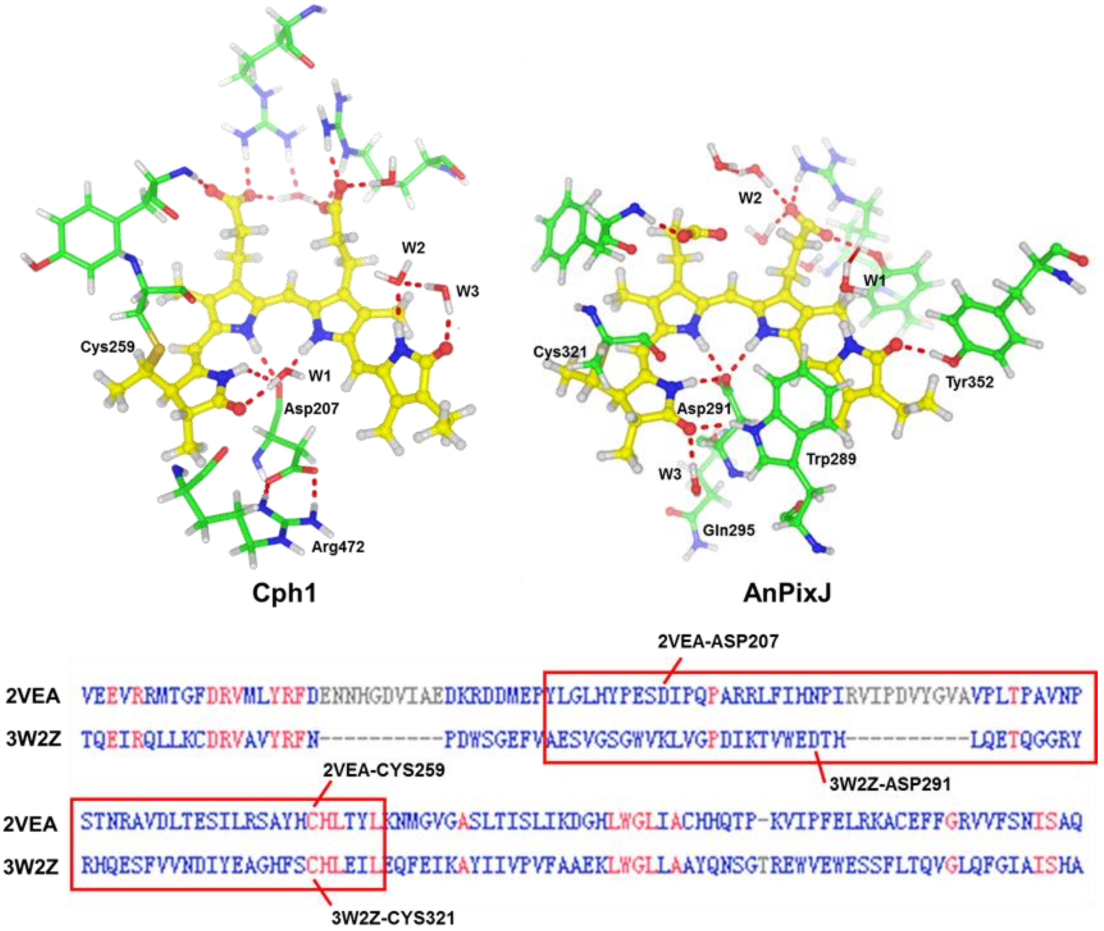

Figure S13. Schematic diagram of hydrogen-bonding networks near the PCB chromophore in Cph1(left; PDB code: 2VEA) and AnPixJ (right; PDB code: 3W2Z).

7. Approximated Valence Bond Patterns

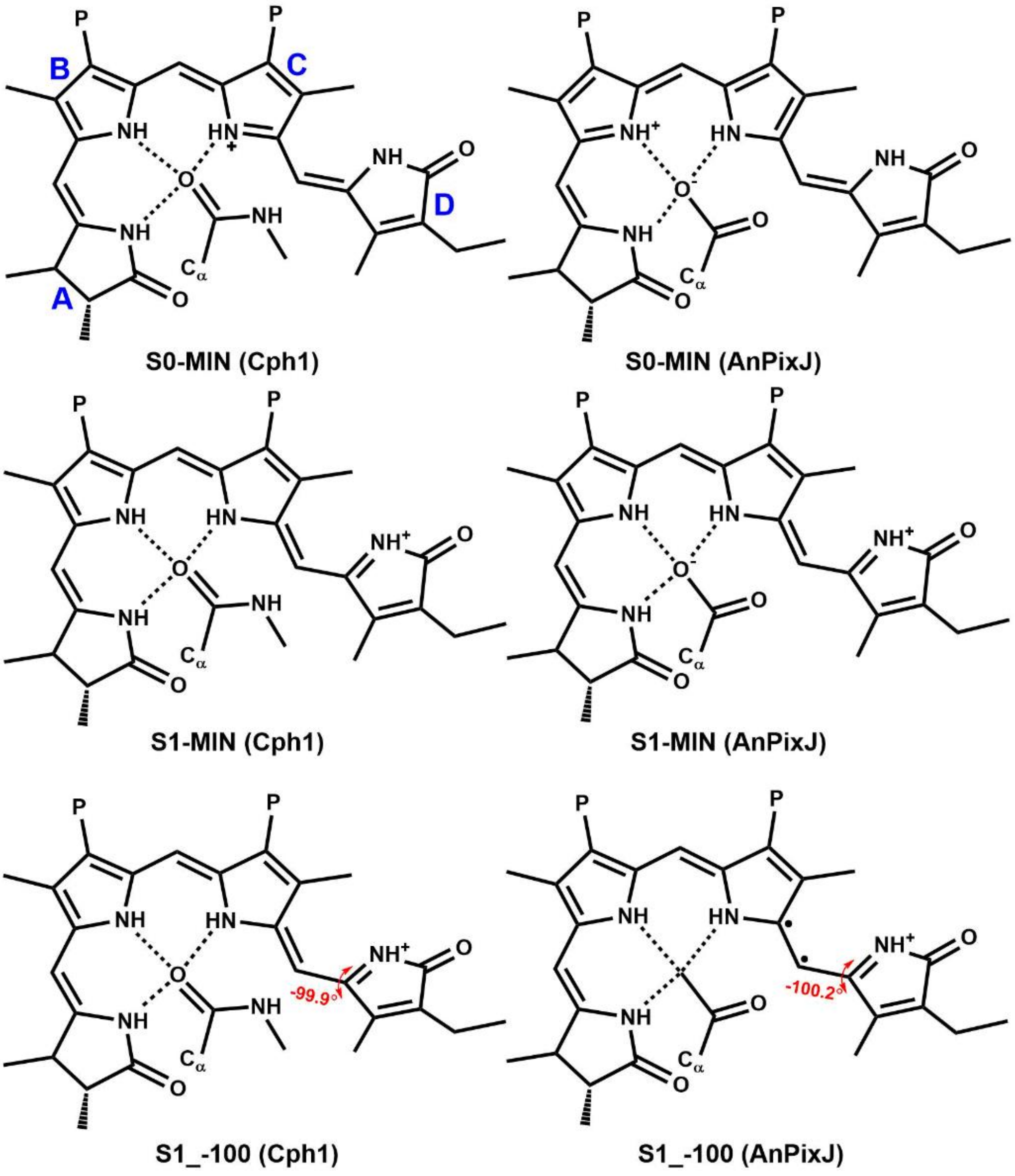

Figure S14. Approximated valence bond patterns of relevant QM regions of Cph1 and AnPixJ.

8. OM2/MRCI Optimized Minimum-Energy Paths

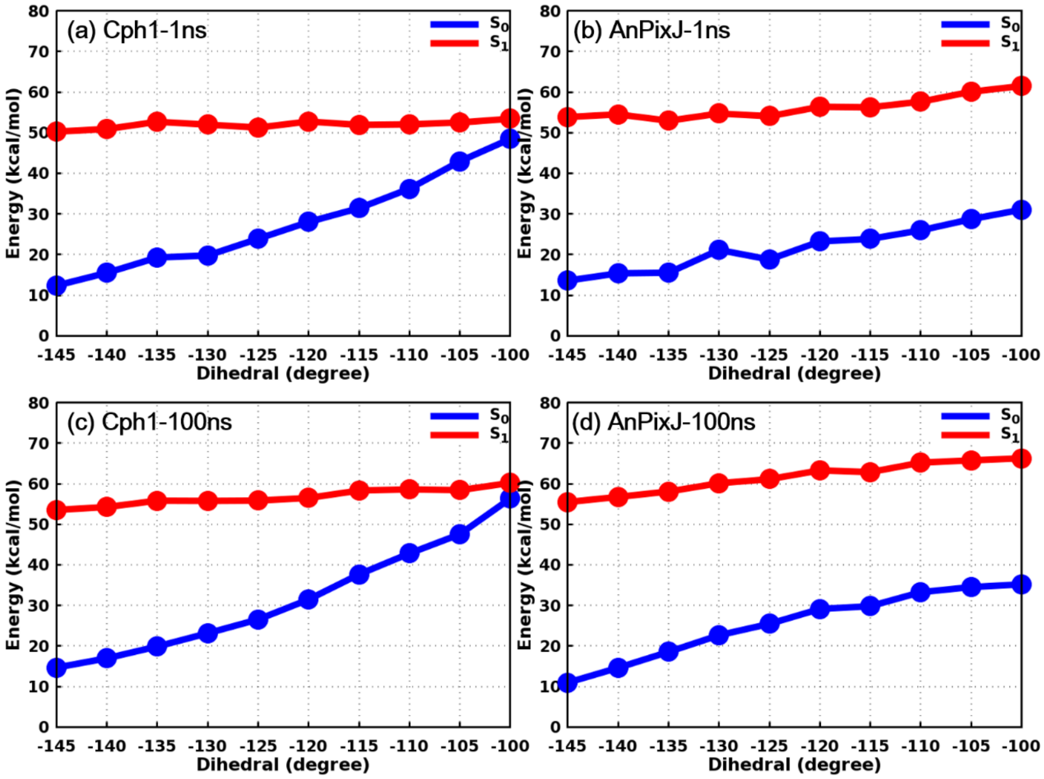

Figure S15. QM(OM2(16,14)/MRCI)/MM calculated S_1 minimum-energy isomerization paths along the C14-C15-C16-C17 dihedral angle of the PCB chromophore in Cph1 (a and c) and AnPixJ (b and d) based on 1 ns (a and b) and 100 ns (c and d) MD snapshots.

9. Nonadiabatic Dynamics Simulations

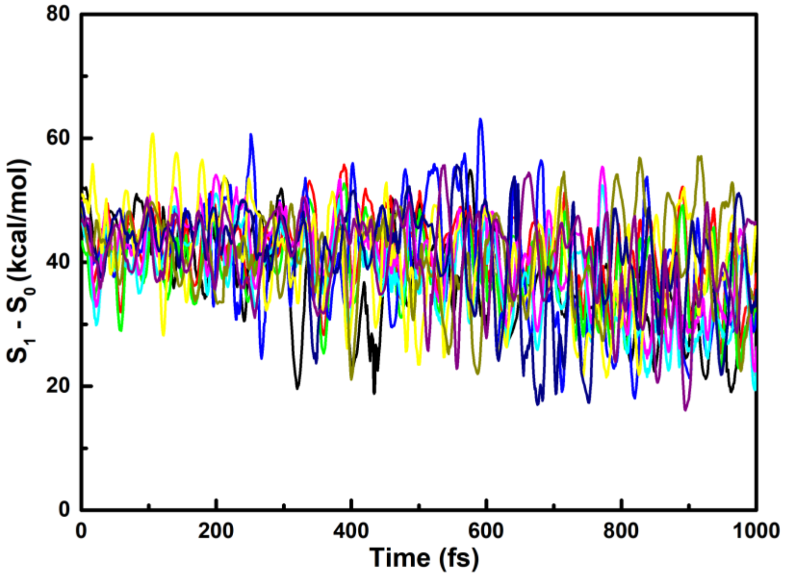

Figure S16. Time-dependent energy gap between the S_1 and S_0 states in the QM(OM2(16,14)/MRCI)/MM nonadiabatic dynamics simulations based on the Zhu-Nakamura scheme. It should be noted that none of these trajectories hops to ground state during the simulation time of 1 ps due to the large energy gap.

10. Tables

Table S1: Natural Charges on the Four Pyrrole Units of the PCB Chromophore of Cph1 in the Different Structures Calculated at the QM(CASSCF)/MM Level of Theory.

A	B	C	D	A	B	C	D
S0-MIN (S_0 state)				S1-MIN (S_1 state)			
0.1178	0.2868	0.4066	0.1611	0.2688	0.1100	0.4051	0.1813
S1 relaxed structure at -100° (S_0 state)				S1 relaxed structure at -100° (S_1 state)			
0.0825	0.02535	0.1727	0.6885	0.0910	0.1707	0.5359	0.1712

Table S2: Natural Charges on the Four Pyrrole Units of the PCB Chromophore of AnPixJ in the Different Structures Calculated at the QM(CASSCF)/MM Level of Theory.

A	B	C	D	A	B	C	D
S0-MIN (S_0 state)				S1-MIN (S_1 state)			
0.1663	0.5851	0.0803	0.0533	0.3221	0.2465	0.2725	0.0418
S_1 relaxed structure at -100° (S_0 state)				S_1 relaxed structure at -100°(S_1 state)			
0.1808	0.4622	0.2823	-0.0487	0.2067	0.4712	0.2507	-0.0520

Table S3: Vertical Excitation Energies and oscillator strengths of S_1 states (in kcal/mol) of Cph1 and AnPixJ Computed at the QM(CASPT2)/MM Level.

System	Calc. (kcal/mol)	Osc.	Exp. (kcal/mol)
Cph1	43.9	0.90	43.3
AnPixJ	47.6	0.71	44.1

Table S4: Wavefunction Relevant Information of Electronic Configuration of S_0 and S_1 Minima of the PCB Chromophore in Cph1 and AnPixJ Calculated at the QM(CASPT2)/MM Level.

<table>
<tr><th>Cph1</th><th>S_0 (weight)</th><th>S_1(weight)</th><th>T_1(weight)</th></tr>
<tr><td rowspan="2">S0-MIN</td><td colspan="3">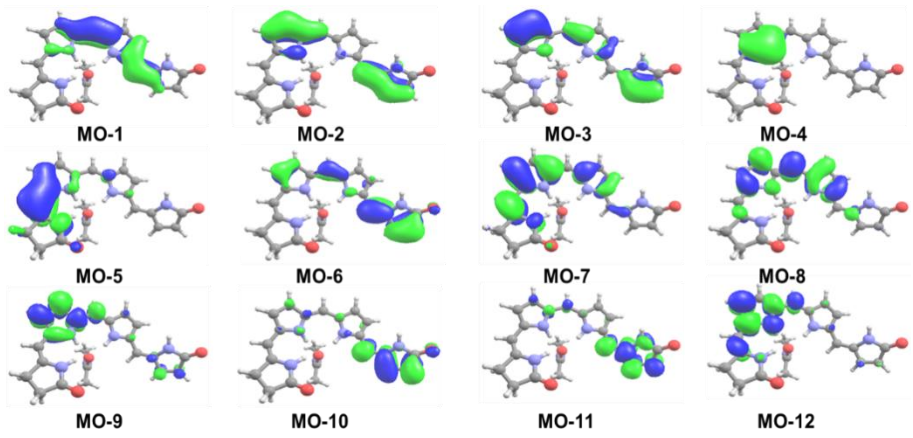
</td></tr>
<tr><td>222222200000 (0.82)</td><td>222222ud0000 (0.78)</td><td>/</td></tr>
<tr><td rowspan="2">S1-MIN</td><td colspan="3">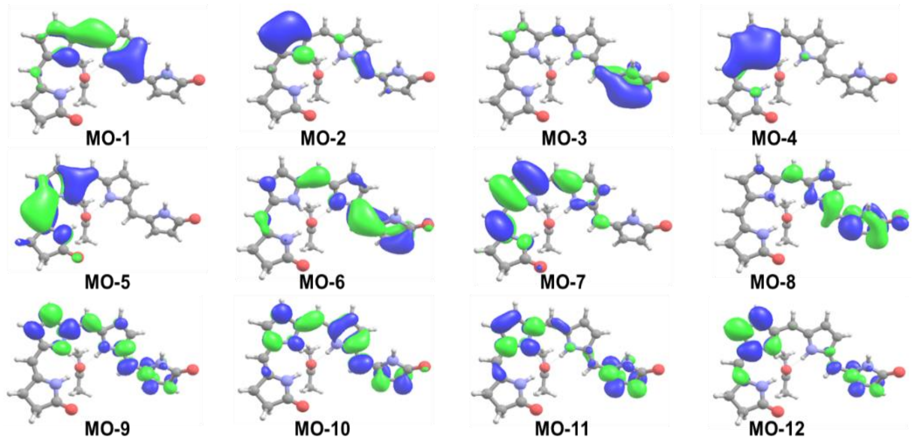
</td></tr>
<tr><td>222222200000 (0.51)
222222ud0000 (0.29)</td><td>222222200000 (0.25)
222222ud0000 (0.46)</td><td>222222uu0000 (0.79)</td></tr>
<tr><td rowspan="2">S1 -
100°</td><td colspan="3">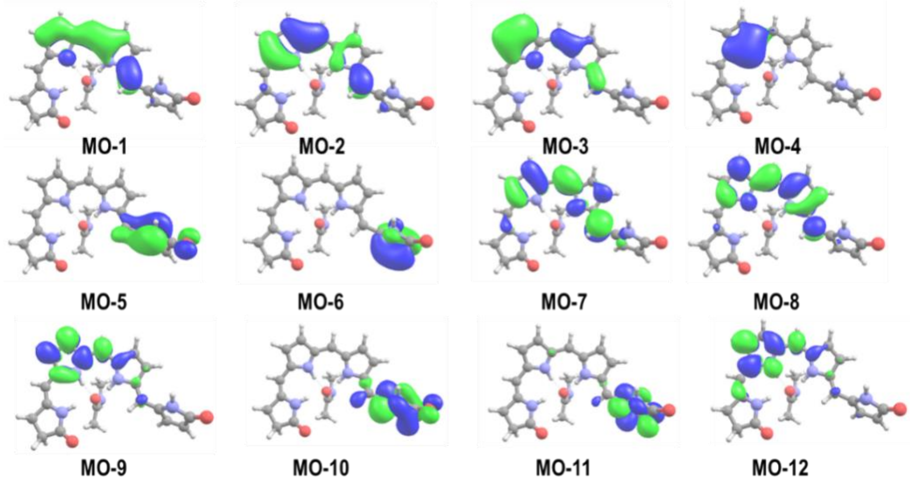
</td></tr>
<tr><td>222222200000 (0.66)
222222u00d00 (0.17)</td><td>222222u00d00 (0.64)
222222200000 (0.17)</td><td>222222u00u00 (0.80)</td></tr>
</table>

<table>
<tr><td>AnPixJ</td><td>S_0 (weight)</td><td>S_1(weight)</td><td>T_1(weight)</td></tr>
<tr><td rowspan="2">S0-MIN</td><td colspan="3">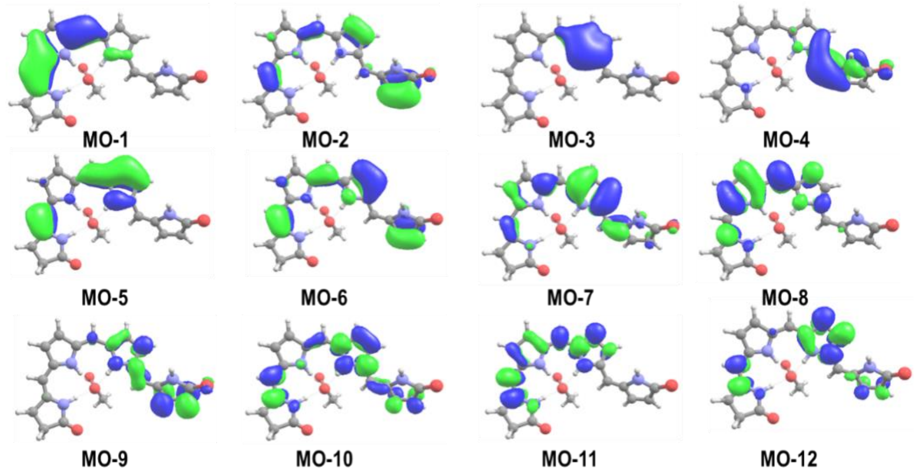
</td></tr>
<tr><td>222222200000 (0.82)</td><td>222222ud0000 (0.80)</td><td>/</td></tr>
<tr><td rowspan="2">S1-MIN</td><td colspan="3">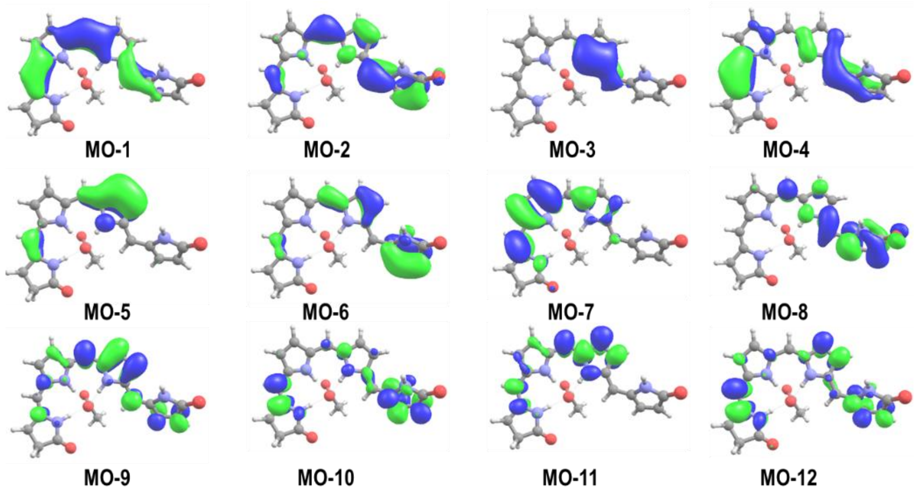
</td></tr>
<tr><td>222222200000 (0.51)
222222ud0000 (0.29)</td><td>222222200000 (0.26)
222222ud0000 (0.48)</td><td>222222uu0000 (0.75)</td></tr>
<tr><td rowspan="2">S1 -
100°</td><td colspan="3">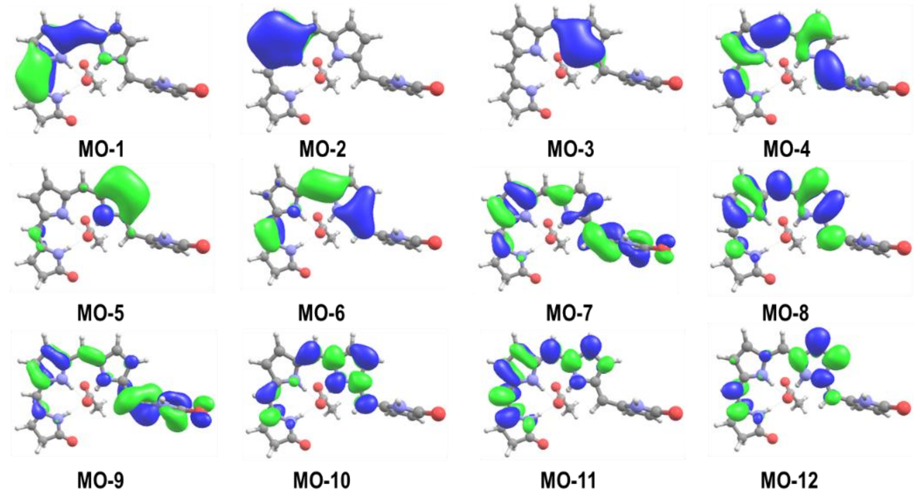
</td></tr>
<tr><td>222222200000 (0.45)</td><td>222222ud0000 (0.16)</td><td>222222u0u000 (0.31)</td></tr>
</table>

	222222002000 (0.31)	2222220ud000 (0.19)<br>222u2220d000 (0.24)<br>222u22d02000 (0.13)	

Evolution of the S_1 Electronic Structures along the Photoisomerization Path in AnPixJ

As discussed in the main text, in AnPixJ, the S_1 electronic structure at the C14-C15-C16-C17 dihedral angle of -100° is different from that at the S_1 minimum. The S_1 state is of main charge-transfer character (MO-7 to MO-8) at its minimum (weight: 0.48 in Table S4). However, more charge-transfer electronic configurations are involved in the S_1 state when the C14-C15-C16-C17 dihedral angle proceeds beyond -130° (Fig. 3b and Table S4). For example, at the S_1 structure with a C14-C15-C16-C17 dihedral angle of -100°, there are four comparable electronic configurations (weights: 0.16, 0.19, 0.24, and 0.13 in Table S4). In these configurations, there are molecular orbitals with clear antibonding character for the C14=C15 double bond, which makes it longer (Fig. S5). For these reasons, the corresponding S_1 energy along the photoisomerization path increases slightly compared with that in Cph1.

Table S5: Absolute Energies (A.E., Hartree), Relative Energies (ΔE, kcal/mol), and MM Energies (Hartree) of Optimized Structures for Z/E Photoisomerization of Cph1 (Reaction Coordinate: C14-C15-C16-C17 Dihedral Angle, Degree). The Total QM/MM Energy Profiles are Plotted in Figure 3 (top left).

(C14-C15-C16-C17)	CASSCF A.E.	CASPT2 A.E.	MM	CASPT2 ΔE
**S_1 (-145)**				
S_0	-1344.37407	-1349.46742	-20.72790	10.4
S_1	-1344.29907	-1349.41163		45.4
T_1	-1344.34187	-1349.44111		26.9
**S_1 (-140)**				
S_0	-1344.36613	-1349.46051	-20.72855	14.7
S_1	-1344.29508	-1349.40787		47.8
T_1	-1344.33824	-1349.43643		29.8
**S_1 (-135)**				

S_0	-1344.35815	-1349.45352	-20.72869	19.1
S_1	-1344.29163	1349.40467		49.8
T_1	-1344.33520	-1349.43190		32.6
**S_1 (-130)**				
S_0	-1344.35382	-1349.45101	-20.72664	22.5
S_1	-1344.29129	-1349.40549		49.9
T_1	-1344.33456	-1349.43106		33.2
**S_1 (-125)**				
S_0	-1344.35097	-1349.44133	-20.72569	26.8
S_1	-1344.28941	-1349.40144		50.3
T_1	-1344.33303	-1349.42746		35.5
**S_1 (-120)**				
S_0	-1344.34465	-1349.43525	-20.72606	30.6
S_1	-1344.29031	-1349.40171		51.6
T_1	-1344.33156	-1349.42531		36.8
**S_1 (-115)**				
S_0	-1344.33835	-1349.42907	-20.72558	34.5
S_1	-1344.29196	-1349.40265		51.0
T_1	-1344.32987	-1349.42316		38.2
**S_1 (-110)**				
S_0	-1344.33164	-1349.42216	-20.72525	38.8
S_1	-1344.29437	-1349.40389		50.2
T_1	-1344.32831	-1349.42090		39.6
**S_1 (-105)**				
S_0	-1344.32323	-1349.41329	-20.72555	44.4
S_1	-1344.29519	-1349.40395		50.2
T_1	-1344.32485	-1349.41729		41.8
**S_1 (-100)**				
S_0	-1344.31565	-1349.40384	-20.72457	50.3

S_1	-1344.29713	-1349.40578		49.0
T_1	-1344.32218	-1349.41420		43.8

Table S6: Absolute Energies (A.E., Hartree), Relative Energies (ΔE, kcal/mol), and MM Energies (Hartree) of Optimized Structures for Z/E Photoisomerization of AnPixJ (Reaction Coordinate: C14-C15-C16-C17 Dihedral Angle, Degree). The Total QM/MM Energy Profiles are Plotted in Figure 3 (top right).

(C14-C15-C16-C17)	CASSCF A.E.	CASPT2 A.E.	MM	CASPT2 ΔE
**S_1 (-145)**				
S_0	-1324.73712	-1329.71163	-14.38108	4.1
S_1	-1324.66558	-1329.65145		41.8
T_1	-1324.71085	-1329.68434		21.2
**S_1 (-140)**				
S_0	-1324.73401	-1329.70943	-14.3811	5.5
S_1	-1324.66472	-1329.65116		42.0
T_1	-1324.71117	-1329.68432		21.2
**S_1 (-135)**				
S_0	-1324.73645	-1329.70660	-14.38145	7.2
S_1	-1324.66379	-1329.65216		41.4
T_1	-1324.71091	-1329.68478		20.9
**S_1 (-130)**				
S_0	-1324.73308	-1329.70412	-14.38117	8.8
S_1	-1324.66337	-1329.65181		41.6
T_1	-1324.71121	-1329.68506		20.7
**S_1 (-125)**				
S_0	-1324.72881	-1329.70063	-14.38169	11.0
S_1	-1324.66226	-1329.65088		42.2
T_1	-1324.71032	-1329.68414		21.3
**S_1 (-120)**				
S_0	-1324.72803	-1329.69927	-14.38111	11.8
S_1	-1324.66675	-1329.65299		42.2

T_1	-1324.71184	-1329.68440		21.2
**S_1 (-115)**				
S_0	-1324.72271	-1329.69486		14.6
S_1	-1324.66429	-1329.65062	-14.38175	42.3
T_1	-1324.71017	-1329.68277		22.2
**S_1 (-110)**				
S_0	-1324.71827	-1329.69099		17.0
S_1	-1324.66231	-1329.64823	-14.38200	43.9
T_1	-1324.70249	-1329.67547		23.2
**S_1 (-105)**				
S_0	-1324.71395	-1329.68785		19.0
S_1	-1324.65976	-1329.64582	-14.38125	45.4
T_1	-1324.70789	-1329.68066		23.5
**S_1 (-100)**				
S_0	-1324.71025	-1329.68388		21.5
S_1	-1324.65809	-1329.64183	-14.38106	47.9
T_1	-1324.70581	-1329.67869		24.7

Table S7: Absolute Energies (A.E., Hartree), Relative Energies (ΔE, kcal/mol), and MM Energies (Hartree) of Optimized Structures for Z/E Photoisomerization of AnPixJ-COOH (Reaction Coordinate: C14-C15-C16-C17 Dihedral Angle, Degree). The Total QM/MM Energy Profiles are Plotted in Figure S7 (left).

**(C14-C15-C16-C17)**	**CASSCF A.E.**	**CASPT2 A.E.**	**MM**	**CASPT2 ΔE**
**S_1 (-145)**				
S_0	-1325.05289	-1329.99475		8.1
S_1	-1324.98518	-1329.93909	-14.37211	43.0
T_1	-1325.02446	-1329.96487		26.8
**S_1 (-140)**				
S_0	-1325.04770	-1329.99148	-14.37232	10.1

S_1	-1324.98457	-1329.93889		43.1
T_1	-1325.02263	-1329.96368		27.6
**S_1 (-135)**				
S_0	-1325.04408	-1329.98869		11.9
S_1	-1324.98455	-1329.93882	-14.37294	43.2
T_1	-1325.02237	-1329.96347		27.7
**S_1 (-130)**				
S_0	-1325.03974	-1329.98507		14.2
S_1	-1324.98448	-1329.93842	-14.37305	43.4
T_1	-1325.02184	-1329.96262		28.2
**S_1 (-125)**				
S_0	-1325.03520	-1329.98129		16.5
S_1	-1324.98516	-1329.93828	-14.37333	43.5
T_1	-1325.02178	-1329.96225		28.5
**S_1 (-120)**				
S_0	-1325.02968	-1329.97693		19.3
S_1	-1324.98596	-1329.93845	-14.37035	43.4
T_1	-1325.01968	-1329.96073		29.4
**S_1 (-115)**				
S_0	-1325.02469	-1329.97239		22.1
S_1	-1324.98774	-1329.93889	-14.37052	43.1
T_1	-1325.01941	-1329.96012		29.8
**S_1 (-110)**				
S_0	-1325.02035	-1329.96845		24.6
S_1	-1324.99079	-1329.94088	-14.36958	41.9
T_1	-1325.01961	-1329.96001		29.9
**S_1 (-105)**				
S_0	-1325.01522	-1329.96394	-14.36937	27.4
S_1	-1324.99352	-1329.94271		40.7

T_1	-1325.01889	-1329.95935		30.3
**S_1 (-100)**				
S_0	-1325.00988	-1329.95902		30.5
S_1	-1324.99657	-1329.94404	-14.36877	39.9
T_1	-1325.01803	-1329.95802		31.1
**S_1 (-95)**				
S_0	-1325.00357	-1329.95277		34.4
S_1	-1324.99937	-1329.94626	-14.36857	38.5
T_1	-1325.01550	-1329.95563		32.6

Table S8: Absolute Energies (A.E., Hartree) and Relative Energies (ΔE, kcal/mol) of Optimized Structures for Z/E Photoisomerization for QM Part of Cph1 in Gas Phase (Reaction Coordinate: C14-C15-C16-C17 Dihedral Angle, Degree). The Total Energy Profiles are Plotted in Figure S12 (left).

**(C14-C15-C16-C17)**	**CASSCF A.E.**	**CASPT2 A.E.**	**CASPT2 ΔE**
**S_1 (-145)**			
S_0	-1343.82549	-1347.85311	10.2
S_1	-1343.75437	-1347.79245	48.2
T_1	-1343.79214	-1347.82363	28.7
**S_1 (-140)**			
S_0	-1343.82124	-1347.84984	12.2
S_1	-1343.75327	-1347.79188	48.6
T_1	-1343.79092	-1347.82258	29.3
**S_1 (-135)**			
S_0	-1343.81582	-1347.84576	14.8
S_1	-1343.75293	-1347.79189	48.6
T_1	-1343.79024	-1347.82168	29.9
**S_1 (-130)**			
S_0	-1343.80976	-1347.84103	17.7
S_1	-1343.75398	-1347.79242	48.2
T_1	-1343.78949	-1347.81937	31.3
**S_1 (-125)**			
S_0	-1343.80433	-1347.83619	20.8
S_1	-1343.75408	-1347.79188	48.6
T_1	-1343.78848	-1347.81735	32.6
**S_1 (-120)**			
S_0	-1343.79928	-1347.83267	23.0
S_1	-1343.75405	-1347.79251	48.2
T_1	-1343.78753	-1347.81746	32.5

**S_1 (-115)**			
S_0	-1343.79286	-1347.82649	26.9
S_1	-1343.75543	-1347.79267	48.1
T_1	-1343.78601	-1347.81506	34.0
**S_1 (-110)**			
S_0	-1343.78641	-1347.82071	30.5
S_1	-1343.75713	-1347.79369	47.4
T_1	-1343.78422	-1347.81302	35.3
**S_1 (-105)**			
S_0	-1343.78214	-1347.81814	32.1
S_1	-1343.75929	-1347.79648	45.7
T_1	-1343.78404	-1347.81414	34.6
**S_1 (-100)**			
S_0	-1343.77639	-1347.81106	36.5
S_1	-1343.76264	-1347.79795	44.8
T_1	-1343.78274	-1347.81167	36.2
**S_1 (-95)**			
S_0	-1343.77143	-1347.79939	43.8
S_1	-1343.76668	-1347.80489	40.4
T_1	-1343.78105	-1347.80955	37.5

Table S9: Absolute Energies (A.E., Hartree) and Relative Energies (ΔE, kcal/mol) of Optimized Structures for Z/E Photoisomerization for QM Part of AnPixJ in Gas Phase (Reaction Coordinate: C14-C15-C16-C17 Dihedral Angle, Degree). The Total Energy Profiles are Plotted in Figure S13 (left).

**(C14-C15-C16-C17)**	**CASSCF A.E.**	**CASPT2 A.E.**	**CASPT2 ΔE**
**S_1 (-145)**			
S_0	-1324.20478	-1328.13520	10.7
S_1	-1324.12705	-1328.06932	49.8

T_1	-1324.17222	-1328.10535	28.3
**S_1 (-140)**			
S_0	-1324.20131	-1328.13320	12.1
S_1	-1324.12548	-1328.06890	49.9
T_1	-1324.17192	-1328.10534	28.3
**S_1 (-135)**			
S_0	-1324.19769	-1328.13053	13.9
S_1	-1324.12448	-1328.06852	50.0
T_1	-1324.17115	-1328.10465	28.8
**S_1 (-130)**			
S_0	-1324.19444	-1328.12805	15.4
S_1	-1324.12356	-1328.06842	50.2
T_1	-1324.17130	-1328.10475	28.7
**S_1 (-125)**			
S_0	-1324.19001	-1328.12433	17.7
S_1	-1324.12225	-1328.06777	50.7
T_1	-1324.17079	-1328.10418	29.0
**S_1 (-120)**			
S_0	-1324.18602	-1328.12070	19.8
S_1	-1324.12114	-1328.06695	51.3
T_1	-1324.17062	-1328.10381	29.3
**S_1 (-115)**			
S_0	-1324.18182	-1328.11660	22.2
S_1	-1324.11970	-1328.06555	52.4
T_1	-1324.17013	-1328.10312	29.7
**S_1 (-110)**			
S_0	-1324.17766	-1328.11245	24.5
S_1	-1324.11815	-1328.06386	53.7
T_1	-1324.16971	-1328.10265	30.0

**S_1 (-105)**			
S_0	-1324.17370	-1328.10815	26.9
S_1	-1324.11642	-1328.06156	55.5
T_1	-1324.16922	-1328.10197	30.5
**S_1 (-100)**			
S_0	-1324.17058	-1328.10497	28.7
S_1	-1324.11511	-1328.05972	56.8
T_1	-1324.16886	-1328.10169	30.6
**S_1 (-95)**			
S_0	-1324.16858	-1328.10298	29.9
S_1	-1324.11429	-1328.05833	57.8
T_1	-1324.16860	-1328.10158	30.7

Table S10: Absolute Energies (A.E., Hartree) and Relative Energies (ΔE, kcal/mol) of Optimized Structures for Z/E Photoisomerization for QM Part of AnPixJ-COOH in Gas Phase (Reaction Coordinate: C14-C15-C16-C17 Dihedral Angle, Degree). The Total Energy Profiles are Plotted in Figure S14 (left).

(C14-C15-C16-C17)	CASSCF<br>A.E.	CASPT2<br>A.E.	CASPT2<br>ΔE
**S_1 (-145)**			
S_0	-1324.60812	-1328.52280	10.8
S_1	-1324.53731	-1328.46448	47.4
T_1	-1324.58486	-1328.49505	28.2
**S_1 (-140)**			
S_0	-1324.60434	-1328.51961	12.8
S_1	-1324.53748	-1328.46418	47.6
T_1	-1324.58412	-1328.49422	28.8
**S_1 (-135)**			
S_0	-1324.60031	-1328.51635	14.9
S_1	-1324.53822	-1328.46453	47.4
T_1	-1324.58395	-1328.49414	28.8
**S_1 (-130)**			
S_0	-1324.59356	-1328.51037	18.6
S_1	-1324.53649	-1328.46239	48.7
T_1	-1324.58090	-1328.48989	31.5
**S_1 (-125)**			
S_0	-1324.58910	-1328.50650	21.0
S_1	-1324.53756	-1328.46314	48.3
T_1	-1324.58079	-1328.48942	31.8
**S_1 (-120)**			
S_0	-1324.58442	-1328.50243	23.6
S_1	-1324.53955	-1328.46498	47.1

T_1	-1324.58092	-1328.48932	31.8
**S_1 (-115)**			
S_0	-1324.57978	-1328.49769	26.6
S_1	-1324.54172	-1328.46697	45.8
T_1	-1324.58063	-1328.48858	32.3
**S_1 (-110)**			
S_0	-1324.57348	-1328.49135	30.5
S_1	-1324.54317	-1328.46896	44.6
T_1	-1324.57912	-1328.48702	33.3
**S_1 (-105)**			
S_0	-1324.56864	-1328.48488	34.6
S_1	-1324.54647	-1328.47247	42.4
T_1	-1324.57909	-1328.48740	33.0
**S_1 (-100)**			
S_0	-1324.56427	-1328.47857	38.6
S_1	-1324.54909	-1328.47681	39.7
T_1	-1324.57827	-1328.48558	34.2
**S_1 (-95)**			
S_0	-1324.55899	-1328.46845	44.9
S_1	-1324.55294	-1328.48137	36.8
T_1	-1324.57691	-1328.48354	35.5

11. References

[1] L.-O. Essen, J. Mailliet, and J. Hughes, The Structure of a Complete Phytochrome Sensory Module in the Pr Ground State. Proc. Natl. Acad. Sci. U. S. A., 105(38):14709-14714, 2008.

[2] R. Narikawa, T. Ishizuka, N. Muraki, T. Shiba, T. Kurisu, and M. Ikeuchi, Structures of Cyanobacteriochromes from Phototaxis Regulators AnPixJ and TePixJ Reveal General and Specific Photoconversion Mechanism. Proc. Natl. Acad. Sci. U. S. A., 110(3):918-923, 2013.

[3] M. A. Mroginski, D. von Stetten, F. V. Escobar, H. M. Strauss, S. Kaminski, P. Scheerer, M. Gunther, D. H. Murgida, P. Schmieder, C. Bongards, W. Gartner, J. Mailliet, J. Hughes, L. O. Essen, and P. Hildebrandt, Chromophore Structure of Cyanobacterial Phytochrome Cph1 in the Pr State: Reconciling Structural and Spectroscopic Data by QM/MM Calculations. Biophys. J., 96(10):4153-4163, 2009.

[4] F. V. Escobar, T. Utesch, R. Narikawa, M. Ikeuchi, M. A. Mroginski, W. Gärtner, and P. Hildebrandt, Photoconversion Mechanism of the Second GAF Domain of Cyanobacteriochrome AnPixJ and the Cofactor Structure of Its Green-Absorbing State. Biochemistry, 52(29):4871-4880, 2013.

[5] D. A. Case, J. T. Berryman, R. M. Betz, D. S. Cerutti, T. E. Cheatham, T. A. Darden, R. E. Duke, T. J. Giese, H. Gohlke, A. W. Goetz, N. Homeyer, S. Izadi, P. Janowski, J. Kaus, A. Kovalenko, T. S. Lee, S. LeGrand, P. Li, T. Luchko, R. Luo, B. Madej, K. M. Merz, G. Monard, P. Needham, H. Nguyen, H. T. Nguyen, I. Omelyan, A. Onufriev, D. R. Roe, A. Roitberg, R. Salomon-Ferrer, C. L. Simmerling, W. Smith, J. Swails, R. C. Walker, J. Wang, R. M. Wolf, X. Wu, D. M. York, and P. A. Kollman, AMBER 2015, University of California, San Francisco, 2015.

[6] H. C. Andersen, Molecular Dynamics Simulations at Constant Pressure and/or Temperature. J. Chem. Phys., 72(4):2384–2393, 1980.

[7] J. A. Maier, C. Martinez, K. Kasavajhala, L. Wickstrom, K. E. Hauser and C. Simmerling, ff14SB: Improving the Accuracy of Protein Side Chain and Backbone Parameters from ff99SB. J. Chem. Theory Comput., 11(8): 3696-3713, 2015.

[8] W. L. Jorgensen, J. Chandrasekhar, J. D. Madura, R. W. Impey, and M. L. Klein, Comparison of Simple Potential Functions for Simulating Liquid Water. J. Chem. Phys., 79(2):926–935,

1983.

[9] J. M. Wang, R. M. Wolf, J. W. Caldwell, P. A. Kollman, and D. A. Case, Development and Testing of a General Amber Force Field. J. Comput. Chem., 25(9):1157-1174, 2004.

[10] K. Andersson, P.-Å. Malmqvist, B. O. Roos, A. J. Sadlej, and K. Wolinski, Second-Order Perturbation Theory with a CASSCF Reference Function. J. Phys. Chem., 94(14):5483-5488, 1990.

[11] K. Andersson, P.-Å. Malmqvist, and B. O. Roos, Second-Order Perturbation Theory with a Complete Active Space Self-Consistent Field Reference Function. J. Chem. Phys., 96(2):1218-1226, 1992.

[12] R. Ditchfield, W. J. Hehre, and J. A. Pople, Self-Consistent Molecular-Orbital Methods. IX. An Extended Gaussian-Type Basis for Molecular-Orbital Studies of Organic Molecules. J. Chem. Phys., 54(2):724-728, 1971.

[13] M. M. Francl, W. J. Pietro, W. J. Hehre, J. S. Binkley, M. S. Gordon, D. J. DeFrees, and J. A. Pople, Self-Consistent Molecular Orbital Methods. XXIII. A Polarization-Type Basis Set for Second-Row Elements. J. Chem. Phys., 77(7):3654-3665, 1982.

[14] X.-P. Chang, Y.-J. Gao, W.-H. Fang, G. L. Cui and W. Thiel, QM/MM Study on the Photoreactions of Dark- and Light-Adapted States of a Blue-Light YtvA LOV Photoreceptor. Angew. Chem. Int. Ed., 56(32):9341-9345, 2017.

[15] B. G. Levine and T. J. Martínez. Isomerization through Conical Intersections. Annu. Rev. Phys. Chem., 58:613–634, 2007.

[16] L.-H. Liu, S. Yuan, W.-H. Fang, and Y. Zhang. Probing Highly Efficient Photoisomerization of a Bridged Azobenzene by a Combination of CASPT2//CASSCF Calculation with Semiclassical Dynamics Simulation. J. Phys. Chem. A, 115(35):10027–10034, 2011.

[17] J. Cao, L.-H. Liu, W.-H. Fang, Z.-Z. Xie, and Y. Zhang. Photo-Induced Isomerization of Ethylene-Bridged Azobenzene Explored by Ab Initio Based Non-Adiabatic Dynamics Simulation: A Comparative Investigation of the Isomerization in the Gas and Solution Phases. J. Chem. Phys., 138(13):134306-134315, 2013.

[18] S.-H. Xia, G. L. Cui, W.-H. Fang, and W. Thiel. How Photoisomerization Drives Peptide Folding and Unfolding: Insights from QM/MM and MM Dynamics Simulations. Angew. Chem.

Int. Ed., 55(6):2067–2072, 2016

[19] T. H. Dunning, Jr., Gaussian Basis Sets for Use in Correlated Molecular Calculations. I. The Atoms Boron through Neon and Hydrogen. J. Chem. Phys., 90(2): 1007-1023, 1989.

[20] F. Aquilante, R. Lindh, and T. B. Pedersen, Unbiased Auxiliary Basis Sets for Accurate Two-Electron Integral Approximations. J. Chem. Phys., 127(11):114107, 2007. S28

[21] N. Försberg and P.-Å. Malmqvist, Multiconfiguration Perturbation Theory with Imaginary Level Shift. Chem. Phys. Lett., 274(1-3):196-204, 1997.

[22] G. Ghigo, B. O. Roos, and P.-Å. Malmqvist, A Modified Definition of the Zeroth-Order Hamiltonian in Multiconfigurational Perturbation Theory (CASPT2). Chem. Phys. Lett., 396(1):142-149, 2004.

[23] J. P. Zobel, J. J. Nogueira, and L. González, The IPEA Dilemma in CASPT2. Chem. Sci., 8(2):1482-1499, 2017.

[24] U. C. Singh and P. A. Kollman. A Combined Ab Initio Quantum Mechanical and Molecular Mechanical Method for Carrying out Simulations on Complex Molecular Systems: Applications to the CH_3Cl^+ Cl^- Exchange Reaction and Gas Phase Protonation of Polyethers. J. Comput. Chem., 7(6):718–730, 1986.

[25] H. M. Senn and W. Thiel. QM/MM Methods for Biomolecular Systems. Angew. Chem. Int. Ed., 48(7):1198–1229, 2009.

[26] See www.chemshell.org for ChemShell3.5, a computational chemistry shell.

[27] P. Sherwood, A. H. de Vries, M. F. Guest, G. Schreckenbach, C. R. A. Catlow, S. A. French, A. A. Sokol, S. T. Bromley, W. Thiel, A. J. Turner, S. Billeter, F. Terstegen, S. Thiel, J. Kendrick, S. C. Rogers, J. Casci, M. Watson, F. King, E. Karlsen, M. Sjovoll, A. Fahmi, A. Schäfer and C. Lennartz, QUASI: A General Purpose Implementation of the QM/MM Approach and Its Application to Problems in Catalysis. THEOCHEM. J. Mol. Struct., 632(1-3):1-28, 2003.

[28] S. Metz, J. Kästner, A. A. Sokol, T. W. Keal, and P. Sherwood, ChemShell—a Modular Software Package for QM/MM Simulations. Wiley Interdiscip. Rev. Comput. Mol. Sci., 4:101-110, 2014.

[29] M. J. Frisch, G. W. Trucks, H. B. Schlegel, G. E. Scuseria, M. A. Robb, J. R. Cheeseman, G. Scalmani, V. Barone, B. Mennucci, G. A. Petersson, et al., Gaussian 03, Revision D.02, Gaussian, Inc., Wallingford CT, 2010

[30] G. Krlström, R. Lindh, P.-Å. Malmqvist, B. O. Roos, U. Ryde, V. Veryazov, P.-O. Widmark, M. Cossi, B. Schimmelpfennig, P. Neogrády, et al. MOLCAS: A Program Package for Computational Chemistry. Comput. Mater. Sci., 28(2):222-229, 2003.

[31] F. Aquilante, L. De Vico, N. Ferré, G. Ghigo, P.-Å. Malmqvist, P. Neogrády, T. B. Pedersen, M. Pitoňák, M. Reiher, B. O. Roos, et al. MOLCAS 7: The Next Generation. J. Comput. Chem., 31(1):224-247, 2010.

[32] W. Smith and T. R. Forester, DL_POLY_2.0: A General-Purpose Parallel Molecular Dynamics Simulation Package. J. Mol. Graphics, 14(3):136-141, 1996.

[33] M. Barbatti, A. J. A. Aquino, J. J. Szymczak, D. Nachtigallová, P. Hobza and H. Lischka, Relaxation Mechanisms of UV-Photoexcited DNA and RNA Nucleobases. Proc. Natl. Acad. Sci. U. S. A., 107: 21453–21458, 2010.

[34] Y. Lu, Z. G. Lan and W. Thiel, Hydrogen Bonding Regulates the Monomeric Nonradiative Decay of Adenine in DNA Strands. Angew. Chem. Int. Ed., 50: 6864–6867, 2011.

[35] G. L. Cui, Z. G. Lan and W. Thiel, Intramolecular Hydrogen Bonding Plays a Crucial Role in the Photophysics and Photo-chemistry of the GFP Chromophore. J. Am. Chem. Soc., 134: 1662–1672, 2012.

[36] R. Long, N. J. English and Prezhdo, O. V. Photo-Induced Charge Separation across the GrapheneTiO_2 Interface Is Faster Than Energy Losses: A Time-Domain ab Initio Analysis. J. Am. Chem. Soc., 134: 14238–14248, 2012.

[37] S. A. Fischer, C. T. Chapman and X. S. Li, Surface Hopping with Ehrenfest Excited Potential. J. Chem. Phys., 135: 144102, 2011.

[38] S. A. Fischer, D. B. Lingerfelt, J. W. May and X. S. Li, Non-Adiabatic Molecular Dynamics Investigation of Photoionization State Formation and Lifetime in $Mn2^+$-Doped ZnO Quantum Dots. Phys. Chem. Chem. Phys., 16: 17507–17514, 2014.

[39] T. Nelson, S. Fernandez-Alberti, A. E. Roitberg and S. Tretiak, Non-adiabatic Excited-State Molecular Dynamics: Modeling Photophysics in Organic Conjugated Materials. Acc. Chem. Res., 47: 1155–1164, 2014.

[40] M. Richter, P. Marquetand, J. González-Vázquez, I. Sola, L. González, SHARC: ab Initio Molecular Dynamics with Surface Hopping in the Adiabatic Representation Including

Arbitrary Couplings. J. Chem. Theory Comput., 7: 1253–1258, 2011.

[41] M. Richter, P. Marquetand, J. González-Vázquez, I. Sola and L. González, Femtosecond Intersystem Crossing in the DNA Nu-cleobase Cytosine. J. Phys. Chem. Lett., 3: 3090–3095, 2012.

[42] Y.-T. Wang, X.-Y. Liu, G. L. Cui, W.-H. Fang and W. Thiel, Photoisomerization of Arylazopyrazole Photoswitches: Stereospe-cific Excited-State Relaxation. Angew. Chem. Int. Ed., 55: 14009–14013, 2016.

[43] S.-H. Xia, G. L. Cui, W.-H. Fang and W. Thiel, How Photoisomerization Drives Peptide Folding and Unfolding: Insights from QM/MM and MM Dynamics Simulations. Angew. Chem. Int. Ed., 55: 2067–2072, 2016.

[44] S. Hammes-Schiffer, J. C. Tully, Proton Transfer in Solution: Molecular Dynamics with Quantum Transitions. J. Chem. Phys., 101: 4657–4667, 1994.

[45] J. C. Tully and R. K. Preston, Trajectory Surface Hopping Approach to Non-adiabatic Molecular Collisions: Reaction of H^+ with D_2. J. Chem. Phys., 55: 562–572, 1971.

[46] L. Shen, B.-B. Xie, L. Liu, G. L. Cui and W.-H. Fang, Role of Multistate Intersections in Photochemistry. J. Phys. Chem. Lett., 11: 8490–8501, 2020.

[47] T. J. Martínez, Insights for Light-Driven Molecular Devices from Ab Initio Multiple Spawning Excited-State Dynamics of Organic and Biological Chromophores. Acc. Chem. Res., 39: 119–126, 2006.

[48] L. Yu, C. Xu, Y. Lei, C. Zhu and Z. Wen, Trajectory-Based Non-adiabatic Molecular Dynamics without Calculating Non-adiabatic Coupling in the Avoided Crossing Case: Trans <-> Cis Photoisomerization in Azobenzene. Phys. Chem. Chem. Phys., 16: 25883–25895, 2014.

[49] C. Zhu and H. Nakamura, Theory of Non-adiabatic Transition for General Two-State Curve Crossing Problems. I. Non-adiabatic Tunneling Case. J. Chem. Phys., 101: 10630–10647, 1994.

[50] C. Zhu and H. Nakamura, Theory of Non-adiabatic Transition for General Two-State Curve Crossing Problems. II. Landau-Zener Case. J. Chem. Phys., 102: 7448–7461, 1995.

[51] C. Zhu, K. Nobusada and H. Nakamura, New Implementation of the Trajectory Surface Hopping Method with Use of the Zhu-Nakamura Theory. J. Chem. Phys., 115: 3031–3044, 2001.

[52] L. Yu, C. Xu and C. Zhu, Probing the π -> π Photoisomerization Mechanism of Cis-Azobenzene by Multi-State Ab Initio on-the-Fly Trajectory Dynamics Simulation. Phys. Chem. Chem. Phys., 17: 17646–17660, 2015.

[53] G. L. Cui and W. Thiel, Generalized Trajectory Surface-Hopping Method for Internal Conversion and Intersystem Crossing. J. Chem. Phys., 141: 124101, 2014.

[54] X.-Y. Liu, Y.-G. Fang, B.-B. Xie, W.-H. Fang and G. L. Cui, QM/MM Non-adiabatic Dynamics Simulations on Photoinduced Wolff Rearrangements of 1,2,3-Thiadiazole. J. Chem. Phys., 146: 224302, 2017.

[55] W. Weber, Ph.D. Thesis, University of Zürich, 1996.

[56] W. Weber and W. Thiel, Orthogonalization Corrections for Semiempirical Methods. Theor. Chem. Acc., 103: 495-506, 2000.

[57] A. Koslowski, M. E. Beck and W. Thiel, Implementation of a General Multireference Configuration Interaction Procedure with Analytic Gradients in a Semiempirical Context Using the Graphical Unitary Group Approach. J. Comput. Chem., 24: 714-726, 2003.

[58] W. Thiel, MNDO99 program, version 6.1; Max-Planck-Institut für Kohlenforschung: Mülheim, Germany, 2007.

[59] G. L. Cui and W. Thiel, Photoinduced Ultrafast Wolff Rearrangement: A Non-adiabatic Dynamics Perspective. Angew. Chem. Int. Ed., 125: 451–454, 2013.

12. Cartesian Coordinates of All Optimized Structures (QM Part)

In xyz format (unit: Ångstroms)

CASSCF method

54

Cph1_S0_MIN

C	36.4814630791	57.7991917748	59.3555757570
H	36.2119761795	58.8129739719	59.6133910121
C	37.4755051649	57.3789740610	60.4325128587
O	37.4742290176	57.8780870329	61.5585878287
N	38.3864774674	56.4318509477	60.1295978687
H	38.3728945338	56.0498439654	59.2071190482
C	39.5391311531	56.1579320559	60.9397122066
H	39.5560626655	56.8458814081	61.7675398871
C	38.9895187018	58.2953716252	65.2904299977
N	36.9107618883	57.2653374946	64.3858206692
C	37.9300796041	57.4295812891	65.3198592749
C	37.6961750903	56.4203793663	66.3198795579
C	36.6301957595	55.6705310772	65.9278973490
C	36.1341939133	56.2557591727	64.7205170814
C	35.0349351595	55.8448211408	63.8587685073
N	33.3087250769	55.2668390368	65.5246200138
C	33.8405393550	55.3447068016	64.2485036183
C	32.8360772715	54.7099598544	63.3548494031
C	31.8369014875	54.2498397086	64.0951928907
C	32.1006097525	54.6124687730	65.5178471416
O	31.4208113439	54.4055265682	66.4771687666
N	36.6705796242	61.0912824314	61.3486851964
C	35.6056371044	61.3842924451	60.5502742853
C	36.0565776417	62.3688152716	59.4938492257
C	37.4909486977	62.7665020777	59.9319418748
C	37.8269497851	61.7796184036	61.0448353999
O	34.5167711729	60.9094313205	60.7161767197
C	39.0281964484	61.6698613807	61.6435016619
N	38.7798914270	59.8024314313	63.2827267473
C	39.4068967363	60.9104087338	62.8271397265
C	40.4591672725	61.1927980193	63.6890470559
C	40.4602255762	60.2093472368	64.6805185017
C	39.4046170810	59.3447623926	64.4303028389
H	39.7945691679	62.3204863987	61.2670979857
H	35.2298737246	55.9274861745	62.8067074120
H	39.6556093109	58.1675065985	66.1218259494
H	38.2404084811	59.2140533113	62.6702152816
H	36.5779581322	60.3701666295	62.0297885076

H	33.7025776258	55.6210295262	66.3814754268
H	36.8113265599	57.7581938066	63.5173717224
H	38.2146022348	62.6370881009	59.1373969895
H	35.3574158881	63.1954376359	59.4835411297
H	38.2566187044	56.3370154280	67.2510188925
H	36.3012258388	54.7241740828	66.3571458573
H	32.9989490654	54.6101308669	62.2817352417
H	30.9472337787	53.6900336506	63.8067910934
H	35.9853237172	61.9028769099	58.5110514984
H	37.5709474492	63.7869657502	60.3065365147
H	41.1636917638	62.0193651145	63.5968563310
H	41.1682983585	60.1592049936	65.5076737119
H	36.9029911669	57.8101168661	58.3504579703
H	35.5619330998	57.2268585692	59.4779128735
H	39.5794796200	55.1497222931	61.3519647105
H	40.4114997446	56.3267076164	60.3084001585
54			
Cph1_S1_MIN			
C	36.4856312439	57.8029334739	59.3571103061
H	36.2020318463	58.8151425908	59.6089621257
C	37.4999067583	57.4010440294	60.4205649890
O	37.5435957442	57.9645570387	61.5144270100
N	38.3787563159	56.4181689724	60.1465985811
H	38.3122817308	55.9637402871	59.2612782103
C	39.5370201921	56.1408569725	60.9468285491
H	39.5592869297	56.8204058315	61.7811475207
C	39.0952408132	58.2028253859	65.2619478854
N	37.0547587320	57.2458493415	64.2863006016
C	38.0331848916	57.3730211709	65.2337084097
C	37.7019084567	56.3724237417	66.2792571574
C	36.6114205355	55.7015266657	65.9012190612
C	36.1012665176	56.3144954351	64.6539364584
C	34.9466584585	56.1757358862	63.9400258966
N	33.2392646018	55.3343609416	65.5434566319
C	33.7497377027	55.4296429622	64.2899259341
C	32.8641164783	54.7040692634	63.4020730524
C	31.8497987664	54.2071053940	64.1289436661
C	32.0518160600	54.6215069063	65.5319957875
O	31.3642351627	54.4312464395	66.4911456974
N	36.6737662091	61.0713307278	61.3202970532
C	35.6004762983	61.3926596572	60.5350959841
C	36.0523635474	62.3833950912	59.4918933081
C	37.4877311436	62.7742792324	59.9326180598
C	37.8222582204	61.7665120962	61.0357019928

O	34.5075125816	60.9304732535	60.7004234999
C	39.0279287087	61.6672266439	61.6623506863
N	38.8411543107	59.7554076031	63.2793445323
C	39.4298322028	60.8949309891	62.8096532499
C	40.4846965035	61.1946375049	63.6883090492
C	40.5055862253	60.2226214843	64.6775901388
C	39.4655658279	59.3153287589	64.4144774198
H	39.7829513204	62.3475529192	61.3049215536
H	34.9261312611	56.6526977922	62.9757181467
H	39.7445059215	58.0815769644	66.1030425497
H	38.2545089318	59.1804290922	62.7016249267
H	36.5806460240	60.3251972322	61.9766772421
H	33.6105601523	55.7330779951	66.3929844008
H	37.0593235594	57.6468018752	63.3763463747
H	38.2044768591	62.6599436951	59.1269388668
H	35.3546566898	63.2107426069	59.4822838068
H	38.2512282200	56.2874408865	67.2168566259
H	36.2484284499	54.7759422461	66.3479821744
H	33.0638635333	54.5758221473	62.3382493628
H	30.9828780634	53.6172696070	63.8312705390
H	35.9839761752	61.9191962145	58.5080693291
H	37.5675694848	63.7951658198	60.3060929135
H	41.1727266381	62.0358990785	63.6048791388
H	41.2044099964	60.1757575383	65.5127644673
H	36.9040200074	57.8166242133	58.3507155240
H	35.5739833868	57.2186406116	59.4818784394
H	39.5823941907	55.1304233222	61.3530699675
H	40.4081895334	56.3112166529	60.3142871596

49

AnPixJ_S0_MIN

C	25.2883319110	51.4825033872	30.2874307974
H	25.5937664872	51.1075628357	31.2547787747
H	25.2490582684	50.6751214453	29.5691541168
C	26.2276567869	52.5763086079	29.8036974912
O	27.0129500620	53.0807496820	30.6781271699
O	26.1899148537	52.9397454606	28.6437275994
C	30.0674777240	54.1168419954	28.3475946812
N	29.6060049427	52.3108426413	30.0363440020
C	30.2496840441	52.8192770454	28.9433001648
C	31.1470613372	51.8611940629	28.5094728567
C	31.0278344265	50.7512173352	29.3700910664
C	30.0411224280	51.0567446039	30.2864293511
C	29.2745211909	50.1777725139	31.1807459806
N	31.0922449979	48.9503406237	32.2772262555

C	29.7548462517	49.1789757134	31.9449685805
C	29.0227972580	48.0180698792	32.4863088247
C	29.8919909985	47.1971612788	33.0699679050
C	31.2384277313	47.8316743628	33.0352241989
O	32.2672612710	47.4818480339	33.5596554946
N	25.9137550720	55.3588326827	31.7730746390
C	25.0516089805	55.1500673139	32.8256924058
C	24.2922959985	56.4294932443	33.0629033759
C	24.6435858515	57.3175291849	31.8392846539
C	25.7727821099	56.5510582620	31.1479403540
O	24.9942338903	54.1095012948	33.4130124209
C	26.4561597001	56.9978640091	30.0583570606
N	28.1827217221	55.2984814975	29.4695179901
C	27.5887758178	56.4776341775	29.3584398989
C	28.3208482318	57.2371690181	28.3404828291
C	29.3007057301	56.4555732121	27.8631795360
C	29.2462344974	55.1784986048	28.5613886556
H	26.1386848758	57.9538332134	29.7040403528
H	28.2082618018	50.2753559626	31.0900931011
H	30.7721190514	54.2803549718	27.5601434400
H	27.7539290900	54.4809942721	29.9063888241
H	26.4399666900	54.5634513891	31.4360252387
H	31.8208134411	49.6354352017	32.2182412303
H	28.7584306244	52.6746040549	30.4387902189
H	23.8157843392	57.3370757684	31.1351225112
H	24.6707106559	56.8345047410	33.9968591206
H	24.2632055015	51.8367696205	30.3954971287
H	31.7003317226	51.8597689192	27.5703472522
H	31.5648440915	49.8057916459	29.2935539047
H	27.9574963872	47.8573200249	32.3208984545
H	29.7392454884	46.2081136141	33.5018481865
H	23.2273898236	56.2623543182	33.2244783414
H	24.8692105233	58.3452674369	32.1237396493
H	28.0705078088	58.2480754769	28.0188232954
H	30.0275518983	56.7204125819	27.0953111831
49			
AnPixJ_S1_MIN			
C	25.2712514545	51.4734302995	30.2884673927
H	25.5790752447	51.1102752620	31.2591233208
H	25.2297574367	50.6582533484	29.5801095030
C	26.2146695207	52.5560662910	29.7951975850
O	26.9960993462	53.0612530375	30.6726582789
O	26.1862031997	52.9097576798	28.6323076402
C	30.0932726667	54.0550402717	28.3199406988

N	29.5393884187	52.3503194014	30.0087698948
C	30.2011872615	52.8496447906	28.9073140527
C	31.1331937190	51.7934892098	28.5057866987
C	31.0202943758	50.7662833137	29.3676453744
C	29.9560711281	51.0951246521	30.3204269283
C	29.2816802225	50.3353969418	31.2757133615
N	31.0761539609	48.9555749503	32.2737952218
C	29.7602638406	49.1507752030	31.9315181491
C	29.0407758473	47.9983094219	32.4172222157
C	29.9105712832	47.1728914082	33.0320469238
C	31.2366896754	47.8189851926	33.0289395775
O	32.2714902980	47.5065058028	33.5640015492
N	25.8957690473	55.3432909221	31.7613208355
C	25.0424467864	55.1393499502	32.8175127958
C	24.2883904231	56.4191137129	33.0641201045
C	24.6393357604	57.3118135380	31.8445450728
C	25.7674207475	56.5470320874	31.1510775741
O	24.9879458535	54.0965204415	33.4064258520
C	26.4739456675	57.0101595386	30.0723308550
N	28.2146583555	55.3038620833	29.4365041359
C	27.5921464656	56.5240974182	29.3354068456
C	28.3067869689	57.2537667602	28.3570334096
C	29.3305870454	56.4575124318	27.8858519614
C	29.2454112119	55.2212129776	28.5624667695
H	26.1585849498	57.9779967089	29.7393753918
H	28.3191239536	50.6986658913	31.5774731610
H	30.7835715462	54.2179737347	27.5223568776
H	27.7886956905	54.4918247019	29.8677038291
H	26.4251461019	54.5496748572	31.4212430854
H	31.7822848708	49.6668740486	32.2675073703
H	28.6597277410	52.6995271250	30.3561593383
H	23.8058015465	57.3379086847	31.1467781233
H	24.6729431946	56.8162342308	33.9989960122
H	24.2476280053	51.8317713465	30.3973424572
H	31.6954921958	51.7973046903	27.5720453445
H	31.5450370459	49.8126774141	29.3097837297
H	27.9768737268	47.8349168301	32.2455184289
H	29.7558905322	46.1906915249	33.4786164100
H	23.2232790017	56.2537189464	33.2261364081
H	24.8636787475	58.3390027417	32.1319813532
H	28.0549969445	58.2589208597	28.0189001940
H	30.0481195516	56.7257956498	27.1104564050

Cph1_-145_-100_S1_SCAN_C14-C15-C16-C17

54
-145

C	36.4856312439	57.8029334739	59.3571103061
H	36.2020318463	58.8151425908	59.6089621257
C	37.4999067583	57.4010440294	60.4205649890
O	37.5435957442	57.9645570387	61.5144270100
N	38.3787563159	56.4181689724	60.1465985811
H	38.3122817308	55.9637402871	59.2612782103
C	39.5370201921	56.1408569725	60.9468285491
H	39.5592869297	56.8204058315	61.7811475207
C	39.0952408132	58.2028253859	65.2619478854
N	37.0547587320	57.2458493415	64.2863006016
C	38.0331848916	57.3730211709	65.2337084097
C	37.7019084567	56.3724237417	66.2792571574
C	36.6114205355	55.7015266657	65.9012190612
C	36.1012665176	56.3144954351	64.6539364584
C	34.9466584585	56.1757358862	63.9400258966
N	33.2392646018	55.3343609416	65.5434566319
C	33.7497377027	55.4296429622	64.2899259341
C	32.8641164783	54.7040692634	63.4020730524
C	31.8497987664	54.2071053940	64.1289436661
C	32.0518160600	54.6215069063	65.5319957875
O	31.3642351627	54.4312464395	66.4911456974
N	36.6737662091	61.0713307278	61.3202970532
C	35.6004762983	61.3926596572	60.5350959841
C	36.0523635474	62.3833950912	59.4918933081
C	37.4877311436	62.7742792324	59.9326180598
C	37.8222582204	61.7665120962	61.0357019928
O	34.5075125816	60.9304732535	60.7004234999
C	39.0279287087	61.6672266439	61.6623506863
N	38.8411543107	59.7554076031	63.2793445323
C	39.4298322028	60.8949309891	62.8096532499
C	40.4846965035	61.1946375049	63.6883090492
C	40.5055862253	60.2226214843	64.6775901388
C	39.4655658279	59.3153287589	64.4144774198
H	39.7829513204	62.3475529192	61.3049215536
H	34.9261312611	56.6526977922	62.9757181467
H	39.7445059215	58.0815769644	66.1030425497
H	38.2545089318	59.1804290922	62.7016249267
H	36.5806460240	60.3251972322	61.9766772421
H	33.6105601523	55.7330779951	66.3929844008
H	37.0593235594	57.6468018752	63.3763463747
H	38.2044768591	62.6599436951	59.1269388668
H	35.3546566898	63.2107426069	59.4822838068

H	38.2512282200	56.2874408865	67.2168566259
H	36.2484284499	54.7759422461	66.3479821744
H	33.0638635333	54.5758221473	62.3382493628
H	30.9828780634	53.6172696070	63.8312705390
H	35.9839761752	61.9191962145	58.5080693291
H	37.5675694848	63.7951658198	60.3060929135
H	41.1727266381	62.0358990785	63.6048791388
H	41.2044099964	60.1757575383	65.5127644673
H	36.9040200074	57.8166242133	58.3507155240
H	35.5739833868	57.2186406116	59.4818784394
H	39.5823941907	55.1304233222	61.3530699675
H	40.4081895334	56.3112166529	60.3142871596
54			
-140			
C	36.4851025876	57.8035433231	59.3550118881
H	36.2030475829	58.8147340742	59.6113411297
C	37.4981917207	57.4013538437	60.4186981325
O	37.5397982699	57.9657690216	61.5110520287
N	38.3760106684	56.4158561159	60.1486407080
H	38.3164165077	55.9711637299	59.2583260490
C	39.5374059303	56.1418423802	60.9481144172
H	39.5590433169	56.8226861567	61.7809090151
C	39.0968506523	58.2015323514	65.2609556124
N	37.0632279580	57.2357465123	64.2813099239
C	38.0397099246	57.3636240473	65.2290921255
C	37.7098779801	56.3605749797	66.2714426161
C	36.6235226128	55.6844239777	65.8885184857
C	36.1049937657	56.3045785187	64.6470818754
C	34.9328081443	56.2111081851	63.9596250268
N	33.2258363495	55.3585661494	65.5363577509
C	33.7454819709	55.4379558112	64.2909983916
C	32.8797761022	54.6839170393	63.4044767317
C	31.8573990085	54.1931328779	64.1265878237
C	32.0437296925	54.6288244456	65.5248593741
O	31.3525620171	54.4481086871	66.4817425664
N	36.6728948620	61.0727823463	61.3224637757
C	35.6015758167	61.3925841175	60.5351718079
C	36.0523029086	62.3838761586	59.4917067350
C	37.4871796548	62.7740991228	59.9337939419
C	37.8221729673	61.7664233370	61.0378203575
O	34.5091108956	60.9292119613	60.6996956818
C	39.0254211842	61.6633843794	61.6624326046
N	38.8365678093	59.7567671372	63.2858561197
C	39.4268110065	60.8933957704	62.8130138415

C	40.4819218220	61.1922369867	63.6890154812
C	40.5051970536	60.2182085307	64.6784800456
C	39.4659796488	59.3108108377	64.4188824396
H	39.7775131756	62.3438604524	61.3087144781
H	34.8872105824	56.7417229535	63.0269583687
H	39.7387448158	58.0847613644	66.1049847639
H	38.2599078855	59.1784831235	62.7023754613
H	36.5787429624	60.3259482787	61.9783174473
H	33.5904301960	55.7695737361	66.3855744087
H	37.0734416324	57.6332552113	63.3694720186
H	38.2032535030	62.6581196692	59.1294450878
H	35.3520514241	63.2089579314	59.4841776252
H	38.2536481068	56.2809916627	67.2127436287
H	36.2529993644	54.7639980413	66.3397342540
H	33.0752819049	54.5594767138	62.3394137580
H	30.9858917627	53.6091184431	63.8308357115
H	35.9831675813	61.9184991187	58.5084918202
H	37.5670058697	63.7952352020	60.3065887038
H	41.1697002362	62.0335960323	63.6045000354
H	41.2045458694	60.1746023922	65.5133912924
H	36.9045809584	57.8167960259	58.3490649145
H	35.5735504107	57.2195620088	59.4819193538
H	39.5828439924	55.1310758061	61.3535196055
H	40.4079022923	56.3125401648	60.3147382244
54			
-135			
C	36.4838064023	57.7972375735	59.3462401663
H	36.2060530579	58.8078021941	59.6071676082
C	37.4944175979	57.3946735642	60.4124712799
O	37.5361685868	57.9634192273	61.5014575715
N	38.3687272369	56.4037265315	60.1422323087
H	38.3178062207	55.9662963337	59.2459370288
C	39.5327888971	56.1378420121	60.9420847568
H	39.5519715340	56.8230483077	61.7720563395
C	39.0814958449	58.2181985074	65.2627381879
N	37.0593367298	57.2387298620	64.2731304198
C	38.0280498187	57.3718376907	65.2245587514
C	37.7064806032	56.3646027088	66.2638433071
C	36.6280879071	55.6789447072	65.8783425939
C	36.0997985475	56.3076600562	64.6445165539
C	34.9100083678	56.2551550083	63.9941564837
N	33.2042734762	55.3812375674	65.5482348353
C	33.7345464111	55.4523525617	64.3132916564
C	32.8981490607	54.6752258212	63.4214369542

C	31.8688199430	54.1817485947	64.1326700894
C	32.0376929991	54.6202319101	65.5348064262
O	31.3535602092	54.4202830208	66.4915609843
N	36.6749217363	61.0819802966	61.3286035357
C	35.6067757678	61.3969491902	60.5369982493
C	36.0598397818	62.3864898276	59.4920965028
C	37.4945415624	62.7768117854	59.9350168731
C	37.8293734331	61.7695951226	61.0381872957
O	34.5140634298	60.9340206548	60.6968432599
C	39.0337737467	61.6593566557	61.6538654207
N	38.8279109645	59.7711121534	63.2899340606
C	39.4295851790	60.8989191574	62.8164779864
C	40.4871386915	61.1915834354	63.6875170404
C	40.5049737946	60.2168613874	64.6781939581
C	39.4564036305	59.3213047587	64.4246117805
H	39.7892279693	62.3293840078	61.2888333573
H	34.8311979611	56.8380376057	63.0963760535
H	39.7162776782	58.1022105674	66.1124812059
H	38.2618830269	59.1892396473	62.6996311378
H	36.5804718979	60.3324565177	61.9810120283
H	33.5712555189	55.7815152363	66.4031380843
H	37.0771377677	57.6261342857	63.3560990331
H	38.2121661842	62.6603250857	59.1321178598
H	35.3570707445	63.2097544314	59.4861042913
H	38.2510281384	56.2835908079	67.2045728672
H	36.2591553321	54.7592571932	66.3323587645
H	33.1054491789	54.5572767891	62.3578666752
H	30.9998577024	53.5989727687	63.8271475656
H	35.9888313468	61.9205099278	58.5093007018
H	37.5709273757	63.7977749298	60.3090041948
H	41.1734993976	62.0344605834	63.6066829766
H	41.2024892672	60.1684451078	65.5143728150
H	36.9039496289	57.8124299300	58.3405981262
H	35.5727956075	57.2134779388	59.4779636342
H	39.5823388003	55.1284730089	61.3504792643
H	40.4035623075	56.3101800277	60.3095341048
54			
-130			
C	36.4833296353	57.7681801308	59.3489381634
H	36.1845910678	58.7783004637	59.5910269365
C	37.5083859520	57.3927317070	60.4154401999
O	37.5684458718	57.9836320908	61.4918648912
N	38.3775192099	56.3927310025	60.1531322838
H	38.2989083907	55.9051122034	59.2830044515

C	39.5464870122	56.1386780064	60.9509239552
H	39.5610347232	56.8269071898	61.7785416308
C	39.1082015243	58.2035870441	65.1829166992
N	37.0304989717	57.2792767375	64.2374251957
C	38.0312550562	57.3792575979	65.1601402468
C	37.7134585581	56.3767052736	66.2019003467
C	36.5956106758	55.7309749225	65.8578478967
C	36.0533113002	56.3689486750	64.6296910934
C	34.8579433142	56.3173559430	63.9889194747
N	33.1207094050	55.4690850766	65.5402955418
C	33.6896432761	55.4918381559	64.3230909988
C	32.9087709240	54.6342439862	63.4535361080
C	31.8848392042	54.1244319301	64.1639111651
C	31.9819356344	54.6513997749	65.5449670076
O	31.2800643456	54.4824498908	66.4937851106
N	36.7052683669	61.1724014018	61.3409355139
C	35.6352507038	61.4765094831	60.5465105369
C	36.1034738980	62.4436461715	59.4810618012
C	37.5327129342	62.8511631667	59.9315538708
C	37.8685513255	61.8541161577	61.0416600798
O	34.5351971386	61.0262810014	60.7145277031
C	39.0733103416	61.7377306107	61.6531701144
N	38.8734854086	59.7867156966	63.2238731897
C	39.4619622700	60.9419059208	62.8016371425
C	40.5036210189	61.2205802157	63.6948072337
C	40.5211819445	60.2151402126	64.6547952286
C	39.4841194266	59.3170875351	64.3637567467
H	39.8412943881	62.3975280642	61.2896648469
H	34.7552031362	56.9117217824	63.0989019782
H	39.7679193243	58.0553803658	66.0107122062
H	38.2831172361	59.2277698514	62.6287329815
H	36.6022202449	60.4645195348	62.0370044834
H	33.4519939749	55.9209696181	66.3831360678
H	37.0342141081	57.6816710919	63.3247871543
H	38.2552434737	62.7462453258	59.1310399704
H	35.4043218200	63.2682393984	59.4377566876
H	38.2817277896	56.2816345845	67.1271563631
H	36.1983208194	54.8545572787	66.3698243953
H	33.1572749794	54.4582324342	62.4069546637
H	31.0813829272	53.4543923467	63.8580157270
H	36.0571635388	61.9571541449	58.5067682571
H	37.5989180087	63.8723919360	60.3067548040
H	41.1894076701	62.0664606270	63.6473725410
H	41.2105942811	60.1547460782	65.4968939343

H	36.8980136439	57.7798545841	58.3409858175
H	35.5868501149	57.1644099536	59.4898391717
H	39.6161676639	55.1338243938	61.3674343098
H	40.4093315467	56.3148346047	60.3086349132
54			
-125			
C	36.4755726754	57.7578987717	59.3398591553
H	36.1812204592	58.7657310843	59.5970540257
C	37.4940977322	57.3748707984	60.4069188221
O	37.5445045859	57.9633839599	61.4840384294
N	38.3615094280	56.3709867822	60.1524808147
H	38.3076994744	55.9201891675	59.2628221822
C	39.5399698456	56.1361430534	60.9416674367
H	39.5530246986	56.8288063468	61.7651806085
C	39.1221648801	58.1754391538	65.1524450609
N	36.9984988449	57.3194489762	64.2608340545
C	38.0294712568	57.3691225139	65.1474578862
C	37.7272360699	56.3490468480	66.1730077146
C	36.5909654534	55.7289432396	65.8452700305
C	36.0257746380	56.4083591123	64.6584792234
C	34.8136147169	56.3882504996	64.0611581865
N	33.0499092266	55.5429837446	65.5817935747
C	33.6597330697	55.5442012442	64.3910089914
C	32.9286755644	54.6659531598	63.5063123227
C	31.8787143858	54.1618282131	64.1760138427
C	31.9162716026	54.7153202879	65.5475416175
O	31.1816189053	54.5527607841	66.4700545721
N	36.7056059545	61.1874200979	61.3521338655
C	35.6478555473	61.4815083391	60.5437023851
C	36.1129667148	62.4452608796	59.4780540477
C	37.5389701224	62.8527538693	59.9331146915
C	37.8711536953	61.8568183675	61.0453817925
O	34.5492667760	61.0261601717	60.7033669422
C	39.0742669864	61.7234370803	61.6485318633
N	38.8585275542	59.7936786972	63.2354794861
C	39.4573910211	60.9388544547	62.8073753766
C	40.5010392846	61.2116751627	63.6939676944
C	40.5244620173	60.1983994405	64.6486349536
C	39.4869457819	59.3046774546	64.3585602186
H	39.8438680685	62.3743913780	61.2806945945
H	34.6832338322	57.0054638650	63.1925671277
H	39.8002595792	57.9932696162	65.9562716309
H	38.3027060223	59.2250100395	62.6213962058
H	36.5942176629	60.4933707772	62.0603118325

H	33.3704004209	55.9791593961	66.4392428237
H	37.0089653495	57.7002324038	63.3399760026
H	38.2646879602	62.7440143830	59.1378977231
H	35.4056424245	63.2639965291	59.4397293901
H	38.3008235634	56.2394490000	67.0933624018
H	36.1913939342	54.8388127243	66.3311479144
H	33.2551311515	54.4580143582	62.4873642102
H	31.0996748350	53.4760078101	63.8431252661
H	36.0641955216	61.9557863799	58.5053757163
H	37.6015100049	63.8748784771	60.3065006859
H	41.1785598707	62.0644852846	63.6521609638
H	41.2101511815	60.1420156434	65.4940450841
H	36.8929212909	57.7722601090	58.3330419244
H	35.5782898483	57.1561522328	59.4842602715
H	39.6191652399	55.1326094969	61.3596573192
H	40.4013389146	56.3159702465	60.2984158849
54			
-120			
C	36.4752462509	57.7546264097	59.3397567079
H	36.1835945366	58.7632315017	59.5968667546
C	37.4918948762	57.3711925462	60.4054323268
O	37.5422125787	57.9617470848	61.4822149558
N	38.3582669946	56.3672088434	60.1497485103
H	38.3132938181	55.9257122528	59.2545796810
C	39.5357492576	56.1355508751	60.9391182363
H	39.5482178571	56.8295477877	61.7616248188
C	39.1191254251	58.1764668360	65.1511929073
N	36.9968790714	57.3189517567	64.2591125777
C	38.0286303446	57.3650213646	65.1433579457
C	37.7338883497	56.3397171831	66.1629985350
C	36.6017329980	55.7126428242	65.8309567205
C	36.0312128624	56.3999973421	64.6528622320
C	34.8034457543	56.4121889498	64.0863453668
N	33.0235024661	55.5686355729	65.5789196023
C	33.6509797487	55.5538401922	64.4005743275
C	32.9485532212	54.6460492339	63.5196181757
C	31.8867810394	54.1543772570	64.1791648410
C	31.9032837933	54.7261586226	65.5438084917
O	31.1653858010	54.5613831096	66.4640371130
N	36.7016124452	61.1896938273	61.3550370959
C	35.6465956344	61.4806987470	60.5442964057
C	36.1136997068	62.4434005637	59.4793580242
C	37.5405138488	62.8517289628	59.9342162534
C	37.8696778750	61.8572838512	61.0486584036

O	34.5474126881	61.0236463680	60.7012171073
C	39.0734026865	61.7245089270	61.6493159860
N	38.8512467384	59.7983828003	63.2388175869
C	39.4540144731	60.9416924877	62.8133370823
C	40.4992052425	61.2112189926	63.6954818440
C	40.5220512454	60.1956315247	64.6495054508
C	39.4815503927	59.3075858143	64.3618276653
H	39.8466374474	62.3718012590	61.2763851802
H	34.6512249386	57.0790812769	63.2567388859
H	39.8005569337	57.9876074439	65.9526409718
H	38.3071133233	59.2265921551	62.6168943398
H	36.5920325773	60.4961903477	62.0630162991
H	33.3458766046	56.0066797048	66.4337823314
H	37.0088092588	57.6917327910	63.3345785032
H	38.2701390706	62.7425561307	59.1406068156
H	35.4073909311	63.2626440916	59.4408741311
H	38.3034721247	56.2344543563	67.0863408940
H	36.1972824661	54.8270206102	66.3210180562
H	33.2754670074	54.4459774360	62.4992428999
H	31.1064373365	53.4719111039	63.8424591732
H	36.0652568981	61.9551447018	58.5060510023
H	37.6024155776	63.8739994001	60.3073092450
H	41.1771058004	62.0636635737	63.6523991201
H	41.2090681348	60.1392907445	65.4938398405
H	36.8922440747	57.7704758502	58.3328164606
H	35.5770862792	57.1540726316	59.4836694463
H	39.6181139796	55.1328091490	61.3583939626
H	40.3977390911	56.3157818549	60.2968119447
54			
-115			
C	36.4730300968	57.7561203745	59.3387874103
H	36.1796936370	58.7642511309	59.5961201646
C	37.4910436072	57.3724044413	60.4058099930
O	37.5398796243	57.9605450065	61.4840632085
N	38.3595695171	56.3692155841	60.1506195965
H	38.3094403449	55.9220070817	59.2583647983
C	39.5379492117	56.1361527038	60.9397408821
H	39.5512605348	56.8293242336	61.7634574262
C	39.1228263645	58.1713445724	65.1464564629
N	37.0033789695	57.3085624632	64.2519362318
C	38.0341020543	57.3544099631	65.1372563109
C	37.7413373543	56.3276298444	66.1525831595
C	36.6118704223	55.6956953089	65.8139496167
C	36.0383851547	56.3895569038	64.6420641109

C	34.7922907721	56.4380635327	64.1145122397
N	32.9970514838	55.5990381502	65.5770093264
C	33.6454747065	55.5659458869	64.4126455197
C	32.9651776217	54.6353850963	63.5348146680
C	31.8939896876	54.1479670542	64.1830885268
C	31.8876432669	54.7373353929	65.5425537661
O	31.1447445210	54.5743416215	66.4575692745
N	36.7040180518	61.1911847616	61.3551354913
C	35.6492516483	61.4811466290	60.5449402538
C	36.1138102933	62.4441111408	59.4784886630
C	37.5399414756	62.8531750836	59.9331896335
C	37.8726498784	61.8589929875	61.0463711861
O	34.5493106098	61.0238605495	60.7021052790
C	39.0749832954	61.7215884758	61.6456619041
N	38.8589442912	59.7925756513	63.2336273701
C	39.4571100006	60.9377751232	62.8117588182
C	40.4970797522	61.2111374704	63.6964399409
C	40.5224023533	60.1929717823	64.6494154567
C	39.4882753752	59.3016044530	64.3576414825
H	39.8478340859	62.3678212435	61.2737032260
H	34.6162516100	57.1454937247	63.3251404251
H	39.8026849601	57.9871499417	65.9516604929
H	38.3076281329	59.2249809515	62.6155528929
H	36.5948870027	60.4978077136	62.0636245547
H	33.3140452804	56.0363729090	66.4357718151
H	37.0121492049	57.6952613197	63.3328592477
H	38.2659079403	62.7443609814	59.1376503778
H	35.4059370081	63.2627045639	59.4397157551
H	38.3071830754	56.2261105250	67.0786390429
H	36.2027680753	54.8143511102	66.3078471005
H	33.2961900878	54.4382203261	62.5151952037
H	31.1117519379	53.4702615120	63.8412020655
H	36.0651936365	61.9553588041	58.5054395247
H	37.6014489839	63.8755336316	60.3061063494
H	41.1745390283	62.0639775865	63.6542532469
H	41.2088563858	60.1381028470	65.4943043919
H	36.8907033913	57.7715065279	58.3321199693
H	35.5755214049	57.1547039966	59.4831603296
H	39.6194553003	55.1327504536	61.3576017840
H	40.3996343747	56.3162207587	60.2969802526
54			
-110			
C	36.4723967828	57.7573060588	59.3387364061
H	36.1774193805	58.7655431955	59.5952384076

C	37.4915865333	57.3734840045	60.4045491879
O	37.5387709101	57.9573263616	61.4840334894
N	38.3618591223	56.3719711383	60.1511787735
H	38.3071055509	55.9207983462	59.2624573726
C	39.5393690469	56.1367976274	60.9398485176
H	39.5521879995	56.8285350783	61.7639849339
C	39.1252629900	58.1680111042	65.1446452201
N	37.0120682213	57.2943605944	64.2430818398
C	38.0398959270	57.3445581074	65.1324703094
C	37.7455788388	56.3205520352	66.1454581454
C	36.6202066285	55.6818697983	65.7983609875
C	36.0480065818	56.3737889998	64.6261597014
C	34.7851807356	56.4538832818	64.1347330032
N	32.9749408519	55.6277272828	65.5683924291
C	33.6421280494	55.5721460470	64.4171016480
C	32.9818092639	54.6130628392	63.5487111735
C	31.8990344837	54.1399299695	64.1879316307
C	31.8796170970	54.7461300963	65.5396554192
O	31.1374442494	54.5812115902	66.4533173027
N	36.7046149972	61.1913029262	61.3549240695
C	35.6495106135	61.4811070129	60.5449584564
C	36.1131950843	62.4439896356	59.4776055329
C	37.5385819845	62.8530114201	59.9319010718
C	37.8731368806	61.8596958052	61.0458860490
O	34.5506072035	61.0242507994	60.7026096550
C	39.0748623567	61.7225016898	61.6439928339
N	38.8637371029	59.7894746385	63.2311706422
C	39.4600769983	60.9354270146	62.8093784120
C	40.4947027697	61.2110662563	63.6967656407
C	40.5203400521	60.1909802179	64.6507648730
C	39.4925366037	59.2967644974	64.3560085725
H	39.8488996622	62.3660873952	61.2725699583
H	34.5928433160	57.2022351682	63.3882198244
H	39.8017278580	57.9875764421	65.9524564486
H	38.3078579657	59.2249628075	62.6158014215
H	36.5964211229	60.4983876099	62.0639339988
H	33.2997474857	56.0578118758	66.4281994707
H	37.0108613390	57.7022043751	63.3328133569
H	38.2635954149	62.7453455924	59.1359615207
H	35.4061614405	63.2629855688	59.4386016916
H	38.3083207106	56.2223520358	67.0737605731
H	36.2068697810	54.8059708025	66.2983816382
H	33.3099999469	54.4261948356	62.5262444131
H	31.1136739107	53.4681476139	63.8415441474

H	36.0649192469	61.9552973209	58.5045092804
H	37.6006102435	63.8752744627	60.3049933100
H	41.1726321848	62.0635495289	63.6549203349
H	41.2080411462	60.1371351368	65.4947050153
H	36.8899888345	57.7721337027	58.3320268796
H	35.5752948865	57.1552821763	59.4831056682
H	39.6198647798	55.1329640329	61.3568684250
H	40.4009350282	56.3164737740	60.2968185077
54			
-105			
C	36.4742923475	57.7514683281	59.3313653895
H	36.1819237631	58.7605407396	59.5879779819
C	37.4904579206	57.3625392273	60.3972726043
O	37.5349260639	57.9337983376	61.4856899598
N	38.3622453468	56.3657038000	60.1331472882
H	38.3268271162	55.9427322151	59.2279566107
C	39.5331312779	56.1329385828	60.9314491352
H	39.5321053046	56.8288660392	61.7529934022
C	39.1126787454	58.1734539393	65.1449713551
N	36.9858973943	57.3141370861	64.2612027502
C	38.0273035271	57.3521383801	65.1363426748
C	37.7529431664	56.3122450401	66.1353431577
C	36.6305774834	55.6659125558	65.7872843333
C	36.0439635951	56.3736386539	64.6356679053
C	34.7667859496	56.4747662215	64.1777765881
N	32.9369603657	55.6557135013	65.5704332034
C	33.6289796528	55.5853867643	64.4359919328
C	33.0016005944	54.6015625657	63.5697908670
C	31.9085803124	54.1325332154	64.1946322981
C	31.8529760878	54.7609584266	65.5394016777
O	31.0995986429	54.6003551875	66.4423310272
N	36.6938957902	61.1975624385	61.3662812505
C	35.6474190950	61.4781459246	60.5432035120
C	36.1174265852	62.4402206374	59.4771255191
C	37.5400332060	62.8493316860	59.9391009249
C	37.8640313457	61.8636961142	61.0590019814
O	34.5483523094	61.0147182687	60.6910500221
C	39.0658525684	61.7305694980	61.6555596097
N	38.8450538221	59.8019651504	63.2392803651
C	39.4479051322	60.9455531945	62.8215197913
C	40.4858374835	61.2145033258	63.7019451837
C	40.5085696380	60.1926324548	64.6551753340
C	39.4754562074	59.3082927185	64.3631280764
H	39.8439563027	62.3614208453	61.2701320184

H	34.5590734282	57.2650198736	63.4802688356
H	39.8113667539	57.9696392653	65.9297247401
H	38.3073282304	59.2312545964	62.6120652667
H	36.5830298459	60.5063747471	62.0777248976
H	33.2610580386	56.0855063721	66.4305786823
H	36.9843003309	57.7062924024	63.3428751698
H	38.2736830398	62.7339038033	59.1523177617
H	35.4147823055	63.2627768988	59.4366654393
H	38.3154036682	56.2172171776	67.0641461189
H	36.2163507557	54.7901042650	66.2867271299
H	33.3387919283	54.4165154582	62.5499248837
H	31.1245797479	53.4630734131	63.8407485045
H	36.0692647228	61.9523036195	58.5036346511
H	37.6029395928	63.8721089597	60.3106335429
H	41.1685267463	62.0628841365	63.6544854986
H	41.2002514718	60.1328591976	65.4954553171
H	36.8929535928	57.7670826510	58.3251119393
H	35.5762064146	57.1516097153	59.4786010963
H	39.6179081203	55.1312860069	61.3528421296
H	40.3946239630	56.3153863141	60.2891016979
54			
-100			
C	36.4718181257	57.7514927596	59.3312803438
H	36.1792489275	58.7602839919	59.5889351380
C	37.4877030088	57.3625734536	60.3994198360
O	37.5289469970	57.9301684805	61.4884727256
N	38.3622828685	56.3677647623	60.1328669046
H	38.3257413252	55.9435677231	59.2290790448
C	39.5333884096	56.1331588368	60.9314464207
H	39.5329470490	56.8279290167	61.7537596719
C	39.1181204953	58.1631429021	65.1395567653
N	36.9886401486	57.3030081519	64.2598502697
C	38.0353288871	57.3399916303	65.1326622983
C	37.7646344433	56.2966534361	66.1262808230
C	36.6424406381	55.6496271636	65.7760169599
C	36.0492345993	56.3648338514	64.6347829928
C	34.7570520713	56.4884560320	64.2151491396
N	32.8960140859	55.6932464933	65.5687727767
C	33.6173752509	55.6019966839	64.4532841339
C	33.0140466688	54.5959483245	63.5898798965
C	31.9097084987	54.1360301992	64.1977393878
C	31.8274774778	54.7792346190	65.5360636205
O	31.0697364895	54.6091785377	66.4336970105
N	36.6947032002	61.1984954262	61.3660774936

C	35.6477026357	61.4761283599	60.5422975825
C	36.1173174269	62.4396645552	59.4763374749
C	37.5399701916	62.8500267998	59.9370560900
C	37.8665190575	61.8648882855	61.0566889140
O	34.5492312535	61.0112739896	60.6881338990
C	39.0674247880	61.7293937743	61.6514521611
N	38.8487156999	59.7970957751	63.2355613348
C	39.4499919495	60.9427991291	62.8194675772
C	40.4829681058	61.2140852878	63.7016168329
C	40.5060563428	60.1903565983	64.6563992506
C	39.4790043780	59.3036380525	64.3607596231
H	39.8482528487	62.3569763444	61.2648641037
H	34.5338611735	57.3127192776	63.5634987699
H	39.8202271352	57.9584993924	65.9226579508
H	38.3120847822	59.2261524077	62.6082491792
H	36.5863904628	60.5088218110	62.0788096121
H	33.2265642921	56.1034420225	66.4375155196
H	36.9752307042	57.7189491216	63.3522065715
H	38.2728143345	62.7362942872	59.1494495383
H	35.4143646064	63.2619863196	59.4371947910
H	38.3246483891	56.2046441859	67.0568643656
H	36.2265889441	54.7744881378	66.2752825261
H	33.3594943253	54.4140973071	62.5722045183
H	31.1293618948	53.4663888591	63.8362055655
H	36.0690390347	61.9523491048	58.5025511020
H	37.6021628813	63.8727884331	60.3087518826
H	41.1657173218	62.0624308122	63.6543893969
H	41.1981687114	60.1319665979	65.4964219458
H	36.8908800892	57.7677183909	58.3252033830
H	35.5738221728	57.1512442486	59.4774721528
H	39.6182522994	55.1308933971	61.3513620788
H	40.3948640079	56.3154132280	60.2890211863
AnPixJ_-145_-100_S1_SCAN_C14-C15-C16-C17			
49			
-145			
C	25.2698054552	51.4744560172	30.2912774437
H	25.5798045997	51.1184395677	31.2639214975
H	25.2289597138	50.6545270662	29.5878904411
C	26.2097138050	52.5577422575	29.7907668511
O	26.9909352900	53.0704702043	30.6647578419
O	26.1795483809	52.9047733638	28.6264334449
C	30.1016935365	54.0609915901	28.3228148773
N	29.5234424296	52.3452924803	29.9935599916

C	30.1981411128	52.8473200099	28.9003547850
C	31.1316740506	51.7914314850	28.5006535207
C	31.0124246272	50.7644955370	29.3624759854
C	29.9405069951	51.0891976530	30.3078872088
C	29.2795238144	50.3402840254	31.2823785228
N	31.0682475448	48.9656654191	32.2980008814
C	29.7521633290	49.1637397670	31.9585814390
C	29.0405683195	47.9939260132	32.4192108180
C	29.9099834700	47.1710646312	33.0378163748
C	31.2343469482	47.8219294107	33.0394153598
O	32.2731842964	47.5038876846	33.5641864722
N	25.8932840948	55.3373615314	31.7556272253
C	25.0445186218	55.1319392709	32.8157213517
C	24.2905517346	56.4140680484	33.0615017612
C	24.6403790026	57.3094333898	31.8424770832
C	25.7694902668	56.5461820551	31.1509735738
O	24.9915531330	54.0910002647	33.4077587860
C	26.4769089653	57.0130201187	30.0749861368
N	28.2167096928	55.3069859934	29.4379482348
C	27.5939378330	56.5269885275	29.3369113206
C	28.3047194516	57.2552122324	28.3562909417
C	29.3292967338	56.4598907012	27.8847780366
C	29.2491855968	55.2245527614	28.5648272775
H	26.1647464748	57.9826132017	29.7450807178
H	28.3034017944	50.6892379386	31.5523400507
H	30.8028484651	54.2362977528	27.5366011635
H	27.7946994824	54.4949589789	29.8724785111
H	26.4189912890	54.5428048792	31.4101952470
H	31.7855176039	49.6640461276	32.2691979217
H	28.6544239849	52.7107860598	30.3554349200
H	23.8084021847	57.3339032556	31.1410315978
H	24.6732395372	56.8119982033	33.9966739192
H	24.2456130754	51.8313641382	30.3995068933
H	31.6960097773	51.8020335275	27.5681944944
H	31.5371732179	49.8107196797	29.3075434646
H	27.9766412721	47.8276521071	32.2504527745
H	29.7579326312	46.1878118709	33.4829698351
H	23.2249563984	56.2516768847	33.2233743675
H	24.8658983761	58.3360632306	32.1309891865
H	28.0509521567	58.2597614102	28.0178383441
H	30.0461125477	56.7309650303	27.1096903975

49

-140

C	25.2686175989	51.4765019452	30.2892752264

H	25.5810526748	51.1206912640	31.2612283561
H	25.2282892222	50.6568111652	29.5858201217
C	26.2068798098	52.5603607926	29.7886333068
O	26.9864894650	53.0739073659	30.6630466946
O	26.1792793654	52.9057599322	28.6232654289
C	30.0855826120	54.0527302167	28.3145457672
N	29.5315253836	52.3502537181	30.0049595655
C	30.1877838379	52.8431619952	28.9012426257
C	31.1165227345	51.7857613356	28.5016627320
C	31.0066957133	50.7599678659	29.3689748955
C	29.9546339430	51.0975071918	30.3230028195
C	29.3198327571	50.3800448650	31.3387392760
N	31.0894823044	48.9691138089	32.3262365131
C	29.7799916233	49.1741484118	31.9810079033
C	29.0590816293	48.0007247187	32.4011326888
C	29.9188131779	47.1655071411	33.0208987708
C	31.2443348890	47.8078692878	33.0424177462
O	32.2806823378	47.4771743245	33.5635847109
N	25.8961041236	55.3454873512	31.7565887856
C	25.0467674882	55.1395299037	32.8152006378
C	24.2915471399	56.4201127933	33.0608379173
C	24.6419071153	57.3149187509	31.8413836075
C	25.7716297442	56.5524694419	31.1492727214
O	24.9950436537	54.0991821460	33.4077372519
C	26.4791056227	57.0158017820	30.0722245877
N	28.2160936481	55.3034039824	29.4343914258
C	27.5940388005	56.5238451665	29.3348102574
C	28.3105136743	57.2515805684	28.3510666885
C	29.3282515592	56.4542225811	27.8784494078
C	29.2440180958	55.2153615642	28.5576215075
H	26.1671345293	57.9868041324	29.7446768515
H	28.3761212309	50.7651770100	31.6702050864
H	30.7784515855	54.2091060240	27.5183732539
H	27.7855698976	54.4935145983	29.8638531532
H	26.4164817902	54.5498058422	31.4083646642
H	31.8156349684	49.6604614893	32.2979106434
H	28.6585993997	52.7105142638	30.3618005626
H	23.8096603709	57.3383398915	31.1406710981
H	24.6737827601	56.8172904317	33.9965274721
H	24.2442190825	51.8326080435	30.3981934429
H	31.6816044417	51.7873124724	27.5695965811
H	31.5389692352	49.8100187529	29.3205122096
H	27.9969500270	47.8428910113	32.2139642312
H	29.7549096149	46.1827149932	33.4628515595

H	23.2261425734	56.2557853354	33.2220106440
H	24.8669540467	58.3417850150	32.1294228510
H	28.0621678316	58.2587800605	28.0164914697
H	30.0466308411	56.7207598195	27.1032357861
49			
-135			
C	25.2697720864	51.4777051763	30.2911929697
H	25.5821947840	51.1220655326	31.2631942580
H	25.2306539607	50.6586518780	29.5870661639
C	26.2092610768	52.5594202958	29.7898405299
O	26.9949469684	53.0672961820	30.6625193632
O	26.1784167715	52.9095247719	28.6258875325
C	30.0793172948	54.0649805687	28.3021153177
N	29.5239843175	52.3486590513	29.9805185866
C	30.1827083444	52.8423203271	28.8857815111
C	31.1013696858	51.7843242304	28.4869076078
C	30.9708714773	50.7461611886	29.3484279769
C	29.9365213634	51.0908426476	30.3011122207
C	29.3157411204	50.4044302610	31.3596049965
N	31.0712191361	48.9930333796	32.3824778857
C	29.7696895230	49.1757337599	31.9995249096
C	29.0635934904	47.9873513005	32.3844795966
C	29.9272060878	47.1576329693	33.0160252568
C	31.2404962500	47.8176315764	33.0725869735
O	32.2792623299	47.4949997597	33.5977541869
N	25.8978477037	55.3412292702	31.7567524715
C	25.0474583628	55.1367247121	32.8151176444
C	24.2923016016	56.4168346082	33.0593287409
C	24.6452042380	57.3103549681	31.8392696904
C	25.7746304426	56.5462788616	31.1470075509
O	24.9956302660	54.0970288658	33.4081032958
C	26.4805137618	57.0069638291	30.0701660018
N	28.2208289151	55.2996361864	29.4411404508
C	27.5973931805	56.5178924308	29.3413850165
C	28.3124348278	57.2504100663	28.3488684599
C	29.3221753886	56.4587543429	27.8711854999
C	29.2393087587	55.2093352248	28.5520831054
H	26.1714454329	57.9784258572	29.7466471954
H	28.3932362606	50.8258420409	31.7177881751
H	30.7787082560	54.2387072455	27.5157614918
H	27.7804903489	54.4930058762	29.8694914092
H	26.4173370241	54.5451089316	31.4112001943
H	31.7976240755	49.6822088347	32.3425052804
H	28.6626472770	52.7198723531	30.3482411428

H	23.8134894790	57.3320256918	31.1385595122
H	24.6738969015	56.8132505353	33.9956345140
H	24.2450378257	51.8329843900	30.3996523014
H	31.6745992108	51.7868619689	27.5598323558
H	31.5028145061	49.7954399146	29.3133177994
H	28.0020444345	47.8261082426	32.1969127294
H	29.7641471140	46.1743618776	33.4572245118
H	23.2269919176	56.2531836939	33.2218116842
H	24.8689843602	58.3370399753	32.1289374385
H	28.0585583449	58.2570537134	28.0167805941
H	30.0437589719	56.7296177748	27.1004603966
49			
-130			
C	25.2698580428	51.4784633873	30.2929745989
H	25.5831938590	51.1231472823	31.2649797733
H	25.2299646420	50.6590515861	29.5901411911
C	26.2059577057	52.5601506694	29.7862751313
O	26.9899160363	53.0730587887	30.6579878748
O	26.1755756003	52.9036141164	28.6205981567
C	30.0742621104	54.0643607833	28.2985845012
N	29.5078390850	52.3366597439	29.9636519201
C	30.1710271330	52.8305596180	28.8776191074
C	31.0931627648	51.7785806697	28.4830570829
C	30.9596099088	50.7364721986	29.3469172980
C	29.9279050771	51.0813692525	30.2903400935
C	29.3307781497	50.4240312721	31.3854888176
N	31.0694430964	49.0117607045	32.4235300225
C	29.7754714849	49.1865825811	32.0209918148
C	29.0725120504	47.9881233999	32.3675622276
C	29.9325909877	47.1535265657	32.9989793342
C	31.2397151954	47.8191922101	33.0811325606
O	32.2795170926	47.4861820941	33.5985145512
N	25.8960792229	55.3411697855	31.7550210557
C	25.0469898669	55.1382218448	32.8138382208
C	24.2923032757	56.4176694852	33.0591078569
C	24.6457958236	57.3101668675	31.8393291507
C	25.7741227535	56.5452213820	31.1449997848
O	24.9981378914	54.0989128811	33.4074688179
C	26.4818535454	57.0066892163	30.0704580682
N	28.2193601110	55.2985483364	29.4390985520
C	27.5964751114	56.5151523045	29.3405519242
C	28.3159893631	57.2485873824	28.3475059996
C	29.3220958578	56.4572328206	27.8699332917
C	29.2376138519	55.2019594328	28.5482326919

H	26.1759569223	57.9791206335	29.7490948706
H	28.4442421788	50.8828655003	31.7853155982
H	30.7833385912	54.2386103217	27.5215581776
H	27.7829714102	54.4960847025	29.8768821814
H	26.4173696375	54.5456359728	31.4116970244
H	31.8002335527	49.6960101551	32.3778655926
H	28.6484668142	52.7114916716	30.3362051345
H	23.8134519357	57.3339048479	31.1404931324
H	24.6733473785	56.8147761968	33.9951755547
H	24.2448411114	51.8330881130	30.4009034903
H	31.6696444148	51.7817615143	27.5580025760
H	31.4988427944	49.7897703367	29.3145053967
H	28.0109230156	47.8291523190	32.1782900210
H	29.7649593375	46.1718150546	33.4419343998
H	23.2270378884	56.2534465870	33.2213038676
H	24.8693638746	58.3369802490	32.1287054759
H	28.0625823522	58.2556245591	28.0162537347
H	30.0440078526	56.7271356386	27.0991787317
49			
-125			
C	25.2706168816	51.4780925011	30.2935373445
H	25.5839588526	51.1234636600	31.2655457953
H	25.2313624762	50.6586136318	29.5900356961
C	26.2095984143	52.5592289107	29.7912659071
O	26.9945161456	53.0689490162	30.6641566002
O	26.1802864995	52.9082195123	28.6270532234
C	30.0671037989	54.0579866749	28.2893284649
N	29.5104257775	52.3381493934	29.9651808108
C	30.1676469611	52.8221969040	28.8716030739
C	31.0784494399	51.7678007293	28.4717329163
C	30.9418621887	50.7263841060	29.3397782558
C	29.9258972730	51.0832514377	30.2893075611
C	29.3508459054	50.4464728796	31.4143103550
N	31.0686802111	49.0362534859	32.4834970536
C	29.7842605743	49.2061817255	32.0471072932
C	29.0866239767	47.9918347955	32.3495905149
C	29.9399252108	47.1526733028	32.9850911829
C	31.2426185467	47.8250331834	33.1046598771
O	32.2788667099	47.4828520803	33.6234510098
N	25.9009779223	55.3444472055	31.7551045432
C	25.0504109040	55.1402359964	32.8129676661
C	24.2950850671	56.4202861319	33.0566507670
C	24.6483343328	57.3136937959	31.8365894213
C	25.7787559550	56.5493844700	31.1444842864

O	24.9984695625	54.1009070450	33.4064882967
C	26.4851023085	57.0085197114	30.0690538762
N	28.2192169321	55.2952077936	29.4375582763
C	27.6002809866	56.5144410102	29.3401840415
C	28.3184627139	57.2467819380	28.3453621080
C	29.3218022156	56.4536348455	27.8656175595
C	29.2375417262	55.1974391066	28.5449058114
H	26.1795660785	57.9805050438	29.7454662528
H	28.4991723854	50.9400385369	31.8452567902
H	30.7703425844	54.2256917498	27.5050101321
H	27.7772534702	54.4898812932	29.8663092871
H	26.4203442934	54.5484030204	31.4097135709
H	31.8025298805	49.7169777938	32.4434331275
H	28.6518029598	52.7152397403	30.3376950640
H	23.8169726678	57.3347800918	31.1355823544
H	24.6761290974	56.8167219933	33.9930667475
H	24.2459488988	51.8338558282	30.4010316768
H	31.6581243339	51.7683991173	27.5486707783
H	31.4826893520	49.7802514070	29.3196301332
H	28.0269402050	47.8353435051	32.1479774413
H	29.7661817381	46.1675865508	33.4180858113
H	23.2298413321	56.2559076825	33.2188314128
H	24.8710932371	58.3406067569	32.1262362624
H	28.0676847565	58.2552564894	28.0164904816
H	30.0436488621	56.7234568022	27.0947734884

49

-120

C	25.2647051204	51.4752806084	30.2934347261
H	25.5788040192	51.1226168010	31.2662090031
H	25.2217812638	50.6541305592	29.5925583673
C	26.2058242051	52.5524521658	29.7843971473
O	26.9876294833	53.0697698072	30.6550796173
O	26.1788499615	52.8913987221	28.6167063049
C	30.0355314967	54.0309731245	28.2602317202
N	29.5370582055	52.3616284259	30.0037015684
C	30.1520223977	52.8087652607	28.8707168310
C	31.0451776520	51.7450071679	28.4688371185
C	30.9385572504	50.7263663238	29.3710895307
C	29.9660866770	51.1148365267	30.3422616501
C	29.4333705919	50.4985679578	31.4997440966
N	31.1307386157	49.0475391646	32.5648284812
C	29.8649251240	49.2371871725	32.0890132635
C	29.1458267452	48.0273866945	32.3260316031
C	29.9658150572	47.1571301103	32.9633012468

C	31.2697976861	47.8124334778	33.1497456909
O	32.2851598359	47.4413144781	33.6882732462
N	25.9057176495	55.3512806395	31.7523773856
C	25.0562260694	55.1531738044	32.8116628650
C	24.3015795423	56.4343018995	33.0510523535
C	24.6598982520	57.3243001068	31.8303450011
C	25.7906626059	56.5569204465	31.1413403351
O	25.0046844089	54.1172341529	33.4105195546
C	26.5021133233	57.0129701801	30.0690674470
N	28.2165012779	55.2856500070	29.4281680299
C	27.6111620922	56.5096775146	29.3366353519
C	28.3330805158	57.2362680063	28.3387711079
C	29.3204116460	56.4313250478	27.8482503381
C	29.2257011721	55.1754016305	28.5246178167
H	26.2055851781	57.9882516588	29.7498596847
H	28.6110191401	51.0014571705	31.9744889790
H	30.7160499547	54.1768166000	27.4525663230
H	27.7661838470	54.4838833488	29.8540673543
H	26.4178777128	54.5509094892	31.4065728780
H	31.8775373539	49.7163137382	32.5591047364
H	28.6734718570	52.7323078881	30.3731933347
H	23.8306937944	57.3427465342	31.1273924259
H	24.6788235183	56.8312860409	33.9885721877
H	24.2405530939	51.8325999566	30.4006847212
H	31.6147545873	51.7272445922	27.5396796200
H	31.4708251972	49.7752847607	29.3568858553
H	28.1006671223	47.8928208185	32.0474630495
H	29.7604599600	46.1613204894	33.3560601224
H	23.2359064513	56.2690561510	33.2094876938
H	24.8804119060	58.3523147860	32.1177934301
H	28.0969425066	58.2499132868	28.0149978270
H	30.0458220948	56.6934195267	27.0780859294
49			
-115			
C	25.2668106669	51.4755969460	30.2933408587
H	25.5784351533	51.1225538600	31.2662601213
H	25.2245623016	50.6549376420	29.5906331275
C	26.2133447282	52.5520016409	29.7932949425
O	26.9973928168	53.0619696185	30.6661033924
O	26.1886195650	52.8993663309	28.6279110644
C	30.0302800262	54.0230100782	28.2528677158
N	29.5467698540	52.3630527215	30.0102125947
C	30.1529065819	52.7992661527	28.8675224056
C	31.0323550871	51.7342528787	28.4595865687

C	30.9259167143	50.7163527298	29.3675348928
C	29.9741509090	51.1187077915	30.3469056915
C	29.4649337461	50.5217848449	31.5317272014
N	31.1322326027	49.0621851914	32.6239861246
C	29.8784482698	49.2539550591	32.1166924075
C	29.1624296343	48.0337379441	32.3079734217
C	29.9718014008	47.1579796545	32.9524093427
C	31.2693510827	47.8133268293	33.1783418214
O	32.2788934888	47.4295590054	33.7189317480
N	25.9138684445	55.3548395436	31.7536044520
C	25.0601885431	55.1563259882	32.8108885646
C	24.3055190999	56.4384612859	33.0474814215
C	24.6649925570	57.3279479845	31.8264097557
C	25.7986287563	56.5613499817	31.1417634461
O	25.0026025520	54.1204466002	33.4100778256
C	26.5082875851	57.0176826582	30.0684521757
N	28.2152415787	55.2799972797	29.4259091847
C	27.6157742756	56.5085247023	29.3360415611
C	28.3377914500	57.2329601052	28.3365328061
C	29.3220567554	56.4257259747	27.8448552751
C	29.2257356874	55.1691358812	28.5224340188
H	26.2097594774	57.9912846234	29.7415454337
H	28.6846848331	51.0616862996	32.0349110779
H	30.6996131421	54.1620819162	27.4332518349
H	27.7574402107	54.4741502849	29.8392242562
H	26.4252075116	54.5547585860	31.4048847054
H	31.8831606763	49.7253936696	32.6186176714
H	28.6805888394	52.7300040784	30.3785903194
H	23.8390058556	57.3443735288	31.1183726198
H	24.6820688870	56.8367104560	33.9849756097
H	24.2429539156	51.8339618072	30.3999196112
H	31.6027915582	51.7143119852	27.5310007382
H	31.4570086330	49.7645279868	29.3613636918
H	28.1233620569	47.8989658617	32.0075701816
H	29.7616917067	46.1571539409	33.3295947864
H	23.2394827564	56.2743806504	33.2046791023
H	24.8844360941	58.3562895144	32.1135077934
H	28.1037656610	58.2478187236	28.0150348027
H	30.0463298773	56.6867437449	27.0732561435
49			
-110			
C	25.2745246951	51.4840536800	30.3030430143
H	25.5866919897	51.1362717175	31.2782538798
H	25.2354943711	50.6599422965	29.6050103909

C	26.2165246656	52.5588490153	29.7911941079
O	27.0005773352	53.0783117571	30.6587984785
O	26.1885980855	52.8946408539	28.6227651159
C	30.0220405207	54.0140194575	28.2390459867
N	29.5516947648	52.3683689121	30.0185776413
C	30.1481426322	52.7909940476	28.8658988336
C	31.0233662847	51.7262782345	28.4643377755
C	30.9216374767	50.7136667898	29.3847314019
C	29.9809668667	51.1267823684	30.3628111401
C	29.4998957447	50.5490135697	31.5739605275
N	31.1390717145	49.0757214811	32.6932959985
C	29.9022526268	49.2688837292	32.1464047817
C	29.1874210532	48.0435752078	32.2852459067
C	29.9819943097	47.1565602395	32.9348893184
C	31.2689149624	47.8097266406	33.2111383111
O	32.2667079440	47.4101766818	33.7617286323
N	25.9140154824	55.3553472371	31.7483969797
C	25.0618147803	55.1610457502	32.8065250661
C	24.3085153558	56.4434345076	33.0407616432
C	24.6711669608	57.3307981693	31.8199025466
C	25.8037758843	56.5608683053	31.1360468024
O	25.0076621847	54.1275992035	33.4099178103
C	26.5191641301	57.0165463594	30.0660593483
N	28.2209009040	55.2801383484	29.4158375434
C	27.6233360393	56.5077203890	29.3291249425
C	28.3454917580	57.2321276936	28.3271079409
C	29.3231865518	56.4221780723	27.8296732546
C	29.2248729222	55.1629396059	28.5062555662
H	26.2278536007	57.9939022161	29.7475547456
H	28.7562127515	51.1101311496	32.1079421712
H	30.6900122883	54.1466678233	27.4165674887
H	27.7703216043	54.4793010358	29.8454689743
H	26.4299963005	54.5548801267	31.4073620748
H	31.8932139876	49.7353658398	32.7087925293
H	28.6906616589	52.7427958716	30.3911326550
H	23.8446252445	57.3486622434	31.1134533323
H	24.6839403166	56.8418776937	33.9783251877
H	24.2489935511	51.8389883232	30.4048974355
H	31.5893394107	51.6974385337	27.5332578951
H	31.4476232824	49.7590046529	29.3796139978
H	28.1578890368	47.9059023071	31.9548263991
H	29.7651176298	46.1493559908	33.2906577244
H	23.2420988790	56.2808592206	33.1969417488
H	24.8904065586	58.3592746835	32.1066726808

H	28.1160369366	58.2496828136	28.0108815753
H	30.0477505875	56.6832148615	27.0583537331
49			
-105			
C	25.2758138565	51.4821388579	30.3025496734
H	25.5907613882	51.1336068628	31.2766099903
H	25.2357318438	50.6587342778	29.6043445512
C	26.2125788705	52.5599238406	29.7890875549
O	26.9950920377	53.0814458895	30.6570247713
O	26.1832884041	52.8960592720	28.6204129925
C	30.0192830161	54.0144386875	28.2304740837
N	29.5261386576	52.3602454551	29.9953861521
C	30.1346647274	52.7835062422	28.8513494583
C	31.0131623415	51.7179558085	28.4596332792
C	30.8988864522	50.7035945647	29.3823617677
C	29.9487825485	51.1164782869	30.3449198852
C	29.4832824132	50.5687934152	31.5819875300
N	31.0938293734	49.1048303463	32.7344429180
C	29.8647765337	49.2831256084	32.1702410134
C	29.1702171992	48.0464803355	32.2763941429
C	29.9733046559	47.1585303053	32.9203574321
C	31.2452137044	47.8276031558	33.2183924781
O	32.2509276040	47.4362684520	33.7629804670
N	25.9145772769	55.3472721831	31.7510453513
C	25.0616751809	55.1497821265	32.8080107759
C	24.3075548178	56.4307267779	33.0454752055
C	24.6683027244	57.3189924652	31.8248448960
C	25.8012072791	56.5512817815	31.1383261904
O	25.0091072356	54.1144018396	33.4077004503
C	26.5149202753	57.0086170887	30.0674249813
N	28.2196670247	55.2777468949	29.4126698923
C	27.6194213085	56.5024249239	29.3292731588
C	28.3415041813	57.2308068088	28.3293117588
C	29.3203312411	56.4237426349	27.8298952642
C	29.2235197339	55.1607982125	28.5006235497
H	26.2202173361	57.9851188404	29.7501643018
H	28.7980481114	51.1803860228	32.1371948458
H	30.6923179958	54.1474979240	27.4129602956
H	27.7708063704	54.4790346909	29.8452340326
H	26.4316576390	54.5481701504	31.4089737573
H	31.8433143407	49.7695401815	32.7490321328
H	28.6712284186	52.7451092132	30.3730087155
H	23.8407895705	57.3376030040	31.1201365761
H	24.6845826517	56.8289393064	33.9825360538

H	24.2505171137	51.8370976454	30.4066549077
H	31.5885247626	51.6846111826	27.5344774698
H	31.4376367828	49.7561982836	29.3983436063
H	28.1322773400	47.9103971278	31.9727014356
H	29.7616893743	46.1493481565	33.2736808171
H	23.2415792354	56.2673175695	33.2037804161
H	24.8866907632	58.3473018977	32.1128612619
H	28.1074482997	58.2476672801	28.0142249809
H	30.0453305574	56.6908882296	27.0610804140
49			
-100			
C	25.2756854972	51.4823602867	30.3027866436
H	25.5913295116	51.1339735921	31.2766700641
H	25.2358143818	50.6591641870	29.6045346099
C	26.2115057413	52.5605319644	29.7887668668
O	26.9930106453	53.0829708412	30.6572075533
O	26.1832220140	52.8960966397	28.6201456138
C	30.0169495099	54.0128699511	28.2293705182
N	29.5152885117	52.3509302730	29.9849086371
C	30.1276989436	52.7755508856	28.8441621509
C	31.0042971413	51.7129399572	28.4514930776
C	30.8841687522	50.6933994905	29.3715949910
C	29.9359636251	51.1065049499	30.3315371690
C	29.5042355732	50.5822458459	31.5937840100
N	31.0831755005	49.1135736325	32.7647655296
C	29.8627195686	49.2873024863	32.1828523249
C	29.1778652644	48.0461333741	32.2588004896
C	29.9778093830	47.1568024128	32.9099327589
C	31.2411822781	47.8293724081	33.2284733991
O	32.2473098495	47.4374511654	33.7719276139
N	25.9147560004	55.3481148742	31.7513200902
C	25.0615537897	55.1509768928	32.8079736089
C	24.3072978377	56.4312961089	33.0455184292
C	24.6683764814	57.3192868553	31.8249228524
C	25.8010707857	56.5513044874	31.1384611721
O	25.0096195527	54.1150863716	33.4077334312
C	26.5151687642	57.0080903950	30.0671904650
N	28.2194863485	55.2769147705	29.4129957339
C	27.6193717755	56.5014496299	29.3295049308
C	28.3424775201	57.2299555243	28.3293485733
C	29.3207928751	56.4229446263	27.8304049355
C	29.2230826411	55.1589188347	28.5006862900
H	26.2208144882	57.9845878684	29.7501990287
H	28.8802841411	51.2322610737	32.1750325000

H	30.6950974893	54.1481655286	27.4164370986
H	27.7699853800	54.4784563182	29.8454785329
H	26.4308695460	54.5485638584	31.4087455980
H	31.8323950414	49.7785885352	32.7809872514
H	28.6624153579	52.7387022834	30.3652174171
H	23.8410683846	57.3377431154	31.1201347311
H	24.6843861891	56.8292738048	33.9826125148
H	24.2503430135	51.8372020815	30.4068402023
H	31.5852224607	51.6822670895	27.5297273807
H	31.4289953364	49.7495976915	29.3932562223
H	28.1390490406	47.9099959453	31.9581436999
H	29.7641108336	46.1486106219	33.2648261149
H	23.2413388515	56.2676625812	33.2037036361
H	24.8868102001	58.3476217997	32.1128134638
H	28.1082680905	58.2467859128	28.0142788105
H	30.0455957418	56.6900458164	27.0613894545

50

AnPixJ-COOH_S0_MIN

C	25.1586163358	51.3865508483	30.3600744761
H	25.3964205828	51.0111432100	31.3449691896
H	25.1297365864	50.5812216610	29.6389048299
C	26.1972676865	52.3945565948	29.9782449283
O	26.9898962429	52.8835262292	30.7405718056
O	26.2035919651	52.7188424909	28.7027103364
H	26.9109979375	53.3310708239	28.5231523522
C	30.1670481200	54.1132972980	28.4648398787
N	29.7321199933	52.3017114354	30.1492673920
C	30.3558459520	52.8527573736	29.0435671488
C	31.2438835300	51.8601007358	28.5732233221
C	31.1494177714	50.7619716315	29.4072187814
C	30.1607557839	51.0506983115	30.3695718939
C	29.4006695334	50.1596017647	31.2604224225
N	31.1821379022	48.9002539019	32.3703709859
C	29.8605587949	49.1440550077	32.0199990321
C	29.0958749869	47.9671995908	32.5097026347
C	29.9487375350	47.1109871609	33.0611010946
C	31.2952095612	47.7507888669	33.1121318451
O	32.2848864736	47.3913048599	33.6787517283
N	25.9633206636	55.4519549499	31.8190600703
C	25.0633222410	55.2335350437	32.8316695257
C	24.3103503534	56.5189887826	33.0546439415
C	24.6814200536	57.4095081464	31.8338685887
C	25.8551846047	56.6696463208	31.1814568922
O	24.9844835626	54.1740082258	33.3879705363

C	26.5761537673	57.1111341428	30.1213272341
N	28.3081884515	55.3843793127	29.5686426923
C	27.7117791725	56.5709230736	29.4020805849
C	28.3875795864	57.2375010386	28.3408437034
C	29.3806785869	56.4124319321	27.8866543586
C	29.3549406545	55.2097073006	28.6517579269
H	26.2924626666	58.0761208358	29.7635417722
H	28.3353690093	50.2535693189	31.1551062581
H	30.8442375143	54.2586769103	27.6495857642
H	28.0446522718	54.7096879499	30.2488721520
H	26.5522953509	54.6945981556	31.5644549474
H	31.9105554338	49.5901822843	32.3915518855
H	28.9752794369	52.6988629293	30.6611248887
H	23.8658356708	57.4112534653	31.1150262847
H	24.6873405879	56.9182055793	33.9914033667
H	24.1513297521	51.7994897469	30.4141323676
H	31.7684195734	51.8687044625	27.6177888835
H	31.6984096967	49.8244092470	29.3197448278
H	28.0294191229	47.8069687340	32.3513832928
H	29.7965493319	46.0853974001	33.3973620719
H	23.2425307407	56.3677103324	33.2125779677
H	24.8830319112	58.4422367162	32.1182827433
H	28.1280061169	58.2214012131	27.9501659437
H	30.0796342051	56.6472477063	27.0839155173
50			
AnPixJ-COOH_S1_MIN			
C	25.1548837608	51.3583797444	30.3453973163
H	25.3868085237	50.9555073996	31.3204693747
H	25.1231899136	50.5727139172	29.6039521054
C	26.1985418168	52.3744445031	29.9994747318
O	26.9694821233	52.8523633375	30.7879009907
O	26.2193688847	52.7235498440	28.7293066912
H	26.9182959816	53.3520292525	28.5700341415
C	30.1201022896	54.0468975140	28.3418246603
N	29.7495823069	52.4424343279	30.1838064187
C	30.2875449739	52.8709682258	28.9920641789
C	31.1649882536	51.7740540977	28.5550094346
C	31.1077871167	50.7758337259	29.4482898172
C	30.1303776806	51.1471193663	30.4956771503
C	29.5803346565	50.4919611589	31.5453722994
N	31.2007463176	48.9701582562	32.5532158029
C	29.9820940739	49.1782690378	32.0637750444
C	29.1954800500	47.9743185640	32.2809013293
C	29.9688255703	47.0864807174	32.9303881372

C	31.3017869143	47.7033381727	33.1403397558
O	32.2835874199	47.2997928708	33.6788307038
N	25.9646364240	55.4392629675	31.8042385232
C	25.0742619038	55.2180690028	32.8171069228
C	24.3168414276	56.5008461017	33.0476897009
C	24.6840733265	57.4010579936	31.8331771249
C	25.8604807139	56.6707995169	31.1757972211
O	24.9965018565	54.1607797116	33.3836606349
C	26.5905834669	57.1225212262	30.1235906931
N	28.3065804403	55.3721614821	29.5023840479
C	27.7094662535	56.5935537227	29.3732751872
C	28.3767570132	57.2533534729	28.3310779433
C	29.3696498592	56.4096858823	27.8470146827
C	29.3252654288	55.2153260051	28.5810824333
H	26.3044584662	58.0956430620	29.7857812996
H	28.8026623045	50.9783914330	32.1012882747
H	30.7378567477	54.1506634235	27.4792010497
H	28.0636128780	54.6994934951	30.1903349709
H	26.5653528554	54.6910483704	31.5491329812
H	31.9582657042	49.6391803620	32.5430861244
H	28.9793436408	52.8577039317	30.6437723250
H	23.8666562535	57.4042355289	31.1165592848
H	24.6923233455	56.8954782053	33.9871740555
H	24.1517166448	51.7803216436	30.4061247606
H	31.6857851220	51.7448254284	27.5979392396
H	31.6500895010	49.8310863980	29.4105054046
H	28.1417210135	47.8734986537	32.0211046969
H	29.7512813387	46.0742859242	33.2712741286
H	23.2490805831	56.3459163831	33.2024495560
H	24.8813640011	58.4330857594	32.1231118272
H	28.1275789260	58.2431693792	27.9486621957
H	30.0711799402	56.6495964750	27.0480368956

AnPixJ-COOH_-145_-95_S1_SCAN_C14-C15-C16-C17

50

-145

C	25.1514734084	51.3616839268	30.3473448540
H	25.3862008876	50.9699024132	31.3265404686
H	25.1197260206	50.5665718950	29.6156079184
C	26.1920652591	52.3749176792	29.9846672376
O	26.9651789047	52.8704057817	30.7614476237
O	26.2107860828	52.7048426359	28.7099141696
H	26.9211348022	53.3159772548	28.5384620528
C	30.1285494805	54.0412599281	28.3687287938

N	29.7814942500	52.4356045952	30.2181071205
C	30.3180027946	52.8866471431	29.0249886797
C	31.2275336275	51.8016465568	28.5900091592
C	31.2042335944	50.8141635429	29.4933800192
C	30.1885021242	51.1490627835	30.5123875213
C	29.5158074521	50.3898439604	31.4318940042
N	31.2275584652	48.8626365955	32.3399450290
C	29.9352420077	49.1384257420	32.0119250767
C	29.1394044115	47.9944297453	32.3970525457
C	29.9499323173	47.0922819295	32.9752636935
C	31.3077900054	47.6753204256	33.0516832223
O	32.2905285481	47.2941895971	33.6150511521
N	25.9533563939	55.4473130502	31.8005503167
C	25.0638319185	55.2254469929	32.8209008375
C	24.3100580909	56.5089576577	33.0541209707
C	24.6756069177	57.4087383605	31.8390450614
C	25.8493456880	56.6760278687	31.1818728466
O	24.9910041806	54.1671875829	33.3817579720
C	26.5889755404	57.1330505714	30.1245399373
N	28.2941652083	55.3753341401	29.5091342096
C	27.6951605922	56.6058154066	29.3857422140
C	28.3748565274	57.2653795564	28.3408830403
C	29.3693620641	56.4224882310	27.8651219442
C	29.3124630871	55.2291645696	28.6029199751
H	26.3023038110	58.1075727173	29.7875652476
H	28.5480330722	50.7430221048	31.7341131667
H	30.7316423340	54.1424738277	27.4974193896
H	28.0419392373	54.6889204669	30.1823610459
H	26.5550631460	54.6972111328	31.5488808895
H	31.9588289378	49.5495303906	32.4096552014
H	28.9938497866	52.8235870907	30.6729465759
H	23.8578836415	57.4127254420	31.1218431837
H	24.6880761824	56.9029460002	33.9931196280
H	24.1476235957	51.7823358987	30.4056931494
H	31.7313113486	51.7717542691	27.6238924426
H	31.7500167128	49.8723599137	29.4368666310
H	28.0770440338	47.8754510246	32.1842109535
H	29.7493579656	46.0788848960	33.3229178800
H	23.2423707408	56.3554244619	33.2107681267
H	24.8757454004	58.4408760527	32.1266270903
H	28.1267711118	58.2524384744	27.9507095273
H	30.0738805813	56.6527950689	27.0659477012

50

-140

C	25.1574894469	51.3574293315	30.3451059535
H	25.3883535530	50.9484828964	31.3181154478
H	25.1267936538	50.5752880927	29.5997617399
C	26.1990463982	52.3764608645	30.0017609416
O	26.9736773526	52.8554850037	30.7869238741
O	26.2150799161	52.7332356818	28.7338087351
H	26.9162698345	53.3591844164	28.5779953335
C	30.1222876184	54.0371060610	28.3382287187
N	29.7990073737	52.4856499735	30.2357798850
C	30.3085738673	52.8918019021	29.0137402019
C	31.1818139369	51.7815147248	28.5770928318
C	31.1655205392	50.8163664918	29.5020679058
C	30.1959216024	51.1987516612	30.5511874421
C	29.5528627073	50.4641228407	31.5049751228
N	31.2560641688	48.9152452769	32.4197465389
C	29.9796705007	49.1922321236	32.0577505595
C	29.1749077838	48.0403756420	32.3877098983
C	29.9641315433	47.1218418792	32.9713563381
C	31.3208524230	47.7007704989	33.0982646973
O	32.2902168643	47.2966459554	33.6667277697
N	25.9690270494	55.4383944410	31.8020681100
C	25.0747689812	55.2155886251	32.8173241611
C	24.3173948478	56.4975581299	33.0466078055
C	24.6861962726	57.3971305733	31.8325745418
C	25.8631873704	56.6655550322	31.1785730143
O	24.9992116955	54.1574085416	33.3783238421
C	26.5977364850	57.1201600701	30.1194687989
N	28.3070847540	55.3679597017	29.4959468461
C	27.7034136683	56.5946089136	29.3744759147
C	28.3747653079	57.2566696721	28.3270097697
C	29.3696926134	56.4187155130	27.8449644860
C	29.3218900869	55.2244102893	28.5815713750
H	26.3087010998	58.0934491125	29.7825548608
H	28.6060857189	50.8329663770	31.8513229271
H	30.7143835488	54.1265916418	27.4567684474
H	28.0779981644	54.6907155681	30.1859659137
H	26.5714026479	54.6887251210	31.5509155910
H	31.9920742117	49.5970444021	32.5079461145
H	28.9929601820	52.8668362496	30.6648378377
H	23.8711771813	57.3999325660	31.1125645136
H	24.6906946938	56.8928352058	33.9868789021
H	24.1540183207	51.7785706533	30.4063655428
H	31.6782404969	51.7374943570	27.6077167803
H	31.6562937457	49.8455298919	29.4336207913

H	28.1425026853	47.9155942545	32.0611576521
H	29.7487945122	46.0988920095	33.2800156626
H	23.2496402067	56.3409608435	33.1997233430
H	24.8825612531	58.4293190330	32.1225656271
H	28.1254375006	58.2468731066	27.9456964415
H	30.0709887330	56.6540980664	27.0444359996
50			
-135			
C	25.1576851496	51.3574704855	30.3450106650
H	25.3885160472	50.9480530623	31.3178168592
H	25.1271565745	50.5759070900	29.5991873601
C	26.1995916866	52.3766657571	30.0024248880
O	26.9736158185	52.8545668265	30.7878818068
O	26.2156061876	52.7335833107	28.7342397687
H	26.9157938592	53.3609758536	28.5792828867
C	30.1248854109	54.0417085938	28.3388233413
N	29.7902631126	52.4757774884	30.2209497384
C	30.3109830474	52.8916672911	29.0106913340
C	31.1792905636	51.7810881077	28.5702380404
C	31.1481733572	50.8058714940	29.4847196734
C	30.1811044635	51.1860758727	30.5352559099
C	29.5634752254	50.4775380090	31.5206116578
N	31.2416455513	48.9349254549	32.4652812983
C	29.9793574227	49.1953944959	32.0688890205
C	29.1810758053	48.0286246282	32.3598115837
C	29.9664690095	47.1122960349	32.9534211615
C	31.3165542490	47.7004043152	33.1077252054
O	32.2901752463	47.2965491045	33.6692043647
N	25.9692507139	55.4381561111	31.8024752969
C	25.0752319928	55.2148580138	32.8169260384
C	24.3176020807	56.4971186349	33.0461289021
C	24.6865030297	57.3968593411	31.8320865975
C	25.8637110260	56.6657162404	31.1781259623
O	24.9993297634	54.1571085696	33.3783008297
C	26.5973791741	57.1195037897	30.1199303990
N	28.3075251455	55.3679154456	29.4950812589
C	27.7048708228	56.5936850254	29.3736642070
C	28.3751220759	57.2560735372	28.3266156630
C	29.3687521091	56.4175644631	27.8430817568
C	29.3224385272	55.2225339434	28.5790269077
H	26.3088497731	58.0925689281	29.7827015068
H	28.6416010984	50.8674652682	31.9059987476
H	30.7136926057	54.1282906570	27.4555997627
H	28.0785391857	54.6914515584	30.1855802593

H	26.5717130198	54.6890044023	31.5507707775
H	31.9913447803	49.6056682258	32.5123625548
H	28.9956516435	52.8663840331	30.6610573932
H	23.8712796144	57.3995238822	31.1124550633
H	24.6907708296	56.8923794534	33.9864939509
H	24.1541863140	51.7785292951	30.4063834377
H	31.6791920843	51.7386986181	27.6025766057
H	31.6495066578	49.8397779872	29.4264503921
H	28.1453962865	47.9093272086	32.0416872189
H	29.7493857844	46.0931744689	33.2733167044
H	23.2498772967	56.3404013119	33.1993298079
H	24.8827148924	58.4289893960	32.1223890369
H	28.1255356813	58.2464405256	27.9458967231
H	30.0706975504	56.6536576471	27.0433320102
50			
-130			
C	25.1583801602	51.3564655490	30.3440480532
H	25.3896044612	50.9469072231	31.3166037039
H	25.1274599714	50.5753327576	29.5977694332
C	26.1997148838	52.3770810546	30.0040707437
O	26.9707277810	52.8545902891	30.7928167887
O	26.2173168253	52.7359207905	28.7364480198
H	26.9149978767	53.3668244995	28.5829273819
C	30.1255854395	54.0427005102	28.3324985410
N	29.7748938962	52.4745834667	30.2055436549
C	30.2962416311	52.8824246927	28.9945299383
C	31.1601737924	51.7679980350	28.5569154646
C	31.1233963615	50.7943636766	29.4738137416
C	30.1670643748	51.1877234421	30.5313170757
C	29.5853201532	50.5093685320	31.5561094537
N	31.2415501720	48.9432315481	32.4815983704
C	29.9867844100	49.2075334737	32.0783309745
C	29.1848042051	48.0301426830	32.3371184713
C	29.9628237105	47.1161855674	32.9427987098
C	31.3117155719	47.7020097333	33.1151140573
O	32.2771457815	47.2935230982	33.6833568240
N	25.9711249707	55.4369671609	31.8028903200
C	25.0768639367	55.2136910295	32.8153144795
C	24.3181473141	56.4950205042	33.0448692136
C	24.6871056118	57.3955347207	31.8314130271
C	25.8647625285	56.6652801258	31.1765076800
O	25.0001864226	54.1555369838	33.3780807037
C	26.5964630050	57.1185869389	30.1198950205
N	28.3080949062	55.3672700617	29.4941987918

C	27.7076957655	56.5927168777	29.3724624537
C	28.3758016706	57.2546569312	28.3264637766
C	29.3675697520	56.4142090141	27.8404430404
C	29.3243037719	55.2192069367	28.5756347932
H	26.3088815297	58.0920214440	29.7832324742
H	28.7043278898	50.9371306195	31.9970154562
H	30.7303501920	54.1382474933	27.4604863123
H	28.0761453237	54.6942501540	30.1866942191
H	26.5737740694	54.6884657767	31.5508753322
H	31.9812041579	49.6244114215	32.5620755083
H	28.9918126460	52.8807249446	30.6523355566
H	23.8719544279	57.3982381820	31.1120126396
H	24.6915820159	56.8895895474	33.9853344509
H	24.1551821061	51.7781250498	30.4062089819
H	31.6715889247	51.7321845811	27.5950220140
H	31.6282765113	49.8299528214	29.4183629703
H	28.1506647028	47.9067511730	32.0155643135
H	29.7486009276	46.0966447799	33.2632862028
H	23.2504378668	56.3381426451	33.1980126917
H	24.8827660417	58.4276449907	32.1221576539
H	28.1258800579	58.2450806461	27.9461124866
H	30.0688254871	56.6520599016	27.0406090572
50			
-125			
C	25.1587678678	51.3564395482	30.3437407595
H	25.3904387019	50.9473153801	31.3162766487
H	25.1277014647	50.5753564670	29.5975287945
C	26.1997850106	52.3771660564	30.0037129683
O	26.9706993356	52.8531539621	30.7933416824
O	26.2172311741	52.7359857348	28.7361645682
H	26.9141684941	53.3681265327	28.5832456568
C	30.1240778928	54.0456888445	28.3306613664
N	29.7647575410	52.4641611927	30.1908347255
C	30.2982816871	52.8808542812	28.9914403090
C	31.1598971445	51.7690049851	28.5517487012
C	31.1036489911	50.7831008347	29.4561246155
C	30.1508146597	51.1745114618	30.5171238156
C	29.5991133286	50.5219019870	31.5711695721
N	31.2265366659	48.9600826614	32.5207798942
C	29.9862353999	49.2069509349	32.0870521039
C	29.1896457884	48.0161070949	32.3085761970
C	29.9648843077	47.1047780025	32.9239193764
C	31.3050035937	47.7024576547	33.1261602089
O	32.2736552491	47.2906448036	33.6839987902

N	25.9711236284	55.4367577752	31.8037255529
C	25.0772681884	55.2126839792	32.8146020066
C	24.3181095339	56.4941052238	33.0441805962
C	24.6866955953	57.3953028744	31.8308562014
C	25.8660318631	56.6666350576	31.1763448917
O	25.0001748064	54.1545776131	33.3787258554
C	26.5966192635	57.1195931264	30.1220788303
N	28.3089769395	55.3688559877	29.4934747623
C	27.7076304826	56.5908765840	29.3679830491
C	28.3752292969	57.2529488027	28.3243804277
C	29.3701080732	56.4134617538	27.8411211576
C	29.3278486434	55.2181231716	28.5750203564
H	26.3077594715	58.0923390223	29.7846383397
H	28.7511273986	50.9711464984	32.0524980105
H	30.7311387462	54.1433538459	27.4605361851
H	28.0749209494	54.6974082079	30.1859519313
H	26.5743752366	54.6892677342	31.5508936736
H	31.9740410615	49.6353379711	32.5685439013
H	28.9961688641	52.8835755272	30.6478569420
H	23.8708269185	57.3978070324	31.1120149683
H	24.6917137460	56.8884190996	33.9846652926
H	24.1555897192	51.7781121552	30.4061335953
H	31.6737284579	51.7341792211	27.5911074576
H	31.6192877307	49.8238902022	29.4103130000
H	28.1518425491	47.8985438943	31.9967904023
H	29.7480400133	46.0897077979	33.2566025338
H	23.2504287374	56.3373251272	33.1976236456
H	24.8825633752	58.4272809082	32.1219304224
H	28.1259694652	58.2445085686	27.9465632520
H	30.0693985481	56.6518056349	27.0397148452
50			
-120			
C	25.1618244633	51.3518764645	30.3352873028
H	25.3893230753	50.9179681305	31.2979520173
H	25.1340948198	50.5902225576	29.5693304685
C	26.2020379799	52.3833871304	30.0252386655
O	26.9772202771	52.8313602317	30.8261781351
O	26.2095919325	52.7874374968	28.7704328012
H	26.8898546725	53.4431355004	28.6401156849
C	30.1051886884	54.0309826492	28.2970815715
N	29.7608092701	52.4665483081	30.1705285824
C	30.2651200742	52.8592797264	28.9519626532
C	31.0932964887	51.7303554582	28.4986322314
C	31.0623226795	50.7594813328	29.4212398003

C	30.1659199011	51.1880929045	30.5119106108
C	29.6689666699	50.5603858809	31.6042004031
N	31.2869846273	49.0026155172	32.6260112990
C	30.0783200620	49.2504661849	32.1219185891
C	29.2673236896	48.0637642193	32.2963705583
C	29.9889428815	47.1513309803	32.9659900506
C	31.3184137471	47.7439923298	33.2463310507
O	32.2516452967	47.3334629412	33.8632867215
N	25.9732209829	55.4264167791	31.8020535516
C	25.0848324969	55.2051071201	32.8164535090
C	24.3195800000	56.4832240486	33.0431499228
C	24.6872768528	57.3852270095	31.8301058178
C	25.8641830942	56.6556455851	31.1707738537
O	25.0126249317	54.1494420364	33.3858305063
C	26.5900400790	57.1048150478	30.1148996125
N	28.3001301374	55.3500418291	29.4760606111
C	27.7063276181	56.5767444105	29.3602434828
C	28.3720835080	57.2420331692	28.3213909983
C	29.3636970359	56.4020520101	27.8283566902
C	29.3213013708	55.2033590499	28.5530839540
H	26.3008982501	58.0763678662	29.7746683097
H	28.8289088517	51.0078662527	32.1030293908
H	30.7030657574	54.1232042332	27.4186475503
H	28.0946530403	54.6955125904	30.1932199467
H	26.5752847932	54.6784404141	31.5485297263
H	32.0405271309	49.6697959583	32.7162828528
H	28.9970031358	52.8871335403	30.6367196171
H	23.8702965743	57.3907088500	31.1131453193
H	24.6856688987	56.8801369064	33.9854482893
H	24.1586337899	51.7727584825	30.4026211790
H	31.6048745567	51.6956113637	27.5367862045
H	31.5125033732	49.7692812434	29.3511814140
H	28.2716956861	47.9627273406	31.8644174599
H	29.7364306003	46.1311637142	33.2550633085
H	23.2522041213	56.3193028513	33.1911554160
H	24.8832415700	58.4163597594	32.1240958399
H	28.1245763887	58.2362531541	27.9494583445
H	30.0676587561	56.6493776351	27.0337907816
50			
-115			
C	25.1618916875	51.3522331348	30.3351217716
H	25.3888842116	50.9172542445	31.2973927523
H	25.1349765044	50.5916995121	29.5682079142
C	26.2017719257	52.3837749719	30.0258596617

O	26.9774619232	52.8305694007	30.8275585517
O	26.2097831167	52.7882366799	28.7711799140
H	26.8884186975	53.4457667938	28.6418293499
C	30.1078161854	54.0358568277	28.2983360734
N	29.7494677116	52.4564365348	30.1571450399
C	30.2713283620	52.8598630219	28.9525969556
C	31.0888093490	51.7313093828	28.4917413031
C	31.0369104011	50.7470210115	29.4007675011
C	30.1472556310	51.1742195746	30.4933264702
C	29.6856116987	50.5680501489	31.6114394877
N	31.2676542508	49.0231879938	32.6761932359
C	30.0797067525	49.2519597760	32.1314865610
C	29.2768309209	48.0535430839	32.2721843420
C	29.9937176510	47.1409114801	32.9491862775
C	31.3133989919	47.7454245573	33.2575323557
O	32.2485053246	47.3382297879	33.8696537885
N	25.9726596485	55.4258511288	31.8023315324
C	25.0851729456	55.2041941699	32.8166733435
C	24.3196023979	56.4827780531	33.0431378479
C	24.6871830142	57.3850684756	31.8300506131
C	25.8639220229	56.6558839748	31.1701744585
O	25.0127830912	54.1491097552	33.3866591090
C	26.5887234312	57.1043499570	30.1152438477
N	28.2987752224	55.3491019456	29.4743829775
C	27.7080802731	56.5757253851	29.3594830875
C	28.3725288849	57.2414247095	28.3226423887
C	29.3626809517	56.3999563380	27.8268248057
C	29.3205457900	55.2012364658	28.5491658655
H	26.3005674209	58.0757605219	29.7743709851
H	28.8781306849	51.0306503343	32.1435257846
H	30.7011502067	54.1221685767	27.4159893765
H	28.0946602530	54.6962257344	30.1932120864
H	26.5748598068	54.6784276292	31.5481507979
H	32.0399043212	49.6733664739	32.7237486714
H	28.9968290703	52.8868129117	30.6324973498
H	23.8699983923	57.3906462707	31.1135024881
H	24.6852669200	56.8798205590	33.9855284547
H	24.1585617179	51.7727810713	30.4024675693
H	31.6049332127	51.6967091734	27.5323216690
H	31.5031942128	49.7634743458	29.3434751403
H	28.2776536403	47.9593623373	31.8469293593
H	29.7372529974	46.1248720198	33.2491141820
H	23.2522354173	56.3186323671	33.1909585966
H	24.8832162338	58.4161176352	32.1242880318

H	28.1247937037	58.2354358994	27.9503037180
H	30.0676578072	56.6485394580	27.0335523954
50			
-110			
C	25.1622201598	51.3518894764	30.3323255639
H	25.3908665019	50.9177026897	31.2945903964
H	25.1339522451	50.5907503640	29.5657056442
C	26.2011990484	52.3858523266	30.0268108575
O	26.9724911178	52.8311405174	30.8333642088
O	26.2127263950	52.7948596594	28.7734509055
H	26.8867529552	53.4586707328	28.6498680863
C	30.1013631100	54.0317971075	28.2911995898
N	29.7246320388	52.4399384511	30.1326915941
C	30.2479663360	52.8412859377	28.9305880172
C	31.0646723336	51.7154491607	28.4718235577
C	31.0128070716	50.7317768231	29.3852361938
C	30.1375030186	51.1688657488	30.4837401996
C	29.7182221304	50.5941339169	31.6368785560
N	31.2755450789	49.0334351244	32.7127605355
C	30.1009050305	49.2677237498	32.1466462588
C	29.2955021005	48.0619553282	32.2562633362
C	29.9941944870	47.1498650973	32.9513028296
C	31.3092844547	47.7518366257	33.2894579548
O	32.2313263530	47.3366152782	33.9125167148
N	25.9724914410	55.4243654919	31.8024924022
C	25.0870087054	55.2042335332	32.8174071087
C	24.3201177146	56.4815804477	33.0437965967
C	24.6876265843	57.3841252072	31.8306992747
C	25.8637400171	56.6547842639	31.1695049237
O	25.0159407844	54.1489713614	33.3897796865
C	26.5866423237	57.1027621069	30.1144572293
N	28.2940778448	55.3456630279	29.4704736278
C	27.7065884905	56.5725628230	29.3553266653
C	28.3722819104	57.2386543345	28.3226532699
C	29.3649050289	56.3967397544	27.8283429448
C	29.3211509685	55.1973686058	28.5476890941
H	26.2996467351	58.0751012325	29.7761269898
H	28.9561955959	51.0949060234	32.2029523320
H	30.7128976823	54.1296546474	27.4219356663
H	28.0896381410	54.6974827087	30.1934836018
H	26.5708046456	54.6753365414	31.5450198189
H	32.0352213986	49.6939372009	32.7988219957
H	28.9896777047	52.8841208244	30.6237945113
H	23.8698312676	57.3898635401	31.1148432451

H	24.6846703726	56.8787750287	33.9864332888
H	24.1589211269	51.7722806697	30.4010958075
H	31.5915002763	51.6889815524	27.5179793169
H	31.4769506916	49.7473428922	29.3258426997
H	28.2999880360	47.9668771641	31.8226979531
H	29.7346720058	46.1319758467	33.2421921670
H	23.2527140787	56.3164382730	33.1902363164
H	24.8836273803	58.4150447219	32.1254121752
H	28.1246243741	58.2330464157	27.9512812396
H	30.0686607620	56.6478786371	27.0347913201
50			
-105			
C	25.1620782652	51.3518522914	30.3316500172
H	25.3915929803	50.9176617499	31.2926031265
H	25.1327358873	50.5898091234	29.5671604325
C	26.2014836365	52.3854659911	30.0267731788
O	26.9716905225	52.8307610332	30.8341762821
O	26.2138410405	52.7972593383	28.7735815575
H	26.8875156013	53.4624485672	28.6500204918
C	30.1055205713	54.0345549805	28.2924640941
N	29.7134015093	52.4286500563	30.1215139563
C	30.2490683652	52.8396834652	28.9276313421
C	31.0607026759	51.7159933316	28.4672479707
C	30.9955096764	50.7234624962	29.3726793633
C	30.1167131337	51.1549781929	30.4648065051
C	29.7327404365	50.5970303931	31.6415213254
N	31.2549127396	49.0512162192	32.7600750188
C	30.0978014217	49.2723334295	32.1564064152
C	29.3052887300	48.0525042694	32.2346523702
C	29.9996781476	47.1433238102	32.9360882995
C	31.3029898584	47.7553023560	33.3063084590
O	32.2301516165	47.3351229712	33.9162166157
N	25.9733667735	55.4234825919	31.8025151455
C	25.0876681073	55.2047302335	32.8166918943
C	24.3200469009	56.4810350551	33.0439393333
C	24.6876627984	57.3833108302	31.8314077722
C	25.8630450187	56.6551180432	31.1692213898
O	25.0158098045	54.1483234000	33.3918648973
C	26.5853399157	57.1035326149	30.1154507332
N	28.2919052546	55.3449171246	29.4681491374
C	27.7065736955	56.5727818509	29.3550108679
C	28.3714503555	57.2382357912	28.3238361827
C	29.3641887582	56.3951684227	27.8277445569
C	29.3218759238	55.1972470085	28.5461971271

H	26.2997753501	58.0755663810	29.7762137791
H	29.0118930182	51.1201489667	32.2374739590
H	30.7121419220	54.1303164495	27.4190189237
H	28.0881276370	54.6987925343	30.1926846709
H	26.5705832538	54.6742698815	31.5436917936
H	32.0295750069	49.6974359409	32.8033214329
H	28.9894491916	52.8829196529	30.6198795556
H	23.8705403804	57.3897518253	31.1157316094
H	24.6846788876	56.8793140494	33.9860712532
H	24.1587609889	51.7721315952	30.4008367295
H	31.5908216311	51.6901447821	27.5152118384
H	31.4689918074	49.7431119469	29.3198161093
H	28.3064904843	47.9622984573	31.8076488253
H	29.7358392583	46.1290786440	33.2356927636
H	23.2526006518	56.3159752379	33.1901611288
H	24.8838209563	58.4142083807	32.1260928054
H	28.1242937912	58.2325725112	27.9519824866
H	30.0685778304	56.6472251942	27.0350463354
50			
-100			
C	25.1632823080	51.3533643285	30.3329770809
H	25.3945244893	50.9148645939	31.2936336076
H	25.1419293161	50.5993991287	29.5583955397
C	26.1984451184	52.3900367560	30.0288702478
O	26.9697703277	52.8385631622	30.8334634265
O	26.2107832055	52.7942938542	28.7753069438
H	26.8805433056	53.4630499880	28.6593590591
C	30.0998611955	54.0297925031	28.2888955539
N	29.6924987725	52.4144946721	30.1036007463
C	30.2336095556	52.8282244321	28.9138753447
C	31.0435590373	51.7055204451	28.4514503295
C	30.9610549954	50.7050900014	29.3492140358
C	30.0999752040	51.1473859461	30.4484397812
C	29.7627756772	50.6121623065	31.6532742874
N	31.2537135394	49.0607394198	32.7944124471
C	30.1096503187	49.2849921645	32.1692973384
C	29.3158202259	48.0573589801	32.2228956997
C	29.9996505896	47.1472643795	32.9322617388
C	31.2935848869	47.7641328986	33.3291188274
O	32.2172311358	47.3372335916	33.9390894061
N	25.9665602381	55.4213486479	31.8008624506
C	25.0880728789	55.2024890696	32.8196496062
C	24.3208788726	56.4788642365	33.0476443684
C	24.6865341583	57.3838081752	31.8345116498

C	25.8603472027	56.6536823153	31.1686161792
O	25.0223790860	54.1485259272	33.3967954149
C	26.5834649854	57.1000524168	30.1148690077
N	28.2891869604	55.3415913483	29.4629511205
C	27.7049481808	56.5685556567	29.3494208861
C	28.3699903391	57.2358507622	28.3212616351
C	29.3653251505	56.3932743174	27.8266374129
C	29.3200289253	55.1950668075	28.5422430494
H	26.3024339744	58.0761996892	29.7820933270
H	29.0966807896	51.1722050129	32.2818634829
H	30.7233708817	54.1324960183	27.4277230907
H	28.0883247786	54.6981855084	30.1904019828
H	26.5666352552	54.6745037492	31.5440328681
H	32.0271035683	49.7084406117	32.8699838520
H	28.9813013702	52.8782507815	30.6118870540
H	23.8657254757	57.3897614394	31.1217290977
H	24.6837239813	56.8727609889	33.9923971635
H	24.1593961623	51.7724697860	30.4010231922
H	31.5838220571	51.6861832974	27.5049784623
H	31.4460063460	49.7299497721	29.3045959660
H	28.3165737300	47.9691240684	31.7965295516
H	29.7347910221	46.1328149838	33.2302699341
H	23.2529651014	56.3138899853	33.1905104777
H	24.8831772096	58.4147234126	32.1288113867
H	28.1229291705	58.2309412809	27.9513661229
H	30.0699672535	56.6474398248	27.0348379100
50			
-95			
C	25.1635738157	51.3547131347	30.3321831626
H	25.3923913320	50.9153250839	31.2898569487
H	25.1438210045	50.6029207005	29.5580821744
C	26.2003462289	52.3900789105	30.0332612669
O	26.9700923207	52.8399129606	30.8376291581
O	26.2108181421	52.7933795195	28.7782710281
H	26.8771976277	53.4648372225	28.6541291316
C	30.0994228551	54.0286285511	28.2892593202
N	29.6753550932	52.3997797174	30.0901858491
C	30.2259176315	52.8219268711	28.9055516335
C	31.0326237074	51.7014051650	28.4434318188
C	30.9408356852	50.6950966440	29.3368688070
C	30.0834844791	51.1368107171	30.4347802630
C	29.7858440254	50.6204594734	31.6635055572
N	31.2381237164	49.0742706853	32.8304517036
C	30.1103860890	49.2946376614	32.1789373102

C	29.3228936862	48.0535220745	32.2079433340
C	29.9990528977	47.1457340255	32.9250635211
C	31.2889355446	47.7660116592	33.3405258914
O	32.2138084012	47.3379406700	33.9445927039
N	25.9639862029	55.4208242816	31.7985932970
C	25.0881439087	55.2026747217	32.8189437817
C	24.3203265264	56.4791281796	33.0489054952
C	24.6863123587	57.3836769389	31.8362072473
C	25.8582053330	56.6537547361	31.1678961540
O	25.0247306273	54.1493643649	33.3973820865
C	26.5824738439	57.0979293359	30.1162183557
N	28.2880418607	55.3414319180	29.4595007179
C	27.7046004867	56.5686016739	29.3478855438
C	28.3680448385	57.2353240587	28.3213301919
C	29.3657413949	56.3917473820	27.8257885525
C	29.3217128151	55.1960770039	28.5407274696
H	26.3033450792	58.0729038678	29.7855727028
H	29.1739787746	51.2097729569	32.3158590850
H	30.7274111644	54.1341344732	27.4326346474
H	28.0895352268	54.6999354213	30.1888005948
H	26.5665981635	54.6749856654	31.5443137666
H	32.0147301053	49.7190326751	32.8980161739
H	28.9792380586	52.8758501716	30.6086903596
H	23.8650038007	57.3899000417	31.1255443899
H	24.6827872607	56.8714009839	33.9945741236
H	24.1594928749	51.7732749399	30.4006998099
H	31.5809003956	51.6858956429	27.5015086907
H	31.4339844763	49.7238712993	29.2969607756
H	28.3225668110	47.9654336177	31.7840876770
H	29.7347524943	46.1318251255	33.2253984851
H	23.2524245673	56.3132498166	33.1908097789
H	24.8830266985	58.4146144067	32.1303814428
H	28.1218953581	58.2306141060	27.9513636980
H	30.0701603529	56.6468171959	27.0340813004

54

Cph1-QM_S0_MIN

C	36.3459791445	57.5914822286	59.3548133241
H	35.8179971451	58.5177741119	59.5196583993
C	37.4349850553	57.4272504255	60.3854290061
O	37.3772557390	58.0049835811	61.4668852520
N	38.4272537105	56.6037466656	60.0628741862
H	38.3765471047	56.1489081983	59.1785822902
C	39.5122791848	56.2132347779	60.9455886449
H	39.5772367499	56.9136130822	61.7614939641

C	39.0452381757	58.2997523186	65.2421638696
N	36.9844161651	57.2016970866	64.2941746346
C	37.9564758700	57.4016548667	65.2534768604
C	37.6271360095	56.5719349680	66.3214169364
C	36.4661749123	55.8826822093	66.0009138978
C	36.0879657456	56.2925049552	64.7248909298
C	34.9815953855	55.8704496734	63.8716189095
N	33.4269267660	54.9795082212	65.5670518364
C	33.8329522981	55.2871648712	64.2778141294
C	32.7482723013	54.8016632521	63.4023484787
C	31.7853327933	54.2747194000	64.1424283416
C	32.1591383647	54.4144386042	65.5779843701
O	31.5346350180	54.1333554626	66.5468357497
N	36.7652283863	61.0428477733	61.2603645819
C	35.7087422937	61.3469870784	60.4317594113
C	36.1229620121	62.5252190695	59.5744756183
C	37.5767745832	62.8118234392	59.9834777377
C	37.8839541763	61.8119087190	61.0820186703
O	34.6862683912	60.7450958191	60.4200218588
C	39.0622118509	61.7573304732	61.7372483635
N	38.8443754043	59.7908062128	63.2387991849
C	39.4377838239	60.9184808956	62.8483828864
C	40.5000759468	61.2047547205	63.7405981689
C	40.5113107734	60.2237291736	64.6822727083
C	39.4573289251	59.3094930127	64.4018511528
H	39.7813746734	62.5080980230	61.4781165926
H	35.0842477147	56.0626007721	62.8196724606
H	39.6670610950	58.1983653497	66.1132726845
H	38.2852299605	59.2204027503	62.6329419228
H	36.6946120605	60.2628615213	61.8718982682
H	33.7910385397	55.4046503933	66.3883345804
H	37.0094463246	57.5468157503	63.3561885026
H	38.2686614220	62.6644910671	59.1645578141
H	35.4516913438	63.3536221811	59.7633952209
H	38.1798964667	56.4944331963	67.2361809536
H	35.9843973599	55.1388524608	66.5996028488
H	32.7875675627	54.8673635037	62.3334717709
H	30.8641816783	53.8264080167	63.8319392113
H	36.0165538075	62.2518663504	58.5322508154
H	37.7140760925	63.8194309239	60.3505869554
H	41.1331230776	62.0654580968	63.6775254410
H	41.1699363602	60.1494806398	65.5246845607
H	36.7349896819	57.5587266418	58.3442156335
H	35.6372813767	56.7771184045	59.4694427084

H	39.3456804100	55.2200943512	61.3448509972
H	40.4449374214	56.2232854049	60.3982514675

54

Cph1-QM_S1_MIN

C	36.2947983214	57.5808224564	59.4016663928
H	35.7278831914	58.4739120931	59.6169548702
C	37.4033247491	57.4171254916	60.4119281701
O	37.3621765649	57.9987938373	61.4900839616
N	38.3984226275	56.6021069884	60.0797501889
H	38.3455995790	56.1557036220	59.1926293237
C	39.5133794697	56.2424662530	60.9398362612
H	39.6212262752	56.9818531150	61.7157046175
C	39.1273811034	58.1238173770	65.2353984755
N	36.9789379010	57.3647828393	64.2941853897
C	38.0312836390	57.3534913838	65.1937176003
C	37.6479001077	56.3695515569	66.2161028997
C	36.4437934422	55.8820826890	65.9281378657
C	35.9846376423	56.4906969299	64.6726301732
C	34.8463941478	56.2939940419	63.9417096632
N	33.2732217574	55.2760044139	65.5842661791
C	33.7338412362	55.4546412912	64.3178974313
C	32.8974674700	54.6623977366	63.4494607779
C	31.9708849945	54.0336638099	64.1832191454
C	32.1592577090	54.4251228076	65.5985337298
O	31.5482865625	54.1260650162	66.5710764783
N	36.7899666089	60.9418576069	61.1235231653
C	35.7477354484	61.2949410690	60.3040782002
C	36.1371385393	62.5617585586	59.5768368324
C	37.5950465650	62.8194528114	59.9900275359
C	37.8968925985	61.7612924338	61.0432031245
O	34.7363070905	60.6719524992	60.2043030077
C	39.0459337121	61.7173077499	61.7704524448
N	38.8770500784	59.7216844601	63.2927323760
C	39.4464293036	60.8894463493	62.8708428956
C	40.4726978830	61.1928882645	63.7792902082
C	40.4926802229	60.2062810009	64.7434667606
C	39.4905316538	59.2789034120	64.4288075978
H	39.7493064657	62.5004966791	61.5553410068
H	34.7681444334	56.7954868473	62.9956303662
H	39.7984974963	57.9393075022	66.0471292819
H	38.2862277604	59.1584141405	62.7194699764
H	36.7353907094	60.0796844881	61.6203668031
H	33.5166700575	55.8325416773	66.3748150147
H	37.0359059987	57.6898273395	63.3577532923

H	38.2705193014	62.7099940733	59.1501598882
H	35.4638836249	63.3569923640	59.8731284660
H	38.2541526026	56.1338672009	67.0672517006
H	35.8924232359	55.1575758077	66.4894477291
H	33.0443719405	54.5880655062	62.3915975974
H	31.2113916800	53.3485621510	63.8661834326
H	36.0073466170	62.4077396283	58.5132109506
H	37.7373349553	63.8126313501	60.3940787343
H	41.0927667814	62.0656114069	63.7347098771
H	41.1236889315	60.1573122136	65.6056865145
H	36.6774772697	57.6202199054	58.3894475800
H	35.6269827630	56.7282702563	59.4769108404
H	39.3575578016	55.2709288942	61.3940485685
H	40.4202460141	56.2150297488	60.3522743998

Cph1-QM_-145_-95_S1_SCAN_C14-C15-
C16-C17
54
-145

C	36.2946575167	57.5811352699	59.4018581590
H	35.7277337212	58.4743203974	59.6170091394
C	37.4033081829	57.4159216399	60.4099063271
O	37.3624304059	57.9992908204	61.4907686871
N	38.3988549606	56.6022455209	60.0804621368
H	38.3453869056	56.1562440509	59.1930640360
C	39.5135751998	56.2422813951	60.9400257508
H	39.6212204024	56.9817970889	61.7157332060
C	39.1276015142	58.1239274565	65.2355429331
N	36.9792731197	57.3647070984	64.2940885700
C	38.0316250659	57.3529298837	65.1937959142
C	37.6481377335	56.3695938831	66.2161905263
C	36.4433721767	55.8823896910	65.9282177163
C	35.9852082489	56.4894768060	64.6719347025
C	34.8464876728	56.2934557102	63.9413945149
N	33.2721235299	55.2776920663	65.5843020648
C	33.7341217372	55.4543269016	64.3180680646
C	32.8980245530	54.6618624881	63.4494193855
C	31.9703621955	54.0333708224	64.1830350150
C	32.1586589329	54.4251227002	65.5986010415
O	31.5492307989	54.1261008798	66.5700624565
N	36.7899442165	60.9417795114	61.1235711515
C	35.7471153207	61.2944919683	60.3037278826
C	36.1371751241	62.5619676189	59.5765884749
C	37.5947728189	62.8193269972	59.9900088671
C	37.8968973558	61.7611514861	61.0431649825

O	34.7375002675	60.6727761919	60.2047952040
C	39.0456105486	61.7171145319	61.7701036088
N	38.8772286050	59.7217293265	63.2927789364
C	39.4469027349	60.8894104135	62.8704966938
C	40.4725668946	61.1929072846	63.7791123606
C	40.4925800768	60.2061942981	64.7435699133
C	39.4910331740	59.2785770437	64.4286899717
H	39.7490068986	62.5000394998	61.5554605077
H	34.7677420954	56.7963277229	62.9962051989
H	39.7983964721	57.9396081761	66.0469634452
H	38.2863316016	59.1589990347	62.7202473035
H	36.7352071419	60.0798754966	61.6204328780
H	33.5167973066	55.8325838731	66.3750144138
H	37.0358949234	57.6899920203	63.3582470093
H	38.2701665385	62.7100528903	59.1502534520
H	35.4638202353	63.3570032030	59.8730276868
H	38.2540328484	56.1338385105	67.0671871824
H	35.8925887867	55.1578011658	66.4893162012
H	33.0440214404	54.5878298908	62.3919319094
H	31.2111643353	53.3483342493	63.8661763005
H	36.0072423488	62.4074151479	58.5131709315
H	37.7370759161	63.8124192185	60.3939270771
H	41.0927142816	62.0653414788	63.7347763083
H	41.1234652844	60.1575427466	65.6056114880
H	36.6775957531	57.6203814201	58.3897230117
H	35.6270382865	56.7285271610	59.4770731132
H	39.3577086432	55.2708130960	61.3940186572
H	40.4200617864	56.2150759017	60.3522432942
54			
-140			
C	36.2941699572	57.5815542932	59.4030930107
H	35.7256871465	58.4750623338	59.6169296878
C	37.4031163650	57.4157863141	60.4115416050
O	37.3631567167	57.9991882839	61.4894970916
N	38.3980958578	56.6008951577	60.0815290099
H	38.3467974166	56.1573159501	59.1942504623
C	39.5133887895	56.2424584449	60.9400679606
H	39.6216979638	56.9818686441	61.7155540089
C	39.1274217772	58.1262467159	65.2360186439
N	36.9939613869	57.3394884936	64.2817695015
C	38.0362586771	57.3447437271	65.1891264095
C	37.6514281671	56.3642112860	66.2132965466
C	36.4520993992	55.8666929046	65.9183109950
C	35.9962759435	56.4694061497	64.6583593775

C	34.8363929856	56.3144157667	63.9506032058
N	33.2365394770	55.3149703995	65.5740774560
C	33.7253625025	55.4609215531	64.3192921003
C	32.9152685399	54.6368939831	63.4528156832
C	31.9788348696	54.0194191929	64.1849540228
C	32.1528561400	54.4281366637	65.5962672969
O	31.5466929011	54.1265660103	66.5699149093
N	36.7902290780	60.9402369931	61.1213312528
C	35.7479166654	61.2936531403	60.3029038478
C	36.1362148311	62.5613147282	59.5773322406
C	37.5942434729	62.8194865043	59.9895630485
C	37.8970495532	61.7608959395	61.0431646872
O	34.7365038949	60.6716818626	60.2036484645
C	39.0457092953	61.7182856262	61.7700564172
N	38.8793624086	59.7200739771	63.2908913641
C	39.4474913612	60.8880415239	62.8704920234
C	40.4721364602	61.1927057127	63.7802894646
C	40.4927075394	60.2057219958	64.7439418591
C	39.4913205854	59.2757364235	64.4289400860
H	39.7507612263	62.5017080573	61.5558849611
H	34.7400619178	56.8551879556	63.0274675118
H	39.7977395137	57.9450789890	66.0502705830
H	38.2848580377	59.1597894740	62.7193795004
H	36.7370952752	60.0799549705	61.6204820646
H	33.5155960140	55.8379812109	66.3738811724
H	37.0367863157	57.6952526369	63.3567663465
H	38.2695942315	62.7112235702	59.1490110692
H	35.4640528941	63.3576207081	59.8730445782
H	38.2512682948	56.1401805409	67.0725114056
H	35.8969718758	55.1501991498	66.4869375932
H	33.0608930963	54.5646557927	62.3941439222
H	31.2140827622	53.3411560829	63.8669285043
H	36.0074102699	62.4079672799	58.5141042311
H	37.7360896651	63.8132820979	60.3928357999
H	41.0927797286	62.0649927612	63.7363084950
H	41.1250217100	60.1577256291	65.6057316476
H	36.6767388445	57.6215055871	58.3911816357
H	35.6257333678	56.7294094895	59.4768277163
H	39.3586814699	55.2712349660	61.3949075464
H	40.4201899996	56.2152375019	60.3526657390
54			
-135			
C	36.2899253324	57.5756945409	59.3999578494
H	35.7232064627	58.4672150020	59.6196306583

C	37.3986036767	57.4150318262	60.4096376781
O	37.3559698470	57.9992106472	61.4868130882
N	38.3951433415	56.6004095373	60.0794264328
H	38.3435948659	56.1536368639	59.1915644424
C	39.5114476248	56.2460796787	60.9408547697
H	39.6190747680	56.9895647015	61.7131537147
C	39.1233237458	58.1280418462	65.2309871989
N	37.0002829055	57.3251237369	64.2667058936
C	38.0354700474	57.3372003535	65.1787220279
C	37.6544089134	56.3535100718	66.1997687120
C	36.4634005212	55.8416659778	65.8983147147
C	35.9925906018	56.4641545376	64.6506830002
C	34.8183303793	56.3548409101	63.9731063870
N	33.2238664464	55.3351923607	65.5743370416
C	33.7189981407	55.4703620231	64.3284521690
C	32.9415960056	54.6201840851	63.4626666981
C	31.9938018967	54.0038682123	64.1874831544
C	32.1388453397	54.4388718352	65.5940735193
O	31.5283985824	54.1436132711	66.5662008513
N	36.7976407566	60.9417076575	61.1197917105
C	35.7558253338	61.2916231307	60.3011672802
C	36.1396368930	62.5645268752	59.5794942087
C	37.5982193429	62.8238213928	59.9902203517
C	37.9030161839	61.7671546306	61.0439719954
O	34.7476924319	60.6647477404	60.1962084062
C	39.0467359529	61.7218026629	61.7719757166
N	38.8723795287	59.7235041030	63.2921842043
C	39.4438906361	60.8901100372	62.8781346036
C	40.4682949104	61.1904739875	63.7841723941
C	40.4925011987	60.1957311070	64.7419999911
C	39.4892763585	59.2699161992	64.4288591062
H	39.7487296253	62.5058664997	61.5615606343
H	34.6906622773	56.9454155378	63.0870198738
H	39.7782986784	57.9560866978	66.0579103331
H	38.2979444114	59.1569376844	62.7061272681
H	36.7487969553	60.0755030400	61.6097676500
H	33.4858094507	55.8762319689	66.3710396245
H	37.0471955244	57.6780926078	63.3404099719
H	38.2709619790	62.7124562871	59.1487576528
H	35.4632059354	63.3542135764	59.8839816973
H	38.2466375069	56.1390377137	67.0659467340
H	35.9112531726	55.1245399776	66.4679097099
H	33.1172889251	54.5207455531	62.4121726172
H	31.2461058410	53.3050167538	63.8700627325

H	36.0060157151	62.4161909360	58.5151892070
H	37.7406908196	63.8171349168	60.3927854053
H	41.0847416245	62.0654310853	63.7438951420
H	41.1260260660	60.1436760984	65.6022589077
H	36.6736817247	57.6192430266	58.3881914482
H	35.6247764837	56.7210415358	59.4737583922
H	39.3572901090	55.2764524830	61.3997636388
H	40.4172928379	56.2175156235	60.3518671538
54			
-130			
C	36.2748014601	57.5307097987	59.3682624784
H	35.6847897828	58.4113178193	59.5718845338
C	37.3850881213	57.3953049186	60.3809231314
O	37.3411478188	57.9778661288	61.4598890204
N	38.3926553746	56.5940643793	60.0465080368
H	38.3415302318	56.1226899519	59.1701398449
C	39.5013491800	56.2576868891	60.9221406293
H	39.6066262464	57.0217247908	61.6747133782
C	39.1167631738	58.1208824777	65.2128522362
N	36.9426279683	57.4186593280	64.2961305175
C	38.0124184829	57.3555583201	65.1696898617
C	37.6595993449	56.3310660640	66.1542067542
C	36.4521036262	55.8461181328	65.8749603993
C	35.9464717434	56.5375967394	64.6754770208
C	34.7700115548	56.4265004269	64.0099407410
N	33.1813847275	55.3821416327	65.5991985603
C	33.7150702771	55.4889767740	64.3710660046
C	33.0223059207	54.5676185069	63.5087690209
C	32.0712925663	53.9387476341	64.2176330801
C	32.1199717371	54.4535154831	65.6051061604
O	31.4520637400	54.2005725215	66.5501294462
N	36.8138289012	60.9409712138	61.1181069128
C	35.7828063695	61.2884078319	60.2854282848
C	36.1584629325	62.5853489079	59.6013584931
C	37.6197089289	62.8334051125	60.0067345752
C	37.9120081901	61.7794160028	61.0632736392
O	34.7887747564	60.6464477491	60.1418994949
C	39.0489520646	61.7403812586	61.7971951731
N	38.8500766408	59.7393629726	63.3019829820
C	39.4307360037	60.9056676002	62.9045721496
C	40.4554318662	61.1956452454	63.8107663028
C	40.4753431306	60.1892652189	64.7569517665
C	39.4665009414	59.2753647109	64.4359408763
H	39.7563882034	62.5208699177	61.5869823031

H	34.6070678562	57.0281838726	63.1349000267
H	39.8041310041	57.9048174415	66.0031951276
H	38.2890347927	59.1717544979	62.7022290046
H	36.7730591766	60.0674971062	61.5957490620
H	33.3796022086	55.9866851488	66.3693389534
H	37.0267071051	57.6779376932	63.3402205840
H	38.2905772686	62.7017923265	59.1655682828
H	35.4906814585	63.3671991719	59.9429512715
H	38.2931371956	56.0638481982	66.9758477721
H	35.9224838022	55.0897773922	66.4180215377
H	33.2676529933	54.4381293532	62.4746200875
H	31.3726496970	53.1936266902	63.8947194602
H	36.0153532057	62.4760185573	58.5343902629
H	37.7771346079	63.8287583831	60.4010210276
H	41.0842707372	62.0624524173	63.7682817449
H	41.1205168622	60.1096469994	65.6064175365
H	36.6638448943	57.5745691522	58.3580085949
H	35.6278684555	56.6614079217	59.4415554584
H	39.3398503026	55.3021670586	61.4075587964
H	40.4140790039	56.2092793046	60.3456873629
54			
-125			
C	36.2725276705	57.5209273795	59.3539480792
H	35.6843430177	58.4020351384	59.5584210255
C	37.3795908952	57.3874697397	60.3694679300
O	37.3300038281	57.9709054812	61.4463731404
N	38.3914076716	56.5893953912	60.0412424792
H	38.3473908008	56.1259268148	59.1622601551
C	39.5000431671	56.2603499570	60.9181230602
H	39.6020140067	57.0282250892	61.6655393529
C	39.1201908317	58.1156227383	65.2081570066
N	36.9373386747	57.4339942196	64.2970338025
C	38.0168372337	57.3452644514	65.1586270559
C	37.6708515332	56.3041444431	66.1261720455
C	36.4547829439	55.8364569923	65.8534310998
C	35.9371003084	56.5550084365	64.6767963601
C	34.7489983341	56.4630937842	64.0343311109
N	33.1296696074	55.4296488157	65.6005782915
C	33.7058926471	55.5080024389	64.3930034053
C	33.0688106698	54.5425477019	63.5346525423
C	32.1063800035	53.9159624014	64.2291625461
C	32.0897606368	54.4720529674	65.6027238044
O	31.4009632825	54.2285281120	66.5349012944
N	36.8137691150	60.9481799910	61.1229364362

C	35.7868156980	61.2895910736	60.2842463605
C	36.1622975124	62.5890336190	59.6047409380
C	37.6248475941	62.8353149123	60.0064377252
C	37.9163564700	61.7831847070	61.0646858290
O	34.7945947622	60.6441648492	60.1377914771
C	39.0513982009	61.7417832304	61.7989123955
N	38.8475700968	59.7432560693	63.3072281944
C	39.4315209089	60.9068667728	62.9109475721
C	40.4553980861	61.1921083811	63.8173968452
C	40.4801060164	60.1791166012	64.7580941292
C	39.4713283724	59.2676419043	64.4352523766
H	39.7588678935	62.5218374461	61.5916764910
H	34.5661927041	57.0852913510	63.1799462670
H	39.8098752263	57.8956637828	65.9957059663
H	38.2792249727	59.1821125109	62.7099366627
H	36.7697175525	60.0745476038	61.5988711233
H	33.2969216404	56.0501021875	66.3661699122
H	37.0199752589	57.6974259724	63.3415547275
H	38.2923658934	62.6973957470	59.1640981985
H	35.4940763600	63.3676346448	59.9521877274
H	38.3095544413	56.0141990977	66.9367767340
H	35.9271869741	55.0733048758	66.3874064448
H	33.3668953795	54.3717869353	62.5197593251
H	31.4522652124	53.1299334168	63.9130140440
H	36.0137126256	62.4852872121	58.5389187461
H	37.7843818266	63.8310235580	60.3985357398
H	41.0793056158	62.0616674801	63.7824957858
H	41.1166949235	60.1042378885	65.6153510962
H	36.6621516704	57.5642575717	58.3447748186
H	35.6255855128	56.6528291893	59.4270136994
H	39.3402189121	55.3094000030	61.4107612558
H	40.4127234424	56.2096780671	60.3425251321
54			
-120			
C	36.2679534993	57.5426212208	59.3789383927
H	35.6864333587	58.4224457610	59.6041910439
C	37.3727623871	57.4006739237	60.3942637677
O	37.3206827498	57.9874300276	61.4694551842
N	38.3772457319	56.5937153973	60.0662861576
H	38.3356165408	56.1351808421	59.1827303141
C	39.4970259574	56.2725297892	60.9338651408
H	39.6051762748	57.0411172927	61.6822604175
C	39.1225060233	58.1099773299	65.2025138444
N	36.9628554446	57.3830312606	64.2691551701

C	38.0296529405	57.3188376200	65.1404871964
C	37.6820831530	56.2821171410	66.1122632077
C	36.4708921611	55.8018295413	65.8313085014
C	35.9595132091	56.5133249205	64.6504143309
C	34.7531505189	56.4612914667	64.0388644179
N	33.0891597597	55.4676818003	65.5836795470
C	33.6959516031	55.5135091957	64.3943939980
C	33.0845090394	54.5178317576	63.5446567978
C	32.1100835570	53.9030321660	64.2328056517
C	32.0685879561	54.4878427234	65.5961329074
O	31.3828146526	54.2458592185	66.5291858565
N	36.8336541864	60.9315567423	61.0978574102
C	35.7951208450	61.2755529122	60.2782751817
C	36.1551004001	62.5785353588	59.5996356992
C	37.6191983872	62.8403115989	59.9979949563
C	37.9273423774	61.7816757407	61.0497197370
O	34.8008432373	60.6292168571	60.1410262073
C	39.0578851307	61.7455588314	61.7922238482
N	38.8590199877	59.7356333181	63.2966829658
C	39.4391311162	60.9026176520	62.9052397375
C	40.4608141069	61.1880595281	63.8109168949
C	40.4849727109	60.1732832841	64.7528523744
C	39.4772373837	59.2606700578	64.4312293362
H	39.7589954908	62.5374471866	61.5988115971
H	34.5563599898	57.1174718994	63.2127793715
H	39.7919908183	57.9051848517	66.0131575156
H	38.3013524423	59.1709507065	62.6928906237
H	36.8045087003	60.0491531787	61.5579686686
H	33.2527310095	56.0947988871	66.3449734384
H	37.0259899807	57.6950261009	63.3281412935
H	38.2852154555	62.7225163403	59.1497006541
H	35.4760970673	63.3451884378	59.9524302751
H	38.3029027486	56.0156229635	66.9442859430
H	35.9251533528	55.0646228956	66.3838372684
H	33.3930480543	54.3501622192	62.5318379168
H	31.4586949696	53.1140481988	63.9159699784
H	36.0032413416	62.4791298135	58.5325249206
H	37.7695845511	63.8352770162	60.3976464110
H	41.0742040160	62.0659147897	63.7848833890
H	41.1189107998	60.1044550947	65.6129758783
H	36.6523876065	57.6024124247	58.3669659574
H	35.6184890081	56.6743203763	59.4419949828
H	39.3499122504	55.3193530127	61.4289288610
H	40.4040485959	56.2278124751	60.3468846258

54
-115

C	36.2672763670	57.5253724649	59.3590480224
H	35.6754126865	58.4048076232	59.5631805740
C	37.3714835888	57.3939339345	60.3794146485
O	37.3176102231	57.9745600243	61.4576173908
N	38.3836667186	56.5966657003	60.0482101146
H	38.3407331664	56.1320302668	59.1672590411
C	39.4968119951	56.2721742414	60.9233982690
H	39.6052017969	57.0471831371	61.6660258119
C	39.1299839049	58.1023995584	65.1877557163
N	36.9380758714	57.4189493880	64.2942810646
C	38.0230416775	57.3272545232	65.1394151198
C	37.6887450327	56.2802183863	66.0967109679
C	36.4667589420	55.8130413085	65.8332933826
C	35.9431258167	56.5362919989	64.6653245740
C	34.7319188149	56.4928394852	64.0621352240
N	33.0541524340	55.5019415356	65.5630722249
C	33.6952653625	55.5118681331	64.3983860055
C	33.1368634757	54.4570771855	63.5737372276
C	32.1377649119	53.8685622634	64.2470152629
C	32.0497693619	54.5037979878	65.5842039925
O	31.3412415918	54.2850960318	66.5047373962
N	36.8237009223	60.9380840611	61.1117850891
C	35.7958008140	61.2852356849	60.2806987235
C	36.1635056229	62.5894750576	59.6069248405
C	37.6268627400	62.8358920360	60.0074650858
C	37.9205440591	61.7834059365	61.0654623671
O	34.8030164088	60.6400380309	60.1286302334
C	39.0487349681	61.7481827420	61.8050663884
N	38.8472253538	59.7453916465	63.3093049769
C	39.4273056284	60.9110469902	62.9221831413
C	40.4483497976	61.1961324746	63.8230953765
C	40.4773825514	60.1752242329	64.7599199232
C	39.4735602087	59.2635275569	64.4367153838
H	39.7580711982	62.5271528342	61.5977366992
H	34.5148031728	57.1719381231	63.2589358635
H	39.8337152119	57.8565873030	65.9568267189
H	38.3101938411	59.1729891459	62.6930431228
H	36.7907667334	60.0574051357	61.5761922221
H	33.2173286974	56.1423299562	66.3158904614
H	36.9883095534	57.7468737703	63.3582950742
H	38.2952514762	62.7036316366	59.1646061196
H	35.4950744251	63.3638415912	59.9646599449

H	38.3340728088	55.9894885865	66.9011726946
H	35.9339565096	55.0678138381	66.3871670378
H	33.4708548219	54.2479972743	62.5778257099
H	31.4817498495	53.0802092635	63.9335338437
H	36.0130570445	62.4939056412	58.5392521091
H	37.7884260520	63.8305896139	60.4002328955
H	41.0766640190	62.0623396641	63.7846887893
H	41.1230162023	60.0965080080	65.6094745508
H	36.6640527920	57.5737421382	58.3512794490
H	35.6242489387	56.6521513467	59.4256655885
H	39.3382078469	55.3223293708	61.4226335536
H	40.4060806258	56.2178952763	60.3405097558
54			
-110			
C	36.2627205794	57.5163171746	59.3491467885
H	35.6615843607	58.3922735833	59.5433905889
C	37.3674136320	57.3947766908	60.3723025931
O	37.3130065309	57.9762281377	61.4503074648
N	38.3816961464	56.5985006655	60.0416021284
H	38.3412526269	56.1271174761	59.1622201311
C	39.4940806631	56.2784362939	60.9199981081
H	39.6048578524	57.0598593858	61.6572022937
C	39.1302338745	58.0935359901	65.1739236948
N	36.9182177487	57.4436430276	64.3076375973
C	38.0156516961	57.3288943318	65.1350014450
C	37.6973609604	56.2637887409	66.0707061307
C	36.4709296460	55.7981324405	65.8120924058
C	35.9292368390	56.5546555111	64.6760926379
C	34.7083053828	56.5244541541	64.0888899459
N	33.0001439444	55.5471877765	65.5562373529
C	33.6890961309	55.5242037260	64.4238104528
C	33.1937533569	54.4206754149	63.6192586398
C	32.1764370014	53.8371710403	64.2671054568
C	32.0164107844	54.5231161557	65.5756784556
O	31.2775678167	54.3232903096	66.4755119607
N	36.8258548979	60.9393280335	61.1127818072
C	35.7995520151	61.2876884707	60.2788991256
C	36.1678002880	62.5969699261	59.6130765591
C	37.6320922046	62.8382525359	60.0116353639
C	37.9214805004	61.7896860332	61.0735990772
O	34.8110717859	60.6412977648	60.1139955569
C	39.0481726692	61.7552849042	61.8126051964
N	38.8463747233	59.7430052185	63.3017647661
C	39.4266079947	60.9136837009	62.9288397803

C	40.4414934476	61.1957484199	63.8331830905
C	40.4707983827	60.1659774663	64.7615446070
C	39.4715014082	59.2578321228	64.4305279272
H	39.7613794332	62.5314600486	61.6032959006
H	34.4739921920	57.2160351059	63.2993450782
H	39.8452679653	57.8350953413	65.9308044873
H	38.3235602873	59.1705354282	62.6763908023
H	36.7976304853	60.0575657656	61.5746313102
H	33.1311823434	56.2174020698	66.2895733695
H	36.9702979836	57.7626394736	63.3695386643
H	38.2985115282	62.6974415426	59.1686386761
H	35.5051597703	63.3742740312	59.9763509421
H	38.3593855946	55.9558958571	66.8541555617
H	35.9551647521	55.0323909438	66.3531950742
H	33.5940012903	54.1796353116	62.6555109753
H	31.5487622401	53.0246859738	63.9512702564
H	36.0167583041	62.5088857145	58.5445121554
H	37.8002244683	63.8346720662	60.3970611027
H	41.0679879047	62.0634171959	63.8044162428
H	41.1194742812	60.0737487698	65.6074369055
H	36.6627308763	57.5617863790	58.3416032728
H	35.6261049200	56.6377592883	59.4178922546
H	39.3336637187	55.3315079359	61.4262703906
H	40.4047964046	56.2175742800	60.3386332120
54			
-105			
C	36.2425198339	57.5293633819	59.3624327092
H	35.6439131074	58.4011155969	59.5733192436
C	37.3447962976	57.4015734723	60.3899854796
O	37.2897185344	57.9949707653	61.4599925109
N	38.3579663610	56.5949846483	60.0706817726
H	38.3413266995	56.1432161734	59.1815132889
C	39.4888935547	56.3011829625	60.9381623000
H	39.6038870062	57.0896426646	61.6635686124
C	39.1559153114	58.0645410019	65.1542322155
N	36.9425770577	57.4018024373	64.2842307993
C	38.0469349373	57.2833301553	65.1013791160
C	37.7275541331	56.2059422023	66.0236582589
C	36.4846152393	55.7722982062	65.7868078429
C	35.9288303415	56.5551307056	64.6772956580
C	34.6963466097	56.5373754079	64.1073555837
N	32.8981555388	55.6126921421	65.5449751840
C	33.6683340409	55.5524323295	64.4641294306
C	33.2482503575	54.4008525838	63.6807047151

C	32.2237391088	53.8035243961	64.3020751533
C	31.9437024153	54.5500540543	65.5602906357
O	31.1535622472	54.3677494457	66.4167910333
N	36.8666105609	60.9166892977	61.0665823903
C	35.8175988989	61.2632388926	60.2585755567
C	36.1644549432	62.5914341428	59.6136907825
C	37.6318666968	62.8517150586	59.9989329553
C	37.9476702233	61.7952306826	61.0499906342
O	34.8301968886	60.6100774964	60.0997585780
C	39.0653913476	61.7701148012	61.8092085932
N	38.8937007438	59.7189000486	63.2735101840
C	39.4513067080	60.9064456487	62.9165735652
C	40.4482052750	61.1918288741	63.8408002084
C	40.4804997201	60.1553157285	64.7646499994
C	39.5004181244	59.2327137595	64.4147474116
H	39.7673378806	62.5648304329	61.6330810358
H	34.4631869111	57.2195237052	63.3125346462
H	39.8573608562	57.8174872491	65.9266641055
H	38.3032601980	59.1744085432	62.6825853536
H	36.8475251406	60.0334472798	61.5272500860
H	32.9343305746	56.3280509024	66.2465053462
H	36.9403647241	57.8314493216	63.3880823756
H	38.2867727951	62.7335330314	59.1429370709
H	35.4922346406	63.3517126669	59.9928869294
H	38.3995948282	55.8583411418	66.7822910946
H	35.9664470699	55.0019968622	66.3199173591
H	33.7176662447	54.1324288295	62.7546033734
H	31.6654871545	52.9396638837	63.9974800761
H	36.0020152389	62.5221003222	58.5468800269
H	37.7851725310	63.8486529710	60.3883943940
H	41.0548287635	62.0731184642	63.8410293465
H	41.1130165006	60.0822710209	65.6247167100
H	36.6397824547	57.5971362434	58.3568569858
H	35.6030551992	56.6537554392	59.4101759130
H	39.3487905365	55.3625533310	61.4602731562
H	40.3911055303	56.2414803406	60.3453479769
54			
-100			
C	36.2429386286	57.5267493511	59.3611497415
H	35.6460657810	58.3999454458	59.5774483862
C	37.3449668548	57.3970700217	60.3847905592
O	37.2855650997	57.9866705744	61.4565888136
N	38.3619503796	56.5990120056	60.0705145590
H	38.3335447403	56.1472565373	59.1850221108

C	39.4882681006	56.3028338563	60.9393668433
H	39.6023428574	57.0944968166	61.6613803333
C	39.1521007300	58.0638089354	65.1467820822
N	36.9391039457	57.3980412794	64.2824691891
C	38.0477844502	57.2766258153	65.0935511236
C	37.7317905138	56.2032005424	66.0160811419
C	36.4928096067	55.7578396181	65.7696472150
C	35.9437034119	56.5285702213	64.6555344982
C	34.6909014920	56.5433364374	64.1257259289
N	32.8766689325	55.6336699866	65.5223878695
C	33.6686874987	55.5528462201	64.4651343514
C	33.2683012715	54.3767544368	63.6958052356
C	32.2308872017	53.7937625573	64.3037475528
C	31.9419970753	54.5582082024	65.5467775706
O	31.1579468267	54.3785929319	66.4070574593
N	36.8659482172	60.9228702819	61.0711968099
C	35.8278818169	61.2700862930	60.2563823607
C	36.1678624100	62.5961754581	59.6119074848
C	37.6330892230	62.8526904132	60.0003657392
C	37.9465949957	61.7947564553	61.0508285076
O	34.8410898817	60.6156759811	60.1015788466
C	39.0621133137	61.7658367872	61.8077103410
N	38.8809162976	59.7285795706	63.2829644103
C	39.4481001839	60.9084005975	62.9204103990
C	40.4459057223	61.1917181213	63.8393726728
C	40.4830128778	60.1483920476	64.7601151509
C	39.5041494825	59.2293653155	64.4104328260
H	39.7580171222	62.5636893965	61.6347518162
H	34.4380706777	57.2619386360	63.3707749143
H	39.8497232553	57.8154745316	65.9203926182
H	38.3220111448	59.1747189973	62.6733293194
H	36.8470781084	60.0340438888	61.5191260900
H	32.9267177579	56.3418137898	66.2307925357
H	36.9356654742	57.8357898647	63.3910998664
H	38.2885073037	62.7299407379	59.1467534998
H	35.4914624978	63.3486074782	59.9995704334
H	38.3914349542	55.8731036880	66.7929791058
H	35.9640315368	55.0059249227	66.3169815077
H	33.7377702983	54.1021330920	62.7735052430
H	31.6664162656	52.9312709589	64.0056262062
H	36.0016028715	62.5260504730	58.5454368011
H	37.7894513900	63.8476616123	60.3915704350
H	41.0500730487	62.0745914552	63.8409909306
H	41.1138713820	60.0756239677	65.6215490472

H	36.6417299886	57.5938177597	58.3566714081
H	35.6072850361	56.6488775058	59.4135426060
H	39.3457889847	55.3647237559	61.4620135710
H	40.3870957164	56.2417855185	60.3434096953
54			
-95			
C	36.2347470136	57.4772262428	59.3376622701
H	35.6303632123	58.3491244102	59.5390559341
C	37.3352488901	57.3738000284	60.3670488033
O	37.2573838023	57.9508208271	61.4435890728
N	38.3729421287	56.6032088716	60.0425624766
H	38.3591568097	56.1564858948	59.1528753930
C	39.4913408182	56.3136704676	60.9231761167
H	39.6152706987	57.1227634773	61.6261293131
C	39.1582467839	58.0341848106	65.1024459557
N	36.8840186580	57.4689318967	64.3320338571
C	38.0275543623	57.2924421923	65.0859908078
C	37.7345911190	56.1932754945	65.9782549134
C	36.4841290765	55.7669841626	65.7590881289
C	35.9104994674	56.5796678922	64.6942943102
C	34.6603371835	56.5558746234	64.1565309817
N	32.8088966908	55.6755011806	65.4996567966
C	33.6750562367	55.5428982214	64.5132147841
C	33.3811380771	54.2844243770	63.8186117286
C	32.3018442315	53.7319750526	64.3748076580
C	31.8762165771	54.5952416209	65.5141034632
O	31.0030460037	54.4817640971	66.2942407431
N	36.8545800535	60.9522382320	61.0774260671
C	35.8357034945	61.3043704399	60.2391324197
C	36.1864186883	62.6454398149	59.6293100855
C	37.6579633336	62.8689585931	60.0105642069
C	37.9414666657	61.8203672536	61.0735022052
O	34.8570822363	60.6473398415	60.0447743130
C	39.0521869261	61.7933151686	61.8348580583
N	38.8710966936	59.7285561287	63.2673165174
C	39.4363166763	60.9202260676	62.9356729604
C	40.4358434957	61.1835227277	63.8505362953
C	40.4875886272	60.1105838327	64.7401550694
C	39.5100326353	59.2063293275	64.3763249295
H	39.7499967685	62.5932639926	61.6661818568
H	34.3910826994	57.2415542287	63.3778897950
H	39.8856111350	57.7416147006	65.8339316620
H	38.3545381752	59.1696351945	62.6239974367
H	36.8196702992	60.0691029025	61.5357564112

H	32.7688762510	56.4571294046	66.1295941390
H	36.8561342357	57.9372946419	63.4570003445
H	38.3103182344	62.7083878469	59.1598936099
H	35.5294508937	63.3943885030	60.0566825669
H	38.4200969222	55.8298674837	66.7163898368
H	35.9723468492	54.9895741429	66.2857182431
H	33.9646165621	53.9283279055	62.9920749490
H	31.7837618938	52.8324538653	64.1007925667
H	36.0082246165	62.6214217014	58.5631247689
H	37.8511585818	63.8642951637	60.3844332594
H	41.0276540549	62.0733283307	63.8783372529
H	41.1205550060	60.0127965377	65.5966170603
H	36.6347072390	57.5322209888	58.3314110927
H	35.6079848561	56.5923468782	59.4003829023
H	39.3302488121	55.3928569971	61.4724278735
H	40.3934531827	56.2220464664	60.3335115007

49

AnPixJ-QM_S0_MIN

C	25.0853522231	51.5468412361	30.4663864323
H	25.5821586520	50.6529577040	30.8354928982
H	24.3211389357	51.2489652672	29.7613295678
C	26.0989511401	52.4639095037	29.8021160191
O	26.9067758378	53.0642989934	30.6108285113
O	26.1295972907	52.5926019839	28.6018049000
C	30.0841589528	54.1077837254	28.3524673077
N	29.4681814737	52.2122170817	29.8763690666
C	30.2734961862	52.8312868994	28.9641210491
C	31.3745099899	52.0172071092	28.7674122306
C	31.2191224427	50.8875847849	29.5764247780
C	30.0161415721	51.0409627613	30.2480460821
C	29.2971883528	50.1553975959	31.1585506048
N	31.1731266003	48.7557470389	31.9275199470
C	29.8321392881	49.1363911251	31.8560409654
C	29.1046674591	48.1244608658	32.6371326026
C	29.9641680873	47.2374857344	33.1207242820
C	31.3420344044	47.6477176239	32.7291202906
O	32.3868808379	47.1567233998	33.0303120576
N	25.7507566999	55.3115877280	31.7148020252
C	24.8669021441	55.1935713112	32.7715561931
C	24.1991593271	56.5401036053	32.9659192902
C	24.6606849514	57.3695247069	31.7631408735
C	25.7257718207	56.5139409901	31.0926961298
O	24.6920281719	54.2025302253	33.3996552772
C	26.4628663981	56.9853256477	30.0644603471

N	28.0718110720	55.2001555660	29.3641897666
C	27.5607089811	56.4190823288	29.3288498222
C	28.3412747546	57.2056077462	28.3960067256
C	29.2997315547	56.4137326693	27.8994211507
C	29.1767087001	55.1119985145	28.5080297005
H	26.2363213521	57.9935168683	29.7701944743
H	28.2428921031	50.3307322474	31.2641630240
H	30.8560654173	54.3481060916	27.6432184418
H	27.6318642042	54.3835794204	29.8078457796
H	26.2531152279	54.4851565899	31.3987312559
H	31.9333304247	49.3671032708	31.7363240986
H	28.5558126468	52.5241099232	30.1853339150
H	23.8551217866	57.5401833907	31.0576189462
H	24.5300873204	56.9520647682	33.9116549685
H	24.6419658297	52.0379704104	31.3244071029
H	32.1926712779	52.2360823309	28.1082737192
H	31.8681446482	50.0382709239	29.6271587574
H	28.0365163705	48.1278951660	32.7361611582
H	29.7686210433	46.3608640159	33.7051876917
H	23.1276451847	56.4090625538	33.0248875601
H	25.0685010156	58.3336275759	32.0387542667
H	28.1588055949	58.2366518033	28.1746897862
H	30.0622587685	56.6757497884	27.1948000214

49

AnPixJ-QM_S1_MIN

C	25.0760990445	51.5213895624	30.5048277024
H	25.6208937995	50.6576226493	30.8789039343
H	24.2839420010	51.1799328540	29.8524378790
C	26.0459642876	52.4238676194	29.7627646140
O	26.8714294520	53.0565803582	30.5285832477
O	26.0342180178	52.5043788824	28.5599498273
C	30.0922981464	54.0173111086	28.2910183421
N	29.4506348546	52.3536817021	29.9903114109
C	30.2066724408	52.8587738031	28.9534641307
C	31.2720156086	51.8801607207	28.7368740859
C	31.1298681438	50.8811941324	29.6097992350
C	29.9438445202	51.1623441460	30.4201080248
C	29.3227456063	50.3952115958	31.3915300120
N	31.1880116465	48.8978412745	32.0909222869
C	29.8483767779	49.1948754785	31.9554806659
C	29.1259365099	48.0526187709	32.4559957913
C	29.9999818645	47.1410354760	32.9031422872
C	31.3646661655	47.6832209770	32.7470723347
O	32.4147126942	47.2378997318	33.0924373591

N	25.7625599566	55.2871265034	31.6656600780
C	24.8875234972	55.1500487356	32.7222744464
C	24.2191184748	56.4909844301	32.9473710070
C	24.6915396467	57.3549989416	31.7726156049
C	25.7492211893	56.5106726259	31.0794955685
O	24.7128801887	54.1470860922	33.3367567617
C	26.5026477878	57.0057627185	30.0601654653
N	28.1199500493	55.2152817656	29.3180379192
C	27.5810174370	56.4782390847	29.2950579628
C	28.3582431303	57.2353405574	28.3940655025
C	29.3474207795	56.4215572264	27.9007889508
C	29.1884326052	55.1556162071	28.4924916379
H	26.2741262538	58.0286754116	29.8090938836
H	28.3487060456	50.7033499590	31.7184209027
H	30.8325070212	54.1799398829	27.5352409947
H	27.6559213752	54.4001751941	29.7187149100
H	26.2473013134	54.4591054427	31.3216464814
H	31.9099065743	49.5837742384	32.1005802738
H	28.5105390777	52.6492177973	30.2249529722
H	23.8873682791	57.5588289982	31.0734067057
H	24.5398006616	56.8748646101	33.9089194177
H	24.6695873321	52.0362287013	31.3666881029
H	32.0406574265	51.9848960652	27.9974096960
H	31.7299465648	49.9978540352	29.6907938168
H	28.0612124590	47.9462715576	32.3900147366
H	29.8064206469	46.1647031408	33.2929765105
H	23.1464734040	56.3548236382	32.9895663961
H	25.1066176117	58.3060646678	32.0819854260
H	28.1934485631	58.2706478673	28.1670043500
H	30.1145275841	56.6763516732	27.2025122104

AnPixJ-QM_-145_-95_S1_SCAN_C14-C15-C16-C17

49

-145

C	25.0777844733	51.5223675496	30.5069938530
H	25.6220894784	50.6583789341	30.8811141958
H	24.2851709370	51.1814396463	29.8552190525
C	26.0451969096	52.4226412628	29.7592501170
O	26.8730940111	53.0583410427	30.5209162298
O	26.0282264367	52.4989489288	28.5565929322
C	30.0907251224	54.0245350258	28.2906278987
N	29.4410820771	52.3471442079	29.9740518422
C	30.2035538614	52.8578961705	28.9471242833
C	31.2693074293	51.8809642934	28.7323732352

C	31.1144285595	50.8722877020	29.5950116565
C	29.9249922866	51.1473915559	30.3939202690
C	29.3196108000	50.3986919443	31.3916124880
N	31.1757914246	48.9228844698	32.1622740409
C	29.8437208631	49.1859988098	31.9609078508
C	29.1266265617	48.0285826433	32.4243788637
C	30.0019453214	47.1269926760	32.8956735293
C	31.3610511196	47.6844180467	32.7661171935
O	32.4169220148	47.2336958497	33.0893140814
N	25.7643758632	55.2856141283	31.6647434385
C	24.8893974461	55.1481002853	32.7207880195
C	24.2200449170	56.4889914173	32.9455822900
C	24.6926370088	57.3528225203	31.7715056875
C	25.7507272570	56.5095135846	31.0777772573
O	24.7153575675	54.1465937811	33.3342489202
C	26.5034841283	57.0050614248	30.0616824612
N	28.1238581827	55.2181085700	29.3212219968
C	27.5842685054	56.4781574079	29.2975466594
C	28.3597927906	57.2363581289	28.3913127093
C	29.3449725231	56.4226280870	27.8964412017
C	29.1895438411	55.1541995103	28.4916075739
H	26.2785571942	58.0272543210	29.8082704390
H	28.3821700048	50.7506038616	31.7751473497
H	30.8329924653	54.1981311149	27.5385605517
H	27.6597205257	54.4052354423	29.7227601204
H	26.2474579766	54.4569554576	31.3209592446
H	31.9150311613	49.5868532098	32.0926259269
H	28.5080471842	52.6592784641	30.2227967396
H	23.8874245894	57.5583760395	31.0745840647
H	24.5415086260	56.8715839522	33.9073110546
H	24.6718041818	52.0381529353	31.3687603507
H	32.0457126106	51.9939542239	28.0009468014
H	31.7229069210	49.9972793269	29.6873522690
H	28.0618881256	47.9256032183	32.3719123882
H	29.8115644965	46.1539104921	33.2969235101
H	23.1474019763	56.3516267535	32.9872940624
H	25.1083031846	58.3023850586	32.0828454669
H	28.1948155751	58.2724660495	28.1659571995
H	30.1108480011	56.6790290863	27.1973924956

49

-140

C	25.0752365127	51.5304901067	30.5344503389
H	25.6226888172	50.6864504718	30.9462585732
H	24.2997435656	51.1592199583	29.8795656370

C	26.0406788306	52.4225306675	29.7743585786
O	26.8791245639	53.0574396960	30.5255387684
O	26.0090161322	52.4958364817	28.5715396116
C	30.0832419491	54.0269128129	28.2851847651
N	29.4348615823	52.3420304611	29.9655778205
C	30.1974424571	52.8559465617	28.9437968359
C	31.2621939867	51.8824831257	28.7422239353
C	31.1055099019	50.8728163432	29.6091118021
C	29.9186784885	51.1489968190	30.4022459015
C	29.3109556477	50.4039005023	31.4136970604
N	31.1730976020	48.9576644432	32.2508501153
C	29.8478038934	49.1732049695	31.9695785354
C	29.1587423094	47.9735980710	32.3508663009
C	30.0443490747	47.0863039549	32.8400162347
C	31.3832426867	47.6996904876	32.8085633769
O	32.4427777774	47.2775952583	33.1627414619
N	25.7710305199	55.2952535489	31.6653086847
C	24.9022505186	55.1680947068	32.7273980938
C	24.2390266228	56.5115678048	32.9488760739
C	24.7037064119	57.3629251776	31.7636698849
C	25.7587461366	56.5152190742	31.0694736248
O	24.7298320464	54.1715140402	33.3494227067
C	26.5080224367	57.0031460115	30.0513815926
N	28.1278240535	55.2136797942	29.3257129510
C	27.5899546313	56.4708460499	29.2926167597
C	28.3622651731	57.2236155122	28.3737163766
C	29.3390224094	56.4099387348	27.8749678778
C	29.1850802273	55.1434483441	28.4849929199
H	26.2846789101	58.0220921847	29.7896206987
H	28.3704517477	50.7619924734	31.7950102026
H	30.8210575224	54.2203745891	27.5336321836
H	27.6542708928	54.4085362253	29.7353257975
H	26.2557250701	54.4669067377	31.3270538559
H	31.8913414839	49.6450411451	32.2091283374
H	28.5141787595	52.6677740655	30.2296391083
H	23.8933685429	57.5609949648	31.0719109348
H	24.5695451813	56.9022856155	33.9043067278
H	24.6438630513	52.0650097145	31.3725936858
H	32.0374263910	51.9982919506	28.0117515404
H	31.7101662979	49.9968442369	29.7156012525
H	28.1036352739	47.8393367368	32.2315139964
H	29.8691649305	46.0924093837	33.1963817160
H	23.1667212654	56.3778790867	33.0020103296
H	25.1182254130	58.3156483927	32.0640116603

H	28.1949005456	58.2568180890	28.1468824879
H	30.1046213266	56.6667460484	27.1733811750
49			
-135			
C	25.0674283414	51.5426840088	30.5831433765
H	25.6103717369	50.6797953029	30.9609559460
H	24.2620773995	51.1974467584	29.9500301530
C	26.0266824164	52.4237383128	29.8022190754
O	26.8774730816	53.0621967080	30.5361324911
O	25.9804432711	52.4842013656	28.5993638097
C	30.0794333042	54.0238810870	28.2874094805
N	29.4285409599	52.3417932972	29.9713260560
C	30.1962531928	52.8537315236	28.9532671283
C	31.2665247458	51.8871118190	28.7624934449
C	31.1078246890	50.8792944287	29.6332678764
C	29.9154564523	51.1541902318	30.4147451694
C	29.3030766464	50.4056894699	31.4253466275
N	31.1547093830	48.9887521458	32.3256991888
C	29.8433239304	49.1710067003	31.9680952041
C	29.1748095398	47.9335533475	32.2547573352
C	30.0596792932	47.0566976001	32.7632422627
C	31.3755417315	47.7154972138	32.8431801416
O	32.4248433193	47.3134389631	33.2470432939
N	25.7825548597	55.3093715596	31.6682206014
C	24.9213573093	55.1867205831	32.7364692638
C	24.2395681192	56.5235643218	32.9411436452
C	24.7071622643	57.3743089982	31.7561171963
C	25.7582600070	56.5232680035	31.0595858835
O	24.7644693604	54.1966313155	33.3738827214
C	26.4962851283	57.0029800916	30.0303840642
N	28.1196203154	55.2114146571	29.3172977334
C	27.5779209978	56.4661769758	29.2725470081
C	28.3463743904	57.2130947102	28.3450796348
C	29.3261302743	56.3989720112	27.8543821448
C	29.1782050708	55.1361044334	28.4767140371
H	26.2670974515	58.0175996196	29.7578004489
H	28.3676169708	50.7666632046	31.8138245373
H	30.8196171791	54.2139574684	27.5367861742
H	27.6486007623	54.4078035319	29.7331898303
H	26.2669514147	54.4798819941	31.3328921963
H	31.8508168507	49.6995059908	32.3497994980
H	28.5058593972	52.6667259173	30.2313450210
H	23.8978682607	57.5803759556	31.0656367891
H	24.5495702766	56.9242837080	33.8991293213

H	24.6776918642	52.0726009135	31.4440951993
H	32.0477252200	52.0049569026	28.0391267326
H	31.7183588884	50.0087856016	29.7521126145
H	28.1348244933	47.7690507087	32.0615765229
H	29.8987063131	46.0430633910	33.0668379590
H	23.1683734762	56.3749770618	32.9791837917
H	25.1287728392	58.3232527951	32.0592591384
H	28.1739477691	58.2428673034	28.1078946700
H	30.0910115837	56.6518156425	27.1508334702
49			
-130			
C	25.0302352351	51.5856365737	30.5941379070
H	25.5519251258	50.7764838483	31.0980664304
H	24.2822548426	51.1693528498	29.9341959435
C	26.0342500912	52.4169570896	29.8154866758
O	26.8777381936	53.0584193895	30.5552709161
O	26.0241656350	52.4429576618	28.6104769080
C	30.0817216532	54.0239703753	28.3040117807
N	29.4343764478	52.3409031760	29.9910373180
C	30.2048865508	52.8526624333	28.9791174959
C	31.2817042023	51.8955078546	28.7969692123
C	31.1205387423	50.8856223926	29.6695316791
C	29.9258377738	51.1555145759	30.4367487100
C	29.3038385679	50.3988789355	31.4426121381
N	31.1402309084	49.0027595925	32.4013606623
C	29.8490174733	49.1555512485	31.9676911258
C	29.2091656739	47.8862705997	32.1489735740
C	30.0931033266	47.0134597837	32.6673085105
C	31.3759609059	47.7109566441	32.8631319195
O	32.4135388152	47.3225246475	33.3090132237
N	25.7860873492	55.3192300838	31.6751685706
C	24.9468874475	55.2162568438	32.7628672273
C	24.2832947859	56.5622260227	32.9668163151
C	24.6893882700	57.3728981352	31.7324638649
C	25.7473964245	56.5226952447	31.0452870882
O	24.7972407416	54.2353575338	33.4161845066
C	26.4739777094	56.9914714728	30.0048059492
N	28.1128326047	55.2041187740	29.3177736739
C	27.5600126046	56.4514933711	29.2534258941
C	28.3217074242	57.1919362066	28.3130725725
C	29.3073740606	56.3810654781	27.8354627894
C	29.1738555215	55.1228488912	28.4776090147
H	26.2337552038	57.9984384250	29.7155670099
H	28.3727812636	50.7605705777	31.8400122876

H	30.8267888876	54.2113616641	27.5576872305
H	27.6481995393	54.4030594027	29.7434093663
H	26.2659131274	54.4851754006	31.3456441307
H	31.8116772531	49.7319730566	32.4920323920
H	28.5074438384	52.6621711773	30.2456131118
H	23.8534408583	57.5136083719	31.0569965847
H	24.6568566918	56.9884881726	33.8908652285
H	24.5635393824	52.1842027818	31.3679579774
H	32.0688757696	52.0166110035	28.0803536465
H	31.7371804086	50.0204115110	29.7967476614
H	28.1897834718	47.6970592927	31.8821069295
H	29.9514656598	45.9797412624	32.9063502227
H	23.2155610910	56.4260181456	33.0754175373
H	25.0828679061	58.3501451257	31.9764172214
H	28.1388690298	58.2166718678	28.0614749506
H	30.0701930625	56.6286166953	27.1275968376
49			
-125			
C	25.0804167218	51.5361961355	30.6334168715
H	25.6513526146	50.6899218122	31.0067181458
H	24.2757376743	51.1696931402	30.0119953225
C	26.0003949575	52.4475591738	29.8405146579
O	26.8787896300	53.0677008458	30.5586196381
O	25.8980835246	52.5511680615	28.6441730401
C	30.0660306436	54.0115834207	28.2842310683
N	29.4226342701	52.3364476352	29.9834105555
C	30.1904360616	52.8392895973	28.9671186133
C	31.2642905462	51.8826896521	28.7896299432
C	31.1053101441	50.8791672157	29.6750418143
C	29.9175374364	51.1564485081	30.4390382685
C	29.2955566982	50.4093568489	31.4600564858
N	31.1140415272	49.0375510730	32.4841833816
C	29.8437365532	49.1653100367	31.9854624522
C	29.2344691866	47.8742680427	32.0914731775
C	30.1164167792	47.0112740044	32.6298846828
C	31.3643939211	47.7386839442	32.9165231798
O	32.3884087318	47.3650453507	33.4053728755
N	25.7937493665	55.3318631853	31.6727616035
C	24.9404971648	55.2244216073	32.7493566428
C	24.2694239224	56.5676441059	32.9469162541
C	24.7143373077	57.3950623051	31.7373342345
C	25.7647135871	56.5373936353	31.0465224956
O	24.7842704939	54.2419664861	33.3984064185
C	26.4937642913	57.0049157395	30.0076048246

N	28.1137482584	55.2047310131	29.3099166144
C	27.5702509265	56.4553764493	29.2486682079
C	28.3316265836	57.1893140295	28.2998719109
C	29.3045092860	56.3710566224	27.8152611790
C	29.1682109747	55.1119408097	28.4612321796
H	26.2622349297	58.0150291545	29.7214600131
H	28.3745624980	50.7843882328	31.8700851022
H	30.8081780310	54.1895418366	27.5322892360
H	27.6465525462	54.4093577260	29.7433545822
H	26.2773765177	54.4993591853	31.3456557606
H	31.7613625661	49.7814637556	32.6197550824
H	28.4995121152	52.6641586241	30.2463913127
H	23.8941021375	57.5755171292	31.0527030343
H	24.6054510279	56.9838963289	33.8894706118
H	24.6864961017	52.0573266325	31.4981330888
H	32.0502491009	51.9963797683	28.0701090680
H	31.7251360230	50.0173455010	29.8104122255
H	28.2365261545	47.6638130048	31.7649091674
H	29.9933547343	45.9670551756	32.8297715892
H	23.1989164252	56.4268314827	33.0134575863
H	25.1283195339	58.3557673910	32.0119286210
H	28.1536820753	58.2150969724	28.0481224477
H	30.0630282725	56.6107512946	27.0999346843
49			
-120			
C	25.0885430177	51.5497488741	30.6713157000
H	25.6467398954	50.6940614678	31.0425664517
H	24.2768900560	51.1956784099	30.0514531087
C	26.0226809827	52.4442135065	29.8756078700
O	26.8978166984	53.0693278482	30.5929481799
O	25.9351674382	52.5288967757	28.6764494055
C	30.0733485629	54.0066356306	28.2911996320
N	29.4389902556	52.3373171123	30.0015497925
C	30.2045925795	52.8356902327	28.9835577809
C	31.2798464580	51.8841151850	28.8093036766
C	31.1222524001	50.8818585453	29.7007033537
C	29.9379748219	51.1604640209	30.4599720326
C	29.3087686945	50.4107952899	31.4824204152
N	31.0996179070	49.0590612942	32.5761150056
C	29.8598339089	49.1647073546	32.0016140361
C	29.2902907853	47.8528967795	32.0164680407
C	30.1674353198	46.9964979917	32.5741925382
C	31.3679945854	47.7518579030	32.9706661323
O	32.3713363509	47.3913470957	33.5100227344

N	25.8161624929	55.3497494945	31.6836340403
C	24.9779548917	55.2574468849	32.7732626724
C	24.2983498295	56.5989374979	32.9523129043
C	24.7126842289	57.4008649640	31.7148227461
C	25.7681419997	56.5420572747	31.0330081144
O	24.8390782857	54.2869416492	33.4445285034
C	26.4840067142	56.9956396718	29.9798131958
N	28.1173462383	55.1981046856	29.3075250391
C	27.5635346332	56.4420978120	29.2272163683
C	28.3157926619	57.1671745757	28.2636642773
C	29.2936034456	56.3505839122	27.7899770348
C	29.1711608784	55.0988867391	28.4565206976
H	26.2398821718	57.9969750020	29.6749099923
H	28.3931129314	50.7912690454	31.8990032048
H	30.8157045109	54.1800027603	27.5382846595
H	27.6572007793	54.4063434367	29.7532868622
H	26.3005276200	54.5145052496	31.3659301913
H	31.7145105410	49.8162151776	32.7753463709
H	28.5157471799	52.6659520773	30.2667604060
H	23.8800966922	57.5467731526	31.0367249455
H	24.6505752831	57.0399415223	33.8775998236
H	24.7054855832	52.0770172695	31.5371028165
H	32.0657554613	51.9964590196	28.0895030840
H	31.7452142423	50.0230961136	29.8411542740
H	28.3228226941	47.6241064554	31.6182626157
H	30.0698399688	45.9407129595	32.7209503251
H	23.2306228773	56.4512668437	33.0444767201
H	25.1129601679	58.3759826036	31.9566814682
H	28.1274569660	58.1873426470	27.9967037531
H	30.0484750383	56.5845298934	27.0687020346
49			
-115			
C	25.0697073013	51.5874121297	30.7312730312
H	25.6090979675	50.7709899867	31.2041970905
H	24.2895514473	51.1783495039	30.1049850127
C	26.0410210322	52.4162562736	29.9094129156
O	26.9057813218	53.0716681351	30.6122421894
O	25.9903478599	52.4258869579	28.7052610773
C	30.0824043198	54.0089463182	28.3020962084
N	29.4469379287	52.3360853679	30.0104133923
C	30.2179708835	52.8366771105	28.9987327307
C	31.2977254044	51.8939413433	28.8322428769
C	31.1361453634	50.8881869482	29.7233094474
C	29.9478987337	51.1622320049	30.4690884225

C	29.3082167114	50.4028613005	31.4868712158
N	31.0616978613	49.0601057098	32.6464926299
C	29.8598262694	49.1525989007	31.9947857003
C	29.3370851104	47.8246380590	31.9107449017
C	30.2062444243	46.9708332817	32.4853543744
C	31.3504756965	47.7446193411	32.9958474078
O	32.3289806516	47.3909270533	33.5832342694
N	25.8239155312	55.3606364218	31.6874835148
C	25.0030745491	55.2853077835	32.7914630816
C	24.3256685801	56.6292123416	32.9597774301
C	24.7043710420	57.4029367144	31.6935004076
C	25.7618282353	56.5419434651	31.0176267452
O	24.8760941863	54.3258959190	33.4805705765
C	26.4663071343	56.9844909839	29.9531220483
N	28.1134238186	55.1918567876	29.3010914765
C	27.5505659949	56.4298991943	29.2061229900
C	28.2993825479	57.1514406822	28.2366956079
C	29.2856451671	56.3387283579	27.7755187430
C	29.1721441185	55.0915643389	28.4545404018
H	26.2122741936	57.9785257674	29.6336106534
H	28.3970869131	50.7870752260	31.9094050994
H	30.8294568817	54.1838768865	27.5538498231
H	27.6599159221	54.4015868555	29.7557738968
H	26.3068007773	54.5218043951	31.3766802692
H	31.6406031015	49.8255707147	32.9109531784
H	28.5223446932	52.6647840740	30.2733884319
H	23.8570925865	57.5131764434	31.0268022691
H	24.7050136952	57.0916285226	33.8638070498
H	24.6432507131	52.1837214120	31.5294736940
H	32.0897512613	52.0098001585	28.1201215052
H	31.7623739841	50.0326760187	29.8689409408
H	28.4062783677	47.5849248806	31.4383017936
H	30.1372846020	45.9063642787	32.5754234702
H	23.2614155663	56.4830566072	33.0868263020
H	25.0905300887	58.3917798644	31.8992715555
H	28.1033260643	58.1668829045	27.9585125813
H	30.0414271682	56.5709909904	27.0548356263
49			
-110			
C	25.0648524007	51.5973757438	30.7307921572
H	25.5991100874	50.7597129807	31.1715438181
H	24.2667668585	51.2165425156	30.1092142477
C	26.0331107132	52.4317309486	29.9108183585
O	26.9096698332	53.0722301379	30.6132617239

O	25.9694692843	52.4588124289	28.7076236669
C	30.0812251022	53.9989711291	28.2943481363
N	29.4441727848	52.3269367626	30.0054308772
C	30.2179613105	52.8239559197	28.9956159221
C	31.2971229428	51.8850639167	28.8345043049
C	31.1334009333	50.8801975714	29.7319170160
C	29.9472196655	51.1567949418	30.4688381263
C	29.2991548448	50.4001258509	31.4934204448
N	31.0157483855	49.0936689616	32.7441383111
C	29.8576818059	49.1560176378	32.0145152667
C	29.3982217839	47.8138512817	31.8577142697
C	30.2628797984	46.9782857875	32.4676517031
C	31.3363911197	47.7810244686	33.0755298818
O	32.2884542459	47.4493396964	33.7176495647
N	25.8436670092	55.3672558414	31.6909221743
C	25.0266441587	55.2942030646	32.7982745765
C	24.3474027400	56.6374396367	32.9642004523
C	24.7285400419	57.4122945865	31.6992493708
C	25.7799557356	56.5471155408	31.0186770238
O	24.9033169257	54.3368143640	33.4909993656
C	26.4793412579	56.9859404815	29.9490321641
N	28.1188231918	55.1873594042	29.2966169405
C	27.5587706572	56.4252341928	29.1985701401
C	28.3043747104	57.1412443893	28.2217365633
C	29.2862354450	56.3249573796	27.7596557594
C	29.1748218466	55.0795258458	28.4452019290
H	26.2256651405	57.9794789710	29.6270847024
H	28.3901668402	50.7914175900	31.9145713833
H	30.8285983395	54.1691460422	27.5448061747
H	27.6665653936	54.4002221372	29.7574577337
H	26.3249429788	54.5278055020	31.3799545727
H	31.5476097217	49.8735549268	33.0595666092
H	28.5204776049	52.6589219029	30.2703373141
H	23.8809007403	57.5315871719	31.0345859595
H	24.7216183554	57.1007928946	33.8697495084
H	24.6629834670	52.1796141945	31.5517474066
H	32.0913314353	51.9980623007	28.1245499916
H	31.7610800516	50.0265771545	29.8824952553
H	28.5117513655	47.5511258071	31.3167050336
H	30.2319247134	45.9099037592	32.5241580981
H	23.2829016844	56.4897220660	33.0866575116
H	25.1218061260	58.3977832140	31.9081243082
H	28.1087925784	58.1557824457	27.9394999271
H	30.0385317035	56.5518902657	27.0334973605

49
-105

C	25.0965746933	51.5777607346	30.8059300519
H	25.6587676413	50.7436416690	31.2172957940
H	24.2779998271	51.1914953346	30.2158550698
C	26.0205764428	52.4280810829	29.9522182485
O	26.9129305892	53.0786994122	30.6254829983
O	25.9116281474	52.4577366175	28.7524176142
C	30.0742770493	54.0080629676	28.2923210416
N	29.4461383829	52.3355926755	30.0083295714
C	30.2186453586	52.8321573303	28.9994506939
C	31.2993476392	51.8993715870	28.8402868766
C	31.1353186110	50.8913689147	29.7402005455
C	29.9526457683	51.1664447799	30.4706657115
C	29.2988355633	50.4114246652	31.5026601899
N	30.9544705357	49.0759885521	32.8037276530
C	29.8437321352	49.1512203895	32.0071620693
C	29.4145751966	47.8157890707	31.7665057761
C	30.2525323272	46.9644381091	32.3957046954
C	31.2733672284	47.7540776531	33.0997920451
O	32.1900244114	47.4091504653	33.7860428783
N	25.8418796693	55.3797359409	31.6910084014
C	25.0287983580	55.3133795639	32.8017991400
C	24.3395042590	56.6524180215	32.9554976469
C	24.7147708490	57.4174468439	31.6831046703
C	25.7681772165	56.5508882187	31.0068643159
O	24.9168498082	54.3625884072	33.5053010113
C	26.4608302872	56.9830120098	29.9299314203
N	28.1061840023	55.1893707392	29.2881516819
C	27.5418582199	56.4220516329	29.1823879163
C	28.2828894568	57.1362132430	28.2014088278
C	29.2687988155	56.3221163078	27.7441503579
C	29.1650285660	55.0798967341	28.4369034829
H	26.2004923866	57.9722165652	29.6010698440
H	28.4024637717	50.8164318315	31.9375825330
H	30.8227119610	54.1767206160	27.5442289121
H	27.6556570738	54.4029245739	29.7508235766
H	26.3254736151	54.5398088896	31.3856223289
H	31.4583596550	49.8482808260	33.1783245563
H	28.5214201318	52.6656072313	30.2739842555
H	23.8654844171	57.5281030602	31.0193629457
H	24.7101378811	57.1269876016	33.8566504929
H	24.7231698767	52.1503882202	31.6466460279
H	32.0956665694	52.0141297563	28.1334937758

H	31.7648643175	50.0399220558	29.8926455842
H	28.5664456043	47.5656786047	31.1619455005
H	30.2327722345	45.8942011131	32.4074962062
H	23.2763479117	56.4964710705	33.0791397401
H	25.1052228489	58.4057993857	31.8825965570
H	28.0830302653	58.1488259234	27.9134674432
H	30.0210901873	56.5491457634	27.0172174519
49			
-100			
C	25.0988593748	51.5838526233	30.8116982479
H	25.6507240975	50.7438111552	31.2260837841
H	24.2795935083	51.2035843509	30.2179885757
C	26.0329663903	52.4252468348	29.9603986895
O	26.9202070894	53.0815197602	30.6350162589
O	25.9366679902	52.4425931786	28.7595611836
C	30.0650886893	54.0051418486	28.2782759463
N	29.4475542553	52.3383986728	30.0047777881
C	30.2149541605	52.8298264932	28.9892106447
C	31.2918233929	51.8981981133	28.8269464734
C	31.1321389229	50.8919352973	29.7331332327
C	29.9560777223	51.1718325859	30.4677717144
C	29.3082472975	50.4238968053	31.5136661023
N	30.9077941750	49.0758943284	32.8742813877
C	29.8483048976	49.1545617487	32.0120774931
C	29.4602387331	47.8235601911	31.7053608711
C	30.2745179820	46.9673886832	32.3626413809
C	31.2352388076	47.7515623747	33.1497131959
O	32.1179325480	47.4025823382	33.8775600343
N	25.8518778697	55.3799117808	31.6964031001
C	25.0463326720	55.3140614242	32.8128669708
C	24.3537451638	56.6513448295	32.9669188667
C	24.7237720705	57.4178065512	31.6937320737
C	25.7724246265	56.5502392522	31.0108015663
O	24.9410110105	54.3651694578	33.5199452401
C	26.4570929917	56.9812203053	29.9279943130
N	28.1017227140	55.1866951786	29.2817911556
C	27.5329320852	56.4181235638	29.1744916830
C	28.2667566147	57.1288970809	28.1831710863
C	29.2500332131	56.3158051367	27.7235232001
C	29.1541674016	55.0755131067	28.4239701263
H	26.1936909614	57.9695688307	29.5963589020
H	28.4203255520	50.8371993902	31.9600205104
H	30.8080934199	54.1714752423	27.5230434386
H	27.6593606991	54.4026218278	29.7564227566

H	26.3375914906	54.5413550724	31.3906481654
H	31.3778834920	49.8466274113	33.2925828484
H	28.5261829993	52.6715793080	30.2785132719
H	23.8712384523	57.5330076909	31.0346198761
H	24.7226267324	57.1283928843	33.8674135274
H	24.7255261241	52.1592566230	31.6507287546
H	32.0833708397	52.0075966815	28.1137765467
H	31.7621278244	50.0403486229	29.8843454819
H	28.6538085688	47.5788052060	31.0436052744
H	30.2712164659	45.8975120014	32.3453028859
H	23.2911958057	56.4926011810	33.0913495135
H	25.1173083122	58.4052292981	31.8935509283
H	28.0611066258	58.1391330270	27.8909029148
H	29.9954809339	56.5400184172	26.9888171539
49			
-95			
C	25.1232903711	51.5816337541	30.8639053457
H	25.6920508433	50.7432457384	31.2576623524
H	24.2891171987	51.2007634409	30.2913585997
C	26.0305357130	52.4323331434	29.9930133523
O	26.9357418878	53.0839101953	30.6481516199
O	25.8988299045	52.4636316517	28.7959890587
C	30.0617853203	54.0051753624	28.2652437940
N	29.4556419127	52.3358294308	29.9929920892
C	30.2201942475	52.8275321331	28.9748122901
C	31.2944484364	51.9027418281	28.8060889498
C	31.1396528474	50.8935314961	29.7142971232
C	29.9682224498	51.1724222612	30.4514309963
C	29.3294180156	50.4303148694	31.5081091358
N	30.8542248006	49.0715549820	32.9388138703
C	29.8626222281	49.1530799581	32.0002394268
C	29.5224377977	47.8281329434	31.6338149762
C	30.2987601336	46.9665119383	32.3350359975
C	31.1851385609	47.7459263623	33.2080261355
O	32.0206299779	47.3956753216	33.9898490040
N	25.8671953355	55.3903201412	31.7038193426
C	25.0633092298	55.3265956448	32.8218563843
C	24.3646971523	56.6622243226	32.9686054536
C	24.7219762596	57.4183507909	31.6851608020
C	25.7765065662	56.5542675229	31.0091003481
O	24.9632788795	54.3811844388	33.5341671723
C	26.4550292864	56.9805465881	29.9211429281
N	28.1026952184	55.1860084494	29.2771240451
C	27.5306074252	56.4175599319	29.1679728130

C	28.2608965237	57.1277233156	28.1699926893
C	29.2428497826	56.3162146322	27.7097025684
C	29.1505264903	55.0754069646	28.4153960349
H	26.1853281035	57.9639411763	29.5838444798
H	28.4441477903	50.8433359569	31.9578397609
H	30.8010298312	54.1734736041	27.5056610627
H	27.6591680504	54.4004217111	29.7512793174
H	26.3541278876	54.5508093379	31.4023014125
H	31.2839518947	49.8390594215	33.4036919777
H	28.5342549894	52.6674745452	30.2687876524
H	23.8661479274	57.5136469250	31.0272594902
H	24.7405393875	57.1477606924	33.8618613913
H	24.7722715557	52.1518880760	31.7160056570
H	32.0838350802	52.0148212430	28.0909096050
H	31.7734738318	50.0442818114	29.8637944575
H	28.7706471360	47.5869134159	30.9098993172
H	30.3108725878	45.8971456657	32.2976534918
H	23.3035908355	56.5005176609	33.1044056215
H	25.1053623531	58.4122631904	31.8711713378
H	28.0512744076	58.1355044068	27.8776064738
H	29.9861243698	56.5376473946	26.9737089549
50			
AnPixJ-COOH-QM_S0_MIN			
C	24.7921122865	51.4749129872	31.0437209431
H	25.4084551106	50.6400419183	31.3552503541
H	23.8449114163	51.1142686911	30.6735654267
C	25.5247401117	52.2476094993	29.9921810746
O	26.6111115492	52.7565069131	30.1532031177
C	30.2605573187	54.1987410124	28.5336659592
N	29.5361705853	52.2251481371	29.9164250121
C	30.4303514580	52.9715083487	29.1559403693
C	31.6296196789	52.2264503638	29.1379768732
C	31.4509620257	51.0832084426	29.8621366886
C	30.1155142185	51.0899847615	30.3272715224
C	29.3747966805	50.0526437930	31.0277186216
N	31.2542181937	48.8235296858	32.0426018642
C	29.9190863988	49.0425125527	31.7399359046
C	29.1904094798	47.8859916372	32.2930065292
C	30.0473951468	47.0718204857	32.8878116355
C	31.4086666989	47.6719791803	32.8103274908
O	32.4238824617	47.2990629228	33.2934934512
N	25.8083545681	55.4254839551	31.5516087193
C	24.9715811154	55.2823750412	32.6352955141
C	24.3692346091	56.6401357084	32.9234886172

C	24.7188812624	57.4856379568	31.6881008609
C	25.7816607055	56.6760032872	30.9602584909
O	24.7912894010	54.2530861770	33.2050087511
C	26.5158545810	57.1378195335	29.9286319656
N	28.1911908338	55.3402601250	29.3891626300
C	27.6100299848	56.5298797528	29.1871261817
C	28.2989716736	57.1782449997	28.1513113044
C	29.3402870806	56.3669180798	27.7775101542
C	29.2959988184	55.1956969427	28.5530648011
H	26.3133133046	58.1474746217	29.6239407366
H	28.3050251622	50.0764627269	30.9456497635
H	31.0965236217	54.4499990695	27.9048122322
H	27.9203252650	54.6790091058	30.0767593031
H	26.2557843588	54.6144197892	31.1883660061
H	31.9344938591	49.5471509610	32.1025829411
H	28.5597964036	52.4158496854	30.0398594883
H	23.8567806387	57.6054980197	31.0413227633
H	24.8218270978	57.0253128401	33.8306831390
H	24.6454090015	52.1153599224	31.9051290480
H	32.5292205252	52.5379985589	28.6453941195
H	32.1589649051	50.2950546874	30.0066569351
H	28.1347586603	47.7452576917	32.1688146127
H	29.8594790053	46.1350035230	33.3686590994
H	23.3077253676	56.5403821810	33.1023494144
H	25.0782817100	58.4738385874	31.9395447157
H	28.0506958360	58.1452200724	27.7654470032
H	30.0742735284	56.5705552797	27.0253502142
O	24.8846671617	52.3477309952	28.8568023060
H	25.3975087154	52.8478648273	28.2276347671
50			
AnPixJ-COOH-QM_S1_MIN			
C	24.7542895724	51.4500510425	31.0056877002
H	25.3788291924	50.6173286588	31.3078441290
H	23.8286547057	51.0812227734	30.5918199352
C	25.5090475202	52.2734211416	30.0096520951
O	26.5749138243	52.7974270225	30.2343020451
C	30.2628114875	54.0999372806	28.4237879416
N	29.5095670478	52.3767285422	30.0315328492
C	30.3722551188	52.9547492296	29.1114541524
C	31.5532716673	52.0841665364	29.0772540173
C	31.3845678121	51.0789467100	29.9301237058
C	30.0375581322	51.1900367748	30.5146749817
C	29.3484486658	50.2887403867	31.2641314719
N	31.1867003082	48.9897429533	32.3217225478

C	29.9291443661	49.0832396876	31.8220493901
C	29.2999157253	47.8043982536	31.9960631813
C	30.1650921204	46.9657967712	32.5903907955
C	31.4041977258	47.7197898883	32.8844216716
O	32.3861769176	47.4000841983	33.4683783928
N	25.8785357750	55.4277824087	31.6419393350
C	25.0090287217	55.3038170198	32.7014170517
C	24.3615110837	56.6542518699	32.9168947563
C	24.7395852550	57.4668220253	31.6688881548
C	25.8283061727	56.6469359617	30.9930012872
O	24.8335912745	54.2917379634	33.3047070312
C	26.5585567359	57.0876516776	29.9351527960
N	28.2409124255	55.2936488247	29.3531491225
C	27.6363970684	56.5081014019	29.1895629200
C	28.3114777924	57.1587594704	28.1448797889
C	29.3219614819	56.3297339554	27.7080199479
C	29.2860204322	55.1626685438	28.4829132103
H	26.3126996271	58.0795185245	29.6005968592
H	28.3011623524	50.4425508417	31.4331559532
H	31.0602010374	54.2978438925	27.7400222455
H	27.9693691033	54.5983341266	30.0082136044
H	26.3690590626	54.6156342658	31.3378232175
H	31.7615731000	49.7670543886	32.5640769311
H	28.5272202919	52.5259444745	30.0389406728
H	23.8939160779	57.5656578107	30.9967592588
H	24.7647812965	57.0823152611	33.8277787110
H	24.5663784090	52.0560728913	31.8839428207
H	32.4117796552	52.2861741567	28.4699028073
H	32.0688410271	50.2813385696	30.1299169222
H	28.3069438630	47.5885778041	31.6601531614
H	30.0337730158	45.9369399466	32.8481420765
H	23.2962432701	56.5333024537	33.0563234121
H	25.0883694492	58.4636380763	31.9030818214
H	28.0732642027	58.1405103871	27.7857847521
H	30.0369444722	56.5272967062	26.9370792825
O	24.9174597899	52.3967883807	28.8498515192
H	25.4498443487	52.9296941048	28.2651970033

AnPixJ-COOH-QM_-145_-95_S1_SCAN_C14-C15-C16-C17

50

-145

C	24.7542895738	51.4500510430	31.0056876993
H	25.3788291938	50.6173286589	31.3078441287
H	23.8286547072	51.0812227727	30.5918199329

C	25.5090475166	52.2734211403	30.0096520959
O	26.5749138305	52.7974270254	30.2343020485
C	30.2628114893	54.0999372876	28.4237879508
N	29.5095670500	52.3767285509	30.0315328478
C	30.3722551135	52.9547492235	29.1114541469
C	31.5532716677	52.0841665324	29.0772540010
C	31.3845678181	51.0789467046	29.9301237186
C	30.0375581343	51.1900367850	30.5146750071
C	29.3484486453	50.2887403812	31.2641314591
N	31.1867003117	48.9897429638	32.3217225507
C	29.9291443742	49.0832396766	31.8220493712
C	29.2999157209	47.8043982670	31.9960631859
C	30.1650921342	46.9657967417	32.5903907843
C	31.4041977068	47.7197898959	32.8844216768
O	32.3861769274	47.4000841960	33.4683784023
N	25.8785357761	55.4277824085	31.6419393359
C	25.0090287240	55.3038170382	32.7014170432
C	24.3615110835	56.6542518691	32.9168947565
C	24.7395852538	57.4668220253	31.6688881559
C	25.8283061714	56.6469359611	30.9930012905
O	24.8335912734	54.2917379498	33.3047070417
C	26.5585567363	57.0876516787	29.9351527959
N	28.2409124207	55.2936488234	29.3531491180
C	27.6363970693	56.5081014002	29.1895629190
C	28.3114777943	57.1587594714	28.1448797865
C	29.3219614792	56.3297339522	27.7080199448
C	29.2860204339	55.1626685400	28.4829132117
H	26.3126996271	58.0795185249	29.6005968591
H	28.3011623591	50.4425508315	31.4331559480
H	31.0602010293	54.2978438874	27.7400222427
H	27.9693690991	54.5983341223	30.0082135994
H	26.3690590661	54.6156342674	31.3378232203
H	31.7615730934	49.7670543861	32.5640769337
H	28.5272202932	52.5259444786	30.0389406728
H	23.8939160778	57.5656578102	30.9967592583
H	24.7647812954	57.0823152624	33.8277787107
H	24.5663784081	52.0560728901	31.8839428205
H	32.4117796527	52.2861741605	28.4699028067
H	32.0688410277	50.2813385665	30.1299169214
H	28.3069438659	47.5885778106	31.6601531652
H	30.0337730158	45.9369399544	32.8481420768
H	23.2962432697	56.5333024540	33.0563234112
H	25.0883694489	58.4636380766	31.9030818201
H	28.0732642033	58.1405103919	27.7857847552

H	30.0369444713	56.5272967066	26.9370792830
O	24.9174597931	52.3967883827	28.8498515197
H	25.4498443527	52.9296941078	28.2651970052

50

-140

C	24.7586383042	51.4490895029	31.0034617616
H	25.3842103274	50.6176916835	31.3069402491
H	23.8364693202	51.0787702904	30.5834300830
C	25.5169268001	52.2782892218	30.0150825008
O	26.5791618276	52.8044686695	30.2499913608
O	24.9337612164	52.4044261971	28.8515058790
H	25.4671536145	52.9442853458	28.2740539934
C	30.2503069163	54.0919685153	28.4116795140
N	29.4991889428	52.3673741992	30.0205009917
C	30.3627753673	52.9482949510	29.1033860363
C	31.5446214292	52.0815457796	29.0711817940
C	31.3714189857	51.0656298271	29.9092956667
C	30.0304503785	51.1828049612	30.5050584393
C	29.3548265000	50.3113841191	31.2976607686
N	31.1841867976	48.9974378816	32.3478965160
C	29.9338717578	49.0922787915	31.8355003801
C	29.3157627998	47.8018199291	31.9670417649
C	30.1674419631	46.9684284178	32.5850890100
C	31.3992422589	47.7193686283	32.9033790847
O	32.3727430260	47.3990112801	33.4989393718
N	25.8831078478	55.4321687284	31.6481915019
C	25.0124232800	55.3073572189	32.7059099674
C	24.3600109467	56.6564851828	32.9174192506
C	24.7386036117	57.4678319637	31.6683323521
C	25.8306315919	56.6495735838	30.9949178452
O	24.8384192173	54.2964439308	33.3102150777
C	26.5582308093	57.0873793351	29.9354911454
N	28.2309136408	55.2862188513	29.3449219963
C	27.6363849574	56.5056712188	29.1882338699
C	28.3161371923	57.1579755138	28.1487726270
C	29.3202526514	56.3238012509	27.7060316568
C	29.2759173958	55.1523015124	28.4724795730
H	26.3143769218	58.0794508100	29.6009129369
H	28.3242186418	50.5052735415	31.5214359665
H	31.0552807129	54.2970021549	27.7381172608
H	27.9468089387	54.5852719322	29.9887986787
H	26.3763070020	54.6208906215	31.3471753997
H	31.7343420018	49.7789826747	32.6361575698
H	28.5147293807	52.5039426118	30.0209514737

H	23.8938102854	57.5621859610	30.9946233648
H	24.7593197205	57.0876900567	33.8286906700
H	24.5644487700	52.0527803386	31.8820005652
H	32.4084262715	52.2895990677	28.4733250714
H	32.0473546457	50.2564571718	30.0893927116
H	28.3486051661	47.5669653883	31.5734051717
H	30.0491607587	45.9278332023	32.7987316732
H	23.2947663071	56.5313144461	33.0548053088
H	25.0821596306	58.4665127410	31.9025473688
H	28.0859534455	58.1427101308	27.7935326510
H	30.0368893043	56.5204667036	26.9359635658
50			
-135			
C	24.7601822577	51.4487482160	31.0027500172
H	25.3859514952	50.6176703972	31.3068735543
H	23.8389225144	51.0781991694	30.5809080148
C	25.5188437449	52.2796546118	30.0162928579
O	26.5801202600	52.8068485798	30.2544925772
C	30.2464603346	54.0918559019	28.4084435551
N	29.4939796821	52.3506276019	29.9996008517
C	30.3602770016	52.9437963767	29.0991374756
C	31.5431282422	52.0799824969	29.0661909160
C	31.3621376873	51.0492098509	29.8862146360
C	30.0248475158	51.1684372106	30.4893389584
C	29.3612792967	50.3269340495	31.3210016298
N	31.1626133010	49.0073952683	32.3911640731
C	29.9318446915	49.0902779218	31.8435300126
C	29.3261080819	47.7874299446	31.9343340210
C	30.1733920914	46.9576022810	32.5658869537
C	31.3945433359	47.7200615075	32.9138036314
O	32.3743621689	47.4033551821	33.5003918973
N	25.8849057805	55.4336023039	31.6511780739
C	25.0136036657	55.3082480229	32.7072528826
C	24.3591134422	56.6564525168	32.9176038160
C	24.7368519527	57.4676478986	31.6686429108
C	25.8316054057	56.6515200331	30.9966697404
O	24.8394259687	54.2974247670	33.3126295576
C	26.5602165513	57.0896754987	29.9393479353
N	28.2284970376	55.2851745172	29.3413524579
C	27.6348133038	56.5036112870	29.1848593904
C	28.3146982803	57.1565002188	28.1471462513
C	29.3211599479	56.3232275146	27.7067212738
C	29.2761122416	55.1502949622	28.4701333696
H	26.3142408768	58.0797775760	29.5998244128

H	28.3453082960	50.5536585887	31.5804005716
H	31.0524170938	54.2994726207	27.7366999252
H	27.9414531022	54.5829993702	29.9816864200
H	26.3772164671	54.6221362371	31.3491490862
H	31.7300614789	49.7861835445	32.6501386535
H	28.5117904832	52.5002826304	30.0159313461
H	23.8925393931	57.5621054862	30.9942062847
H	24.7574104753	57.0893340018	33.8281409884
H	24.5645886062	52.0524465054	31.8809048545
H	32.4128011355	52.3079285656	28.4837122139
H	32.0449105572	50.2482137499	30.0785232521
H	28.3586155071	47.5529398522	31.5389834688
H	30.0464678156	45.9216834549	32.8029070389
H	23.2942689569	56.5307966104	33.0550815968
H	25.0807495326	58.4659554306	31.9020196808
H	28.0907402738	58.1446450229	27.7979353284
H	30.0355077486	56.5163426633	26.9340981120
O	24.9383812981	52.4059115932	28.8517023856
H	25.4716832017	52.9486264241	28.2766205243
50			
-130			
C	24.7839756718	51.4275871267	30.9806230972
H	25.4051408679	50.5990047607	31.2995703917
H	23.8905734702	51.0547123777	30.5044259185
C	25.5781603455	52.2907577534	30.0513882368
O	26.6301843014	52.8080225745	30.3443610614
C	30.1735032215	54.0490075495	28.3469096890
N	29.5427706294	52.4175410395	30.1004089942
C	30.3284503591	52.9315641303	29.0833748559
C	31.4692896700	52.0203250072	28.9687192303
C	31.3503542631	51.0503857691	29.8703186192
C	30.0609388828	51.2167076608	30.5549805939
C	29.3924055357	50.3431257383	31.3420606689
N	31.1139019778	49.0400230445	32.5519817570
C	29.9699980940	49.1028911269	31.8463107287
C	29.4109820951	47.7778905856	31.7869526744
C	30.2128024883	46.9404225170	32.4660911971
C	31.3302719255	47.7330174335	33.0363530440
O	32.2053882119	47.4265228282	33.7685860009
N	25.9110089982	55.4554724784	31.6753913923
C	25.0392873696	55.3245549617	32.7302198912
C	24.3557031417	56.6614163540	32.9198864754
C	24.7264225896	57.4672334128	31.6647573219
C	25.8350663563	56.6620202894	31.0030304741

O	24.8868009491	54.3188847612	33.3488893760
C	26.5506432999	57.0968452298	29.9363580974
N	28.2076354197	55.2865241756	29.3348471302
C	27.6319724640	56.5135559094	29.1879854682
C	28.3185285881	57.1640559820	28.1555363516
C	29.3065100706	56.3162135151	27.6993313544
C	29.2429386422	55.1353914420	28.4490477730
H	26.2935291137	58.0813932964	29.5904391483
H	28.3527652548	50.5128346989	31.5541511513
H	30.9122527261	54.1930392948	27.5867532400
H	27.8780312328	54.5699786384	29.9379096340
H	26.4432711671	54.6588469503	31.4063955261
H	31.6137705055	49.8292279087	32.9092436331
H	28.5736453414	52.6038805898	30.2032463119
H	23.8830903211	57.5432741232	30.9871769820
H	24.7390534628	57.1106930627	33.8291127307
H	24.5345938581	52.0093478388	31.8602382700
H	32.2591667778	52.1505383200	28.2564904550
H	32.0341326510	50.2503155739	30.0613887097
H	28.5057474968	47.5464623177	31.2644376306
H	30.1097907811	45.8846560274	32.6188236839
H	23.2925235650	56.5157588761	33.0509443914
H	25.0544879175	58.4727463625	31.8914562302
H	28.1057708612	58.1554691574	27.8115539437
H	30.0239142937	56.5092203068	26.9292995250
O	25.0380557574	52.4577683873	28.8721686830
H	25.5919465956	53.0157767713	28.3326316927
50			
-125			
C	24.7898042596	51.4231561515	30.9759587709
H	25.4102404444	50.5952766546	31.2980017492
H	23.9019299562	51.0499551417	30.4898734354
C	25.5903444594	52.2931642336	30.0586965510
O	26.6382266589	52.8102058383	30.3650459238
C	30.1596967863	54.0429496717	28.3341531054
N	29.5405699093	52.4144211394	30.0945561384
C	30.3137773709	52.9203539041	29.0658387118
C	31.4480219312	52.0049958206	28.9419084106
C	31.3344104107	51.0339567330	29.8441929748
C	30.0604364710	51.2161262985	30.5535699765
C	29.4030657882	50.3668731937	31.3759326042
N	31.1006883071	49.0462351861	32.6003886036
C	29.9803096792	49.1138271998	31.8644018478
C	29.4406738363	47.7823927776	31.7528911900

C	30.2209642855	46.9405318349	32.4488032699
C	31.3050646006	47.7318186183	33.0763373289
O	32.1654554037	47.4128926090	33.8230099350
N	25.9164278854	55.4599679537	31.6807020868
C	25.0449876039	55.3276178805	32.7341771430
C	24.3550292833	56.6616130182	32.9205696921
C	24.7235063274	57.4670602701	31.6644796015
C	25.8359777506	56.6652481934	31.0037554964
O	24.8962258752	54.3227011187	33.3561414956
C	26.5489248170	57.0987679900	29.9375500193
N	28.2018314797	55.2853319018	29.3318891766
C	27.6324763781	56.5141822888	29.1865145860
C	28.3190358055	57.1642341201	28.1570211020
C	29.3038618011	56.3124674725	27.6981225908
C	29.2375732230	55.1307697439	28.4431213237
H	26.2891392688	58.0812669684	29.5878244950
H	28.3786460328	50.5656413376	31.6279690968
H	30.8970628978	54.1886670679	27.5717731935
H	27.8641732231	54.5675525240	29.9281594472
H	26.4538557309	54.6661031822	31.4148732655
H	31.5742780740	49.8316504809	32.9994162825
H	28.5755336199	52.6110930945	30.2163076321
H	23.8802298643	57.5400244269	30.9866089005
H	24.7360129345	57.1140702475	33.8291746992
H	24.5308118493	52.0018436666	31.8547891061
H	32.2330127241	52.1310006019	28.2246447247
H	32.0131380868	50.2253415938	30.0148720387
H	28.5767183504	47.5469089646	31.1652629831
H	30.1298944347	45.8794995151	32.5594509681
H	23.2923378477	56.5118696598	33.0510935860
H	25.0484788300	58.4736813595	31.8902266098
H	28.1102055375	58.1569650018	27.8148166812
H	30.0206163247	56.5051679359	26.9274810114
O	25.0604508101	52.4659118274	28.8756683650
H	25.6170143503	53.0295516228	28.3445415102
50			
-120			
C	24.7917859206	51.4220194603	30.9743888734
H	25.4123173008	50.5945809856	31.2970113212
H	23.9055645745	51.0489994748	30.4851293218
C	25.5949170325	52.2933847701	30.0607436765
O	26.6418604246	52.8091719665	30.3713118096
C	30.1565912196	54.0434131341	28.3293606138
N	29.5305143987	52.3959778349	30.0662159318

C	30.3044468361	52.9050620208	29.0456519142
C	31.4371935332	51.9923522603	28.9205624540
C	31.3274290156	51.0216143600	29.8268695503
C	30.0565000890	51.2075644186	30.5459983669
C	29.4093834961	50.3893447050	31.4061879234
N	31.0696186825	49.0482780402	32.6466122719
C	29.9749918837	49.1186165997	31.8847470818
C	29.4533023909	47.7789373855	31.7347349330
C	30.2163235657	46.9351375050	32.4498607923
C	31.2922445501	47.7235773062	33.0950410548
O	32.1565113674	47.4011908119	33.8348620550
N	25.9176496124	55.4610586687	31.6823373251
C	25.0466630878	55.3284564856	32.7350471149
C	24.3548804600	56.6617297230	32.9205924428
C	24.7227964051	57.4671521177	31.6641592091
C	25.8360727116	56.6662831837	31.0034524120
O	24.8988857281	54.3241505203	33.3587791393
C	26.5473090369	57.0992789885	29.9377790884
N	28.2003433046	55.2862995633	29.3314324358
C	27.6341832341	56.5147786932	29.1856236245
C	28.3187587586	57.1645680677	28.1575996393
C	29.3030618992	56.3105181819	27.6967110192
C	29.2378709503	55.1287765657	28.4409095644
H	26.2867178334	58.0812386920	29.5881518813
H	28.4034153409	50.6223478394	31.7027725164
H	30.8931663371	54.1942071502	27.5663684329
H	27.8592750360	54.5696453948	29.9270252669
H	26.4576320327	54.6688828555	31.4175884639
H	31.5546483297	49.8385777639	33.0177889983
H	28.5766264282	52.6210254466	30.2218602536
H	23.8791986983	57.5392433202	30.9867489091
H	24.7356073823	57.1145467871	33.8292003232
H	24.5296288798	51.9998801743	31.8528800278
H	32.2374028408	52.1434759517	28.2247565419
H	32.0119148363	50.2147671913	29.9867662040
H	28.6221975398	47.5325190031	31.1069708838
H	30.1450234302	45.8706309739	32.5356968926
H	23.2922893214	56.5106264473	33.0509229577
H	25.0464001058	58.4741966596	31.8901612483
H	28.1091888304	58.1577370372	27.8167200845
H	30.0206147116	56.5056190334	26.9264690860
O	25.0671937725	52.4679239111	28.8769049197
H	25.6230364230	53.0335406059	28.3470925852

50

-115

C	24.7929222245	51.4217779064	30.9737997421
H	25.4135132012	50.5943469913	31.2964003283
H	23.9075939132	51.0488742767	30.4828340234
C	25.5964970854	52.2934999416	30.0613533292
O	26.6441327702	52.8085037268	30.3735299220
C	30.1512560416	54.0424271156	28.3222949096
N	29.5271043260	52.3840102588	30.0528759810
C	30.3056557901	52.9050251187	29.0429660070
C	31.4371755446	51.9961349856	28.9174264420
C	31.3161596039	51.0102108109	29.8080811207
C	30.0456627541	51.1902960497	30.5226314782
C	29.4167968988	50.3947821337	31.4175205103
N	31.0330746968	49.0546711387	32.6914295738
C	29.9669809870	49.1180799000	31.8969543930
C	29.4667089482	47.7700159420	31.7112506168
C	30.2227261982	46.9298898958	32.4340342489
C	31.2808756880	47.7215142082	33.1085828713
O	32.1556150057	47.3992036810	33.8342797333
N	25.9178960061	55.4612336597	31.6831092124
C	25.0476918209	55.3291561157	32.7349012948
C	24.3550167929	56.6618084571	32.9204645169
C	24.7222597840	57.4673670897	31.6644431494
C	25.8357674019	56.6672892350	31.0035965770
O	24.8997942333	54.3245322044	33.3602137681
C	26.5452151019	57.0993573569	29.9379475849
N	28.2020917749	55.2874564244	29.3320079465
C	27.6340674583	56.5135797435	29.1833403089
C	28.3177660507	57.1634811910	28.1579043266
C	29.3051263001	56.3097404448	27.6988517411
C	29.2381158230	55.1278283552	28.4381708916
H	26.2856007196	58.0820490680	29.5889373744
H	28.4319128881	50.6552663565	31.7545652469
H	30.8911397092	54.1954812520	27.5626933383
H	27.8577496693	54.5727124044	29.9267319837
H	26.4597481747	54.6701342794	31.4192982928
H	31.5522107883	49.8464992943	33.0149451290
H	28.5819366664	52.6268216672	30.2263531417
H	23.8791944395	57.5386080632	30.9866333226
H	24.7352058866	57.1147427682	33.8290338931
H	24.5288404974	51.9987773137	31.8523531949
H	32.2387184954	52.1478997208	28.2230218375
H	32.0149447998	50.2192580651	29.9847791551
H	28.6341894327	47.5220316638	31.0832461191

H	30.1473324445	45.8654703269	32.5360668867
H	23.2927114098	56.5103486919	33.0506997737
H	25.0457202718	58.4743715783	31.8900118720
H	28.1085248607	58.1571411552	27.8186712920
H	30.0192593133	56.5058210456	26.9266130728
O	25.0699125658	52.4684067876	28.8770851609
H	25.6250363219	53.0349701754	28.3476228012
50			
-110			
C	24.8195088673	51.4085969232	30.9574190923
H	25.4411218458	50.5858152757	31.2895308353
H	23.9518384070	51.0303802102	30.4396584598
C	25.6388386893	52.3009534823	30.0792337647
O	26.6721874319	52.8186035196	30.4289616606
C	30.1310835880	54.0360052580	28.3109626048
N	29.5374018259	52.4061810073	30.0763870786
C	30.2790964025	52.8896879069	29.0208805605
C	31.3682684305	51.9424453591	28.8432062581
C	31.2718773191	50.9775943908	29.7614262398
C	30.0510685329	51.2110706722	30.5392837723
C	29.4443638456	50.4256275097	31.4575523792
N	31.0303343515	49.0642449033	32.7613449875
C	30.0151989129	49.1477302500	31.9058406618
C	29.5448296082	47.8027913601	31.6375977607
C	30.2432160604	46.9430157259	32.3918911319
C	31.2302417525	47.7193499078	33.1810475897
O	32.0240991985	47.3818505720	33.9875896548
N	25.9324178928	55.4720253239	31.6971289195
C	25.0646488579	55.3356391944	32.7500197244
C	24.3551542905	56.6608113623	32.9268148402
C	24.7150808865	57.4652778300	31.6671050221
C	25.8315743128	56.6697577771	31.0043525317
O	24.9288697909	54.3338757711	33.3816662847
C	26.5300463121	57.0975285819	29.9308535785
N	28.1951926495	55.2912533806	29.3334584071
C	27.6283943539	56.5171532727	29.1813448746
C	28.3125640110	57.1653838810	28.1574673481
C	29.2997101750	56.3084671606	27.6985428683
C	29.2324867741	55.1296159789	28.4389950372
H	26.2545823740	58.0705118169	29.5677634275
H	28.4616295399	50.6742592024	31.8099025997
H	30.8491079740	54.1706584371	27.5276398377
H	27.8351873568	54.5696629841	29.9108626365
H	26.4791573364	54.6840268689	31.4356787223

H	31.4674451298	49.8452107057	33.2115435785
H	28.6042874046	52.6693423699	30.2871985103
H	23.8689145879	57.5344819471	30.9930643327
H	24.7269352503	57.1232634383	33.8342292109
H	24.5260927856	51.9744491631	31.8338818807
H	32.1264811791	52.0479981472	28.0948404019
H	31.9347449961	50.1474681381	29.8957099265
H	28.7722575693	47.5832798331	30.9296454408
H	30.1682026768	45.8762065650	32.4411560715
H	23.2944001209	56.4958642026	33.0555765186
H	25.0364277497	58.4732171023	31.8917629574
H	28.1028784252	58.1566865728	27.8118629821
H	30.0164439796	56.5038661125	26.9279935474
O	25.1444320598	52.4917500933	28.8836314775
H	25.7108257076	53.0719685876	28.3810514481
50			
-105			
C	24.8211006191	51.4076018839	30.9558453252
H	25.4446674332	50.5864761467	31.2878435473
H	23.9541927156	51.0278176140	30.4381139917
C	25.6422084265	52.3007754103	30.0797713486
O	26.6744123657	52.8157158076	30.4327960121
C	30.1280411125	54.0343685614	28.3065539521
N	29.5313957720	52.3924322426	30.0607769064
C	30.2749429724	52.8827097897	29.0112658424
C	31.3617054464	51.9378275643	28.8319396226
C	31.2574311417	50.9640869135	29.7424913928
C	30.0437794034	51.2002906338	30.5239454896
C	29.4554924537	50.4359669538	31.4757535686
N	30.9909724088	49.0664619347	32.8052174368
C	30.0080602199	49.1483255568	31.9174508970
C	29.5612890554	47.7964853019	31.6145096995
C	30.2491925092	46.9389900912	32.3801642818
C	31.2174768701	47.7192850650	33.1932131655
O	32.0173116288	47.3787433326	33.9903018527
N	25.9328453018	55.4726030862	31.6985047071
C	25.0658353201	55.3354698041	32.7515009499
C	24.3551010493	56.6606698662	32.9274899582
C	24.7144191506	57.4649615798	31.6675989402
C	25.8312256038	56.6701087685	31.0044219985
O	24.9303840396	54.3351827317	33.3839091930
C	26.5279303059	57.0978167846	29.9306095270
N	28.1990214767	55.2950678136	29.3366213408
C	27.6285951541	56.5178422504	29.1798457202

C	28.3103838597	57.1647406300	28.1568121216
C	29.3016475657	56.3088826797	27.7002004523
C	29.2358816067	55.1320599600	28.4400992126
H	26.2504707318	58.0697579496	29.5661182452
H	28.4936054324	50.7111590623	31.8694979013
H	30.8483704024	54.1733074858	27.5242471841
H	27.8328111742	54.5722662174	29.9075883500
H	26.4802896184	54.6849122355	31.4362863053
H	31.4578761170	49.8513740119	33.2147437698
H	28.6094533195	52.6765545128	30.2900147998
H	23.8680543457	57.5346527582	30.9940649564
H	24.7262721330	57.1240166830	33.8345965651
H	24.5264313594	51.9734420578	31.8319330558
H	32.1261369485	52.0537054614	28.0907296496
H	31.9311948419	50.1456359777	29.8885467322
H	28.7962037489	47.5759541510	30.8973089030
H	30.1719012881	45.8709641357	32.4369676543
H	23.2945700355	56.4946049015	33.0561883563
H	25.0356174487	58.4728031899	31.8922100568
H	28.1008896539	58.1556603471	27.8118047603
H	30.0154364524	56.5032144101	26.9268141529
O	25.1500883275	52.4938973261	28.8832267879
H	25.7185332124	53.0752564040	28.3841027969
50			
-100			
C	24.8437194772	51.4046586801	30.9472418365
H	25.4650718636	50.5831108806	31.2828541387
H	23.9917075093	51.0273107924	30.4038983936
C	25.6706137255	52.3114046024	30.0918781784
O	26.6905048917	52.8389635090	30.4644342021
C	30.1248866153	54.0353023907	28.3088360338
N	29.5195416625	52.3729353277	30.0378257002
C	30.2630243964	52.8691533600	28.9914937091
C	31.3290706734	51.9087394263	28.7877061851
C	31.2202427623	50.9258202250	29.6906846561
C	30.0358338676	51.1882355657	30.5047677861
C	29.4708419026	50.4579160365	31.5033242056
N	30.9799029871	49.0731839099	32.8728129868
C	30.0293338329	49.1803598450	31.9540010258
C	29.5931918333	47.8355397725	31.6019744855
C	30.2607915900	46.9541357426	32.3532804114
C	31.1961532872	47.7096264269	33.2287336469
O	31.9603140007	47.3480656817	34.0493766549
N	25.9390472004	55.4783285078	31.7014205045

C	25.0772351201	55.3378656849	32.7568632395
C	24.3568679826	56.6577168303	32.9313986041
C	24.7102679457	57.4646864058	31.6709360275
C	25.8265475412	56.6732362717	31.0034304231
O	24.9520968962	54.3374175565	33.3939554890
C	26.5148433051	57.0962067535	29.9236860625
N	28.1783796471	55.2858749730	29.3240122343
C	27.6140057574	56.5117029912	29.1706777175
C	28.2996613482	57.1600508621	28.1539278869
C	29.2952984628	56.3043835859	27.7017019399
C	29.2256032198	55.1274351437	28.4371522205
H	26.2402587258	58.0688282704	29.5590647246
H	28.5304488190	50.7596774218	31.9213781755
H	30.8570203550	54.1874664112	27.5406148672
H	27.8318455530	54.5732499674	29.9196688744
H	26.4927436751	54.6958593764	31.4402317435
H	31.3918227801	49.8417596023	33.3665093485
H	28.6115845176	52.6774716382	30.2991607487
H	23.8614547652	57.5320749152	31.0002780812
H	24.7234128542	57.1233019053	33.8391698223
H	24.5266657837	51.9600011870	31.8223313647
H	32.0886641318	52.0187336176	28.0404903436
H	31.8631187247	50.0755739824	29.7918446467
H	28.8589547373	47.6428179299	30.8447057769
H	30.2034687512	45.8838952682	32.3498607564
H	23.2974538787	56.4840124912	33.0582933575
H	25.0295659133	58.4734967394	31.8948085566
H	28.0908620459	58.1515974378	27.8103343583
H	30.0172432753	56.5037585903	26.9373767481
O	25.1945109106	52.4986374017	28.8881270786
H	25.7554480771	53.0913241423	28.3940234784
50			
-95			
C	24.8469157277	51.4050862726	30.9459216978
H	25.4666312227	50.5846476566	31.2808529309
H	23.9984762900	51.0321156834	30.3968137411
C	25.6749400708	52.3122317926	30.0954887807
O	26.6934053457	52.8412078063	30.4673751985
C	30.1238744612	54.0352420333	28.3100832434
N	29.5116220276	52.3570613216	30.0230045487
C	30.2617918052	52.8665183675	28.9853789213
C	31.3262004735	51.9083049439	28.7806749533
C	31.2041004051	50.9126709164	29.6699733831
C	30.0223927511	51.1722592792	30.4820622479

C	29.4812293256	50.4633317828	31.5131324613
N	30.9411235343	49.0760146699	32.9106044428
C	30.0212637730	49.1853525904	31.9671906623
C	29.6100254445	47.8326980109	31.5844127805
C	30.2713385002	46.9501745896	32.3372832470
C	31.1836082834	47.7086052306	33.2381174494
O	31.9585056877	47.3461697482	34.0464476024
N	25.9390882038	55.4795815577	31.7001928929
C	25.0785595019	55.3398599845	32.7552999359
C	24.3567787820	56.6588436718	32.9313583268
C	24.7104823459	57.4649916728	31.6712896189
C	25.8257314304	56.6739930617	31.0025774981
O	24.9546422152	54.3399483882	33.3927838403
C	26.5128641280	57.0939314817	29.9233575564
N	28.1752997214	55.2850890140	29.3199693487
C	27.6122285018	56.5109519876	29.1681664985
C	28.2964945299	57.1590786379	28.1545890359
C	29.2940921069	56.3027358627	27.7015483135
C	29.2230079681	55.1277805805	28.4334446979
H	26.2402059855	58.0661693574	29.5621462863
H	28.5706270006	50.7958323477	31.9714208979
H	30.8581112556	54.1910150866	27.5458148626
H	27.8348583802	54.5765109597	29.9229093793
H	26.4953520827	54.6983510950	31.4409817703
H	31.3843327938	49.8455009778	33.3780626510
H	28.6146852135	52.6798319661	30.2989045256
H	23.8615295168	57.5293463878	31.0019585647
H	24.7235521791	57.1223964388	33.8401130825
H	24.5269932847	51.9584848676	31.8188075046
H	32.0934173656	52.0229850722	28.0419570338
H	31.8569647235	50.0733404060	29.7835500000
H	28.8732054694	47.6425647110	30.8301974852
H	30.2058460004	45.8781776960	32.3437874873
H	23.2975385532	56.4823212517	33.0578521504
H	25.0299294130	58.4730328498	31.8951618414
H	28.0889968002	58.1506344384	27.8127639518
H	30.0177821173	56.5044042781	26.9401065162
O	25.1965339597	52.4956282543	28.8916193298
H	25.7539729205	53.0898989977	28.3950482604
